# Colour-Balanced Edge-Guided Digital Inpainting: Applications on Artworks

**DOI:** 10.3390/s21062091

**Published:** 2021-03-17

**Authors:** Irina-Mihaela Ciortan, Sony George, Jon Yngve Hardeberg

**Affiliations:** Department of Computer Science, NTNU—Norwegian University of Science and Technology, 2815 Gjøvik, Norway; sony.george@ntnu.no (S.G.); jon.hardeberg@ntnu.no (J.Y.H.)

**Keywords:** inpainting, colorization, generative adversarial networks, dunhuang wall paintings

## Abstract

The virtual inpainting of artworks provides a nondestructive mode of hypothesis visualization, and it is especially attractive when physical restoration raises too many methodological and ethical concerns. At the same time, in Cultural Heritage applications, the level of details in virtual reconstruction and their accuracy are crucial. We propose an inpainting algorithm that is based on generative adversarial network, with two generators: one for edges and another one for colors. The color generator rebalances chromatically the result by enforcing a loss in the discretized gamut space of the dataset. This way, our method follows the modus operandi of an artist: edges first, then color palette, and, at last, color tones. Moreover, we simulate the stochasticity of the lacunae in artworks with morphological variations of a random walk mask that recreate various degradations, including craquelure. We showcase the performance of our model on a dataset of digital images of wall paintings from the Dunhuang UNESCO heritage site. Our proposals of restored images are visually satisfactory and they are quantitatively comparable to state-of-the-art approaches.

## 1. Introduction

Image restoration is a classical task in computer vision, where the objective is to enhance the quality of an image, by removing noise, undoing irreversible damage, increasing the resolution, or recovering lost information. Inpainting, which is also known as image completion, is one of the instances of the image restoration problems that aims to fill gaps in a digital image, by reconstructing the color and structural elements. Colorization is highly related to inpainting, the task of hallucinating colors in black-and-white and neutral-toned visual material, where the semantics is preserved but the color clues are non-existent. As a matter of fact, the choice of possible infilling colors is more confined in image completion cases, since assumptions can be extrapolated from the area surrounding the missing region.

Centuries old artworks with historical and cultural values are among the surfaces that most suffer from severe degradations due to aging and mishandling. In most cases, the damage represents a complete lacuna, where ground-truth is missing and/or the number of unknowns is too high to allow the generation of a fully validated reconstruction. Retouching the physical objects might result in a risky operation and it is perceived with very careful and conservative views in the Cultural Heritage (CH) community. Nonetheless, the desire to visualize the hypothesis of how the undamaged original might have looked like is still present, and the possibility to do this digitally without altering the original is very attractive to art conservators. This places CH among the first applications to benefit from digital inpainting. As a matter of fact, the pioneering work on digital inpainting [1] was built around the scope of artwork reconstruction.

Traditional solutions to inpainting fill the missing region while using the statistics of the image and the structure and texture synthesis from the neighbourhood. However, such traditional approaches require ad-hoc feature engineering and they are seldom semantic-aware. The take-off of deep learning techniques has leveraged the performance of image restoration solutions, including inpainting. End-to-end convolutional neural network (CNN) models infer complex image understanding processes and they account for low-level characteristics and high-level semantics of the image in one-go without the need to break the image and feature computation into sub-components treated as single cases.

**Figure 1:**
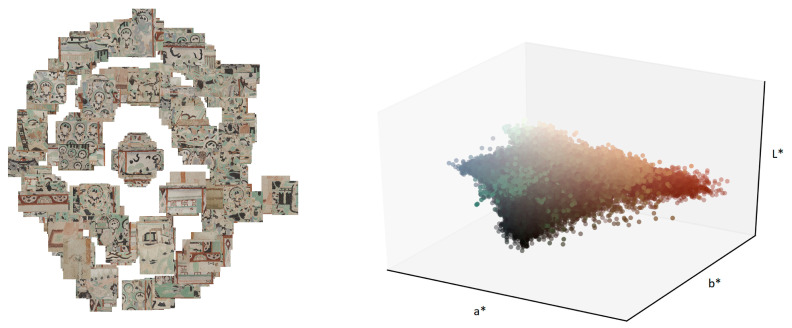
**Left**: Dunhuang dataset visualized in a two dimensional space with Barnes-Hut Stochastic Neighbour Embedding (BH-SNE) [8]. The clustering is done based on the activations of the first fully connected layer of the pretrained VGG19 network [9] that outputs a vector of 4096 features (color, size, semantics, etc.) for each image. Images are displayed exactly at their embedded location in the two-dimensional (2D) projection space. **Right**: color gamut of the Dunhuang dataset. The L*a*b* coordinates are rendered with the corresponding RGB colour.

The interest for virtually repairing damaged artworks with deep learning approaches is rising. As a matter of fact, the e-heritage Workshop of the International Conference on Computer vision in 2019 [2] organized an image restoration challenge for image inpainting that is tailored to CH scans, releasing a dataset of wall paintings (visualized in Figure 1 from the Dunhuang cultural site [3]). There are not numerous such attempts in the computer vision world, partly because CH datasets are yet not perceived as a baseline in the deep learning community and partly because they are not widespread. For this reason, most of the deep learning approaches are not made to solve the specific problems of art images. Moreover, most of the solutions for the CH field are deployed by means of transfer learning from approaches that were developed for natural images (ImageNet [4], Places [5]), buildings and streets (Paris Street View [6]), celebrities faces (CelebA [7]), etc.

In this work, we propose an inpainting algorithm for artworks, where two convolutional models are learned in a generative adversarial fashion: an edge generator and a color generator. The purpose of the first model is to learn the edges in the missing region based on a monochrome-only representation of the input image. To complement the structural information, the second model fills in the gap with color information. It does so by enforcing that the infilled chromatic information respects a balanced palette, based on priors that were computed in the quantized chromatic channels, so that the generator doesn’t get biased to only overly-used colors. With this workflow, we are traversing the usual steps that an artwork usually undergoes: first the under-drawings are sketched (edges), then the main color palette is chosen (priors), and, finally, the color tones are painted. We test our approach on digital images of the Dunhuang murals, part of an UNESCO heritage site in North China [3].

## 2. Related Work

In this literature review, we only focus on learning-based solutions to digital inpainting. We start with works on natural images, which are the state-of-the-art in field and, then, we carry on to cultural heritage applications.

### 2.1. Deep Learning Approaches for Image Inpainting

The seminal work of Pathak et al. [10] addressed the inpainting of natural images with an encoder-decoder CNN. The encoder part is based on AlexNet architecture [11], trained on an input image size of 227 × 227. The missing area amounts to 1/4 of the image and it is simulated by either a rectangular shaped mask originated at the center or several rectangular areas randomly positioned. Such solutions that involve rectangular masks are categorized as region inpainting. By adding the adversarial loss to the L2 Euclidean reconstruction function, [10] claim a substantially improved accuracy.

The approach of Iizuka et al. [12] is inspired by [10], while adding the following contribution: holes are simulated by arbitrary forms, as opposed to only rectangular shapes, a training process in the adversarial fashion that ensures multi-scale resolution and global as well as local consistency and applications to more challenging data, such as faces. In order to increase the quality of the resolution, they also use dilated convolution layers, which allows for increasing the receptive field without placing a burden on the number of parameters or the computational complexity. These methods are however dependent on the initialization of the missing pixels and might need post-processing refinements. The work of Liu et al. [13] proposes an end-to-end inpainting pipeline with partial convolutions arranged in a U-Net architecture, where convolutions affects only non-hole pixels and where the mask gets updated after each convolution, by getting thinner and thinner, until its total disappearance. This way, the content to be infilled is learned only from the contextual information provided by the valid pixels. Moreover, the masks have no constraints on the shape and the approach gives good results, even if the missing information is present around the edges (image extrapolation).

Isola et al. [14] and Shu et al. [15] both employ generative adversarial networks (GAN) to solve a more general problem, that of "image to image translation”, which can be adjusted to inpainting as well. In the former paper, the inputs are pairs of images, while the latter extends the work for cases when pairs of images do not exist by introducing a cycle consistency loss. The work of [16] offers multiple plausible solutions for the completion of natural images, faces and buildings and trains in parallel two networks (reconstructive and generative) supported by the generative adversarial setting.

Inspired by the image-making process from an artist’s perspective "lines first, color after”, the network that was proposed by Nazeri et al. [17] uses a three-stage GAN network: firstly, the edges are learned in the missing region from the grayscale input; secondly, the color information in the missing area is learned from the RGB input; and, thirdly, the joint edge and color information are trained in conjunction.

There is a body of works that focus on the accurate inpainting of complex structural information [18,19,20]. These works exploit landmarks in the geometry of the human body [18,20] and human faces [19] in order to estimate parsing maps. Parsing maps encode the components that define the objective structure, together with each part’s labeled location that constrain the subsequent color completion step. While these methods go beyond learning edge information, since they deal with a more specific and geometric definition of structure, they require a dataset that is uniform and deterministic in its shape representations, be it human body, animal body, or human faces. In this sense, the Dunhuang dataset is rich in content, varying from Buddha representations to architectural elements and decorative patterns [3].

### 2.2. Deep Learning Approaches for Paintings Retouching

One of the first attempts to address image restoration problem with a dedicated interest for paintings and for big missing regions was made by van Noord [21]. The method is mainly based on the context encoder of [10], but it uses dilated convolutions instead and different loss functions. In order to repair damaged wall paintings of the Dunhuang site, Yu et al. [22] utilize transfer learning from [13] and bring modifications that adapt to the specificity of the data, such as the use of masks that have a random walk configuration and, thus, resemble more the stochastic damage process of the wall paintings surface. More specifically, the dusk-like masks (based on random walk) emulate deterioration by molds and salty erosion, while jelly-like masks (which is a dilated random walk) simulate physical damages or sabotages.

Wang et al. [23] offer a method for the digital restoration of Tibetan Thanka murals, based on Unet Partial Convolution Network. Their main contributions are related to the mask and training loss design. Thus, they analytically summarize the frequent type of damage in the murals as scratches and spots and simulate the damage with masks that contain lines and elliptical shapes with various size parameters and of random distribution. These masks are similar to the irregular masks that were proposed by [12], while adding the elliptical elements as variation. Nonetheless, this mask design is tailored to the type of damage observed in the Thanka murals. Further on, they train their model in two stages: in the first stage, higher weight is given to losses that characterize per-pixel reconstruction, while, in the second stage, higher weight is given to perceptual losses.

In their work [24], Wang et al. identify the problems of low-resolution, color discrepancy, and blurriness in image inpainting for general applications, and they stress that these problems are more critical for CH surfaces. Thus, their methodology is rooted in the unpaired image-to-image translation implemented with a fully-convolutional CycleGAN architecture [15]. Nonetheless, they bring minor improvements to account for the above-mentioned problems. They address the high-resolution by diving the symmetrically-padded input images (8912 × 8912) into patches of the maximum size allowed by memory (2048 × 2048) and then recompose the patches into a high-resolution image. However, this results in a loss of local color accuracy that is compensated by adding a color constraint to the high-resolution mosaic. This constraint is derived from a low resolution reconstruction with the same network, where the input image is resized to the maximum possible size (2048 × 2048). The constraint is verified by means of an identity loss. As far as the sharpness is concerned, it is solved with a Gaussian–Poisson editing in the post-processing stage.

Weber et al. [25] also include refinements in the post-processing stage, by inserting human expertize in their inpainting solution to the Dunhuang murals and benchmark dataset [3]. More precisely, they introduce an interactive extension to the Deep Image Prior (DIP) work [26], where an initial restoration is given by the DIP method, that requires no training process. Through an interactive tool, the initial restoration is then edited by human experts, which generates an improved result that is further fed back into the DIP algorithm. This process continues iteratively until the user is satisfied with the inpainted image. While such an interactive approach is very attractive, especially because of bridging the domain-specific expert knowledge with the input provided by computer vision technologies, this work does not challenge the internal learning mechanisms of a deep-learning model.

### 2.3. Research Gaps and Contributions

There is still room for improvement for deep learning approaches for artworks inpainting. Many of the imperative improvements to be made are on color consistency and high-resolution. For strengthening the color consistency, new losses have been formulated in the GAN setting, such as identity loss, Wasserstein loss [27], etc. A successful approach was developed by Zhang et al. [28] for the recolorization of natural images, where the color accuracy is not verified with an Euclidean distance (that is minimized by the mean value), but formulated as multinomial classification, where the ground-truth image’s gamut is represented in a quantized L*a*b* color space. Moreover, each of the discrete values of the quantized color space receives rebalancing weights to account for the natural images’ bias to desaturated colors (due to the higher frequency of skies and landscapes in the dataset). A similar color-palette constraint was also used in [29], without the rebalancing weights. Even though these color-palette constrained CNN have been developed for the recolorization tasks, they can be easily adopted in the inpainting task.

Several actions can be taken for reaching sharper results and higher-definition. If computing power allows, training on bigger image size can help. Otherwise, the result of the inpainting can be improved by being fed to super-resolution model. The resolution is increased when the receptive field of a CNN is increased, so dilated convolutions are good practice, because they expand the receptive field without an echo on the number of the parameters included in the model [21].

In addition, the size of the receptive field is also affected by the size and shape of the holes. As a matter of fact, in [30], they studied how the size, shape, and orientation of masks influence the performance of digital inpainting.

Based on all of these insights, we bring the following contributions in our proposed model: (1) we complement the edge-guided multi-stage network introduced by [17] with a color-aware loss that rebalances the chromatic elements to avoid the bias of dull colors from the core of the gamut; (2) we use four morphological variations of the random-walk mask, so as to target different receptive fields of the network. While the work of Nazeri et al. [17] is guided by the principle “lines first, color after”, our work’s underlying principle is “lines first, color palette after, color tones at last” by resembling even more the modus operandi of an artist.

## 3. Method

**Figure 2:**
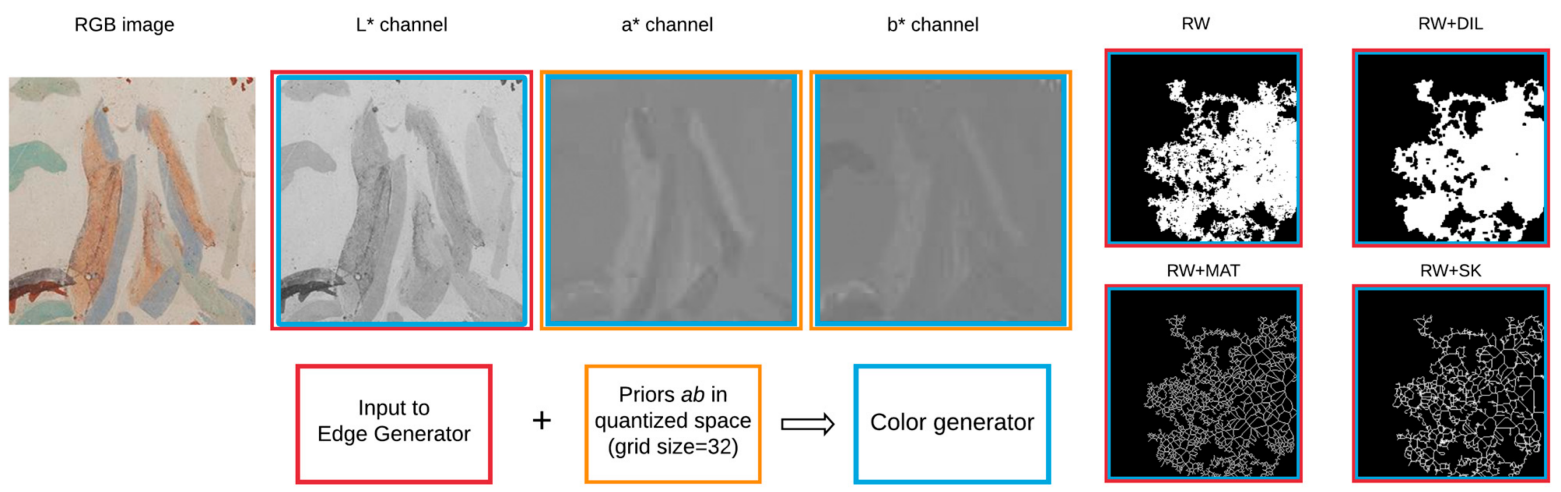
Our model converts the RGB images to L*a*b* color. The edge generator receives as input only the monochrome luminance channel and the binary masks of missing pixels (that follow variations of a random walk pattern). Subsequently, color rarity weights are computed on the a* and b* channels and then used in the loss function of the color generator. The edge map, the multichannel L*a*b* image and the priors are fed to the color generator.

**Figure 3:**
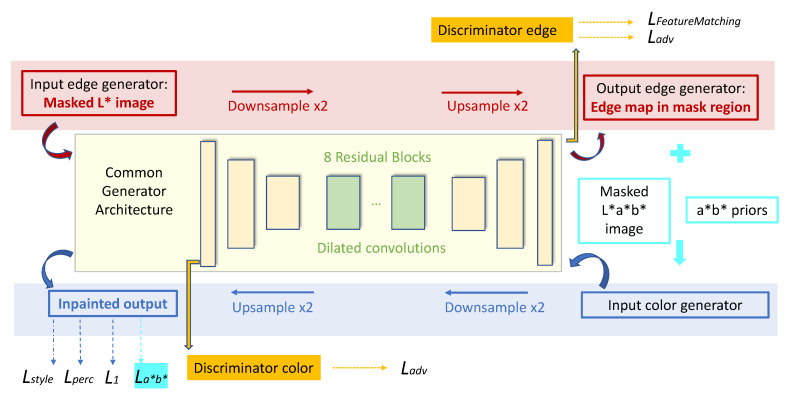
Both color and edge generators have the same underlying convolutional architecture, composed of encoder–decoder blocks and eight residual blocks with dilated convolution in between. Different from [17], we introduce the $L_{a*b*}$ loss for the color generator to lower the bias towards mean values of the dataset’s gamut.

Our approach to solve inpainting for artworks is both edge and color aware. Building on the work of [17], we train two generative adversarial networks (see the diagram of our approach in Figure 2): one that learns the edges in the lacunae (Section 3.2) and a second one that learns the color information (Section 3.3). The two networks are trained in a multi-stage fashion: first, separately and then combined, as detailed in in Figure 3. Contrary to the work of [17], instead of the RGB colour space, we work in the L*a*b* space, which is a more perceptual chromatic space. Besides enforcing the computation of the convolutional neural features in a perceptual space, the use of L*a*b* space serves the scope of rebalancing colors, so that they fit into the overall palette of the dataset. In order to improve the color coherence, we smooth the effect of L1 loss in GANs—that of filling in empty spaces with the mean colour of the gamut, which results in inaccurate and desaturated colors—by adding a color rebalancing loss similar to [28,31].

### 3.1. Masks

**Figure 4:**
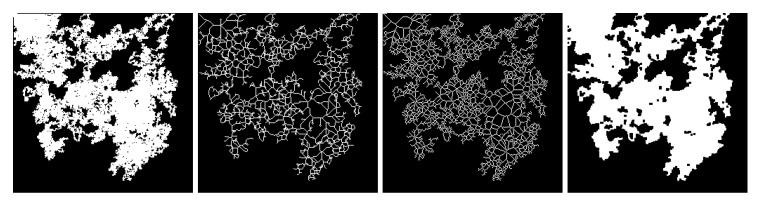
Random walk (RW) mask (leftmost) with three morphological variations: skeletonization (RW + SK), medial axis transform (RW + MAT) and dilation (RW + DIL). Besides covering different areas of missing pixels, these masks simulate various patterns characteristic to artwork degradation: moist and pest formation, craquelure, and mechanical damage.

The performance of learned image completion is influenced by the network activations and the receptive field, especially when training for a resolution twice or four times the test image resolution. Based on this insight, we take the following measures to modulate the size of the receptive field: we incorporate dilated convolutions and we use irregular masks of different size and orientations. We start with a mask that simulates a random walk: starting from a random seeding position, each next move is decided by a random choice between the pixels in the four-connected neighbourhood. There can be multiple seeding positions and, at the same time, a pixel can be re-traversed multiple times, which decides the overall spread of the mask. We use the convention 1 values for holes, 0 values for non-hole region. Each base random walk mask is then further processed into morphological derivates (see Figure 4) by applying the following operations: dilation, skeletonization and medial axis transform. The random walk mask was proposed in the Dunhuang challenge [2,3]. This pattern was adopted in [22], where the authors call the normal random-walk dusk-mask and the dilated random walk jelly mask, claiming the former to be characteristic of mold and erosion and the latter of physical damages. Nonetheless, to the best of our knowledge, we are the first to introduce the skeletonization and medial axis transforms of the random walk. Through these operations, we are synthesizing a fine craquelure-like structure, a common aging effect of artworks.

### 3.2. Edge Inpainting Model

The edge model receives three inputs: the Luminance channel of the ground-truth data, the mask image, and the edge map obtained with the Canny operator from the luminance channel. The threshold for Canny edge detection was empirically set to 1.1 to discount as much as possible noise and only select the relevant edges. As sketched in Figure 3, we preserve the network architecture that was proposed in [17] with dilated convolutions and residual blocks, followed by spectral normalization. Beside the adversarial loss, the loss function for the edge hallucinator includes feature-matching loss that compares activation maps at intermediate layers of the discriminator.

### 3.3. Color Inpainting Model

The input to the color inpainting model is the masked L*a*b* image and the edge map. In a first phase, when the color model is trained independently from the edge model, the edge map is given by the Canny operator. Subsequently, when the two models are jointly trained, the edge map is inferred by the edge model. The architecture of the network is the same as in [17], consisting of dilated convolutions and residual blocks, followed by instance normalization. As in [17], the loss function for the generator includes the adversarial loss, the $L_{1}$ distance, the perceptual loss, and the style loss $L_{1},L_{adv},L_{perc},L_{style}$. In addition, we inserted a new loss term, $L_{ab}$, which only operates on the chromatic channels.

The $L_{ab}$ loss is computed as multi-class cross-entropy in the quantized *ab* space between the target and predicted image, multiplied by color rebalancing priors. The priors are extracted before the training process, by saving the discretized *ab* space (where number of quantiles *q* = 32) for each training image. Subsequently, these priors are smoothed with a Gaussian filter and finally, they are mapped to a probability distribution between [0,1] in the discretized space of possible colors. This loss is computed separately for a and b channel, and then averaged as the final $L_{ab}$ loss that contributes to the generator loss. The full mathematical formulation for the color rebalancing loss can be followed in Equations (1)–(4). The parameter *p* was chosen as in [31]. The weight for the losses are as follows: $w_{L_{1}}=0.05,w_{L_{adv}}=0.1,w_{L_{perc}}=0.1,w_{L_{style}}=250,w_{L_{ab}}=0.9$. We did not completely exclude the $L_{1}$ distance, instead we opted for a very small weight, since we want the model to still be aware of the global differences that are provided by $L_{1}$ norm.

(1) $$priors_{a,b}={\left(\left(1-p\right)\left(Filt_{gaussian}\times q_{train}\right)+p\right)}^{-1}$$

(2) $$l_{a,b}=priors_{a,b}*MCE\left(q_{a,b}\left(target\right),q_{a,b}\left(pred\right)\right)$$

(3) $$L_{ab}=mean\left(l_{a},l_{b}\right)$$

(4) $$\begin{array}{c} Loss_{GC}=w_{L_{1}}L_{1}+w_{L_{adv}}L_{adv}+w_{L_{style}}L_{style}+\\ w_{L_{perc}}L_{perc}+w_{L_{ab}}L_{ab} \end{array}$$

Finally, the output of the network is rendered from L*a*b* to RGB and merged with the non-masked original pixels.

## 4. Results

### 4.1. Dataset and Training Specifications

Our model was trained and tested for the Dunhuang dataset, released for the ICCV e-Heritage workshop challenge [3]. The images represent digital scans of the mural paintings inside one of the Mogao caves in North China. Generally, the Dunhuang caves were painted across many centuries by various artists and under various dynasties, covering more than one artistic style. They display figurative symbols that were taken from Buddhist mythology. The ICCV e-Heritage challenge released 500 train images with ground-truth and 100 masked test images, for which the reference has not yet been released to the best of our knowledge. The images have non-uniform dimensions across the dataset. Therefore, for our work, we split the available 500 images into 465-10-25 images as base for training-validation-test experiments. Further on, we cropped the test and validation images in blocks of 256 × 256 with minimal overlap, so as to take advantage of the full resolution of the images. This totalled to 5596 ground-truth images for training and 96 for validation. The test images were left at their original size.

Each image in the training and validation collections was accompanied by a random walk mask and its 3 morphological variations, so the total training load was 22,384 images. Meanwhile, for each image in the test set, two base masks plus the derivates were generated, amounting to a total of 200 images for inference. The masks cover from 0 to 60% of the image size. The training images were randomly flipped horizontally and vertically with a random factor that is decided by a binomial distribution.

**Figure 5:**
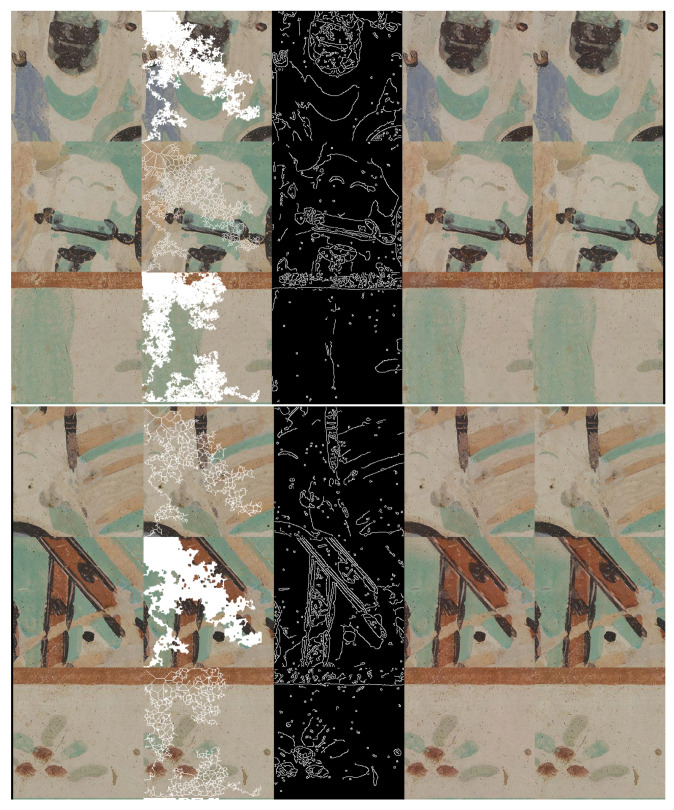
Intermediate results generated for a subset of random six images from the validation set (original size of the images is 256 × 256, scaled to fit in the page). For each of the six instances, five images are shown, in order from left to right: ground-truth; ground-truth with deterioration; edge map in the missing region; output inpainted image with both edge and color information as generated by the network; and, output of the model merged with the non-masked input pixels.

The model was implemented in PyTorch1.1 [32] and then trained on a single CUDA-enabled GPU with a memory capacity of 11 GB. Training was performed on images of size 256 × 256, with batch size of 4. The model was optimized with ADAM optimizer [33], and the hyperparameters $\beta_{1}$ and $\beta_{2}$ set to 0 and 0.9. We followed the training strategy of [17]. In a first stage, the color generator was trained separately from the edge generator and then, the input from the edge model was added as input to the color-only based model to improve the infilling of edges. For the separate training phase of the generators, we set the learning rate to $10^{-4}$ and stopped the training after observing a flatness in the oscillation of the loss values. Afterwards, for the joint training, we reduced the learning rate to $10^{-5}$ to push the weights of the model to update for smaller changes, and continued training until the losses plateaued. In each case, the discriminators were learned at a rate that was ten times lower than the generators’ rate. Figure 5 displays the intermediate results of the jointly trained model on the Dunhuang validation dataset. An interesting highlight of these intermediate maps are the edge maps, which show how the edge generator is able to infill structure in the missing regions in a way that follows the edge lines in the original image.

### 4.2. Qualitative and Quantitative Assessment

The test images were reconstructed at the original resolution. In most of the state-of-the-art papers on image restoration, the results are presented at the same size as train size or only slightly bigger. However, in our case, the scaling factor is between two to four times the training size.

**Figure 6:**
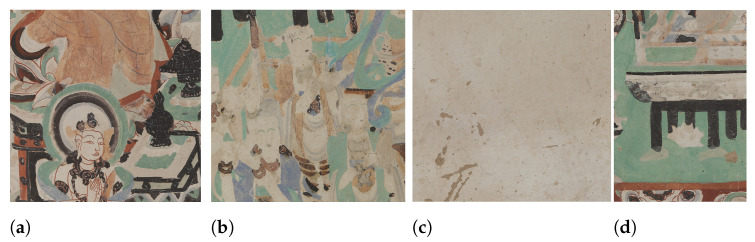
Ground-truth images selected for discussion. (**a**) First image (697 × 701 pixels) was chosen because it contains a face, that we consider a challenging case for inpainting. (**b**) Second image (681 × 674) is very color diverse. (**c**) Third image (828 × 800) is a homogenous colour, where it will be easy to check for color artifacts in the inpainted result. (**d**) Fourth image (582 × 841) is a scene with decorating motifs, where edges reconstruction can be inspected.

**Figure 7:**
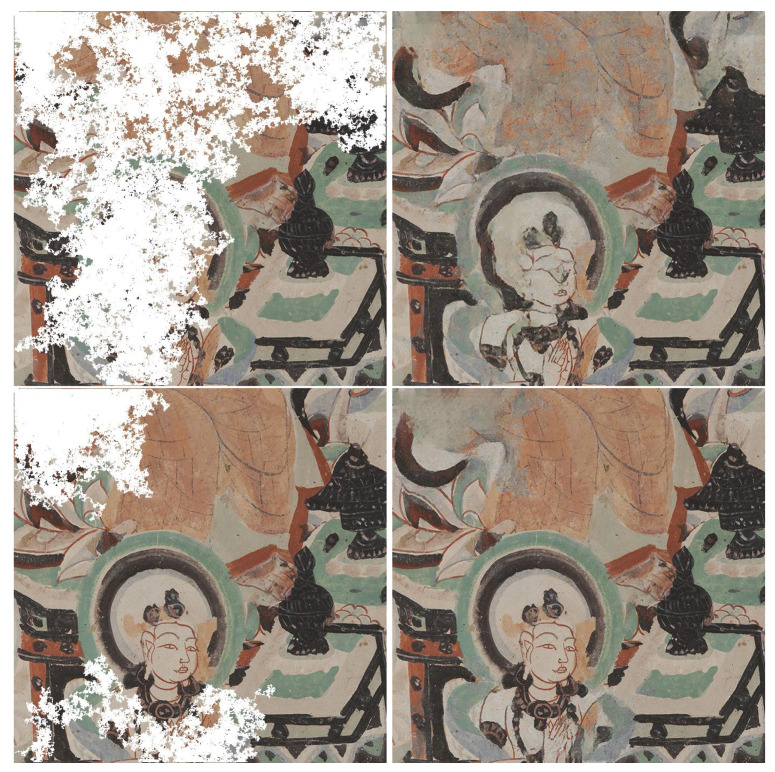
Inpainting results for random walk deterioration with different coverage of the same scene (ground-truth is Figure 6a). The inpainted images were generated at full resolution, however they are shrinked for display purposes here. Even though, in the first deteriorated example, the face of the character is mostly covered, the inpainted version manages to reconstruct some structural details with good accuracy, such as the aura, the lips and the left eye.

For visual inspection, we selected four images that are displayed in Figure 6, that we consider challenging cases for inpainting. Figure 7 shows two random walk degradations over a scene that contains a character (Figure 6a). The first deterioration hides most of the details of the face and decorations above the head of the character. Faces contain fine structural details that are challenging for an inpainting task. Our result is able to recover the details for the aura, the left eye, the lip, and the base of the nose. In the same way, the second deterioration occludes the palms and fingers that are recovered in the inpainted image. Nonetheless, a downside of this reconstruction is that the orange wings in the top center are not completely free from color artifacts.

**Figure 8:**
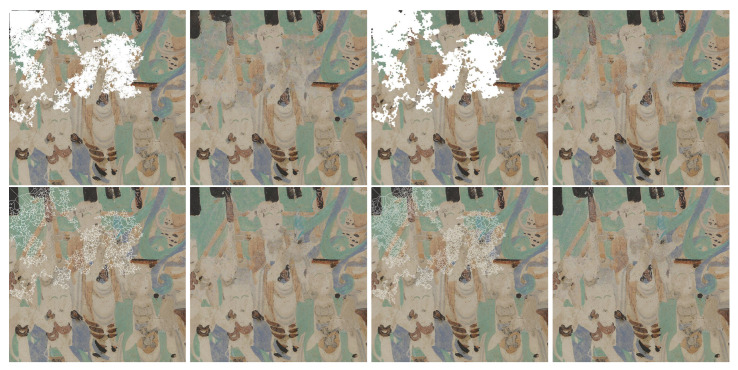
Pairs of inpainting results for morphological variations of random walk masks with different coverage of the same scene (ground-truth is Figure 6b). The colors are well preserved in the restored version. In the second pair (top third and fourth images), where dilated random walk deterioration is used, we can notice more blurriness.

**Figure 9:**
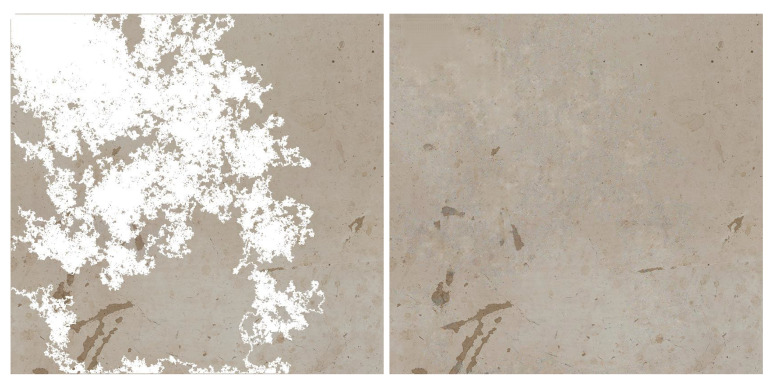
Inpainting result for Figure 6c. The restoration does not disrupt the color homogeneity with respect to the non-corrupted part of the image.

**Figure 10:**
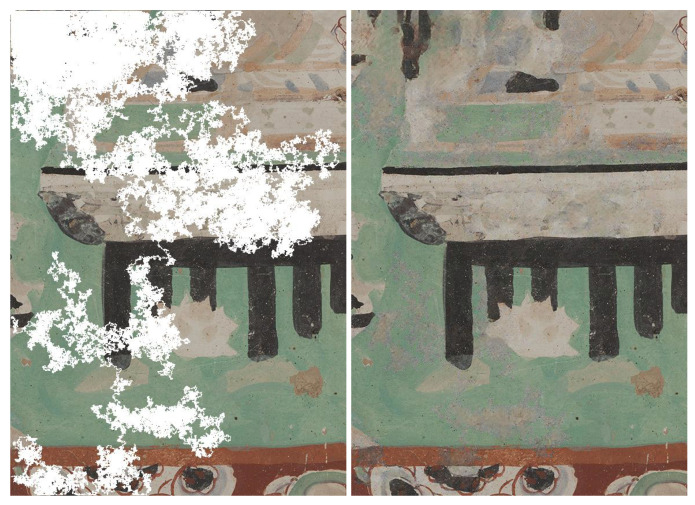
Inpainting result for Figure 6d. The beige structure in the middle of the image remains color coherent in the infilled image. Similarly, the two leftward black poles. However, the reconstruction is more blurry in the top left corner, which corresponds to a bigger and more contiguous lacuna.

On the other hand, Figure 8 and Figure 9 show an accurate restoration of the color content. The gap shown in Figure 8 cover distinct color tones from Figure 6b that are well retrieved in the restored result. Similarly, Figure 9 retouches Figure 6c, which lies as a patch of homogeneous color where chromatic inaccuracy would be easily detected. However, the infilling adds color of a hue similar to the original. The last example (Figure 6d) is a painting of a temple that is rich in structural details. The color coherence is maintained in the beige roof in the middle of the image, but it shows greenish artifacts in the orange stripe in the bottom of the image, as depicted in Figure 10.

**Figure 11:**
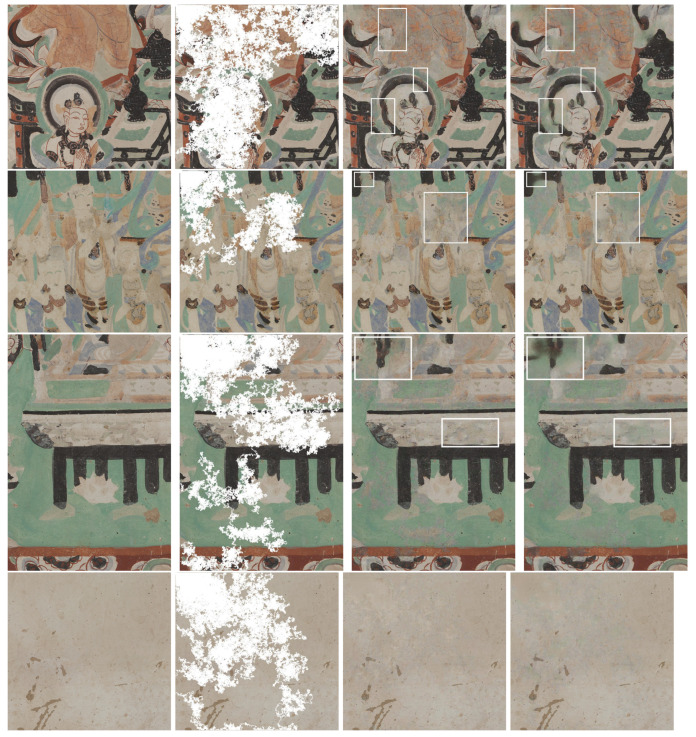
The columns represent in order, from left to right: original image, deteriorated image with RW mask, inpainted image with our approach, inpainted image with the approach of [17]. In the highlighted regions of interest, our approach outputs more color coherent and sharper results.

**Figure 12:**
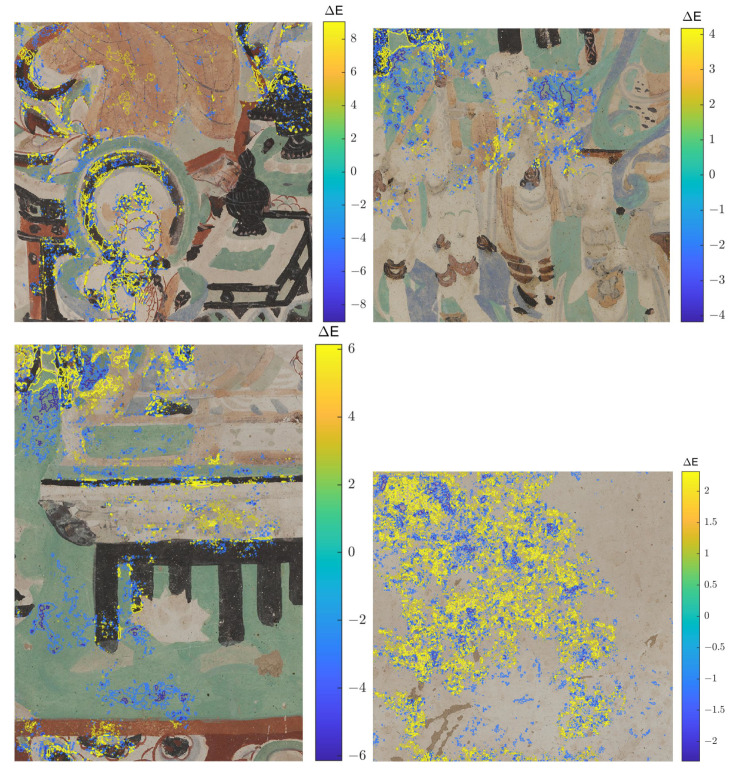
Contour plots showing the isolines of difference in ΔE error between images inpainted with the proposed method minus the method in [17]. For each method, ΔE was computed with the S-CIELAB distance metric, where the reference was the original, undamaged image. A positive value (yellow) for the contour plots show areas where our proposed method is more color coherent with the original, whereas negative values (blue) represent regions where the other algorithm performs better.

We have quantitatively evaluated the performance of our method with traditional image quality metrics (IQM), as well as CNN-enhanced IQMs. These metrics are computed starting from the RGB rendering of the output of our approach in comparison to that of [17] trained from scratch on our dataset. Out of the traditional IQMs, we present Structural Similarity Index (SSIM) [34] with a window size 11, Peak Signal-to-Noise Ratio (PSNR), colorimetric difference with the CIEDE2000 formula [35], as well as the spatial extension of the CIELAB colorimetric difference (S-CIELAB) [36]. The results in Table 1 are reported for each mask type. The results are better for the gap that occupies less space in the image. Accordingly, the metrics indicate that the highest quality of reconstruction is achieved on holes that simulate craquelure due to the minimal corruption they bring to the original image. Meanwhile, the lowest performance of the metrics corresponds to the dilated random walk masks, because of their increased coarseness of the pattern and a higher take-over of the image. Even though, numerically, our approach does not outperform [17] for the four traditional IQMs, the differences are not significantly apart. Actually, the differences between the compared methods for the color metrics quantified as CIEDE2000 and S-CIELAB are under the "just noticeable difference” ΔE unit. Meanwhile, PSNR and SSIM are known to be low estimators of the human perception and, in addition, they have very low sensitivity to color (as a matter of fact the input to SSIM is grayscale) and spatial effects (such as blur or constrast). By visually assessing selected images that are inpainted by the two approaches (see Figure 11), we can see that the restoration that is generated with our approach appears to be more color coherent and sharper in localized areas with respect to the results obtained with [17]. The pictures in the third and fourth rows, last column exemplify color hue artifacts where beige color gets replaced with green tones, which are not produced by the proposed method. We would like to suggest that for the color metrics, a more meaningful representation than numeric values that are aggregated over all images and pixels, is actually a distance map, where the chromatic differences are correlated with their spatial context. As a matter of fact, in order to enforce the above-mentioned visual hints perceived in Figure 11, we computed Spatial-CIELAB [36] distance maps between the original, our proposed method and the method in [17]. S-CIELAB computes the color difference between images, after applying a spatial processing step as a simulation of the human vision bandpass filtering. This way, instead of considering pixel-only variations, it measures the color distance on more semantically meaningful spatial patterns. Figure 12 depicts the difference in ΔE error between our approach and [17] based on S-CIELAB computed for the selected images in Figure 6. We visualize these differences as contour plots, where positive values (yellow) highlight areas in the images where our method performs better colorimetrically while negative values (blue) show where the other method works better. The contour plots display isolines of ΔE differences at specific levels defined as −2σ, −σ, σ, 2σ, where σ is the standard deviation of each case’s distribution. These comparisons are consistent with the visual observation of images shown in Figure 11 and they prove that both models have selective performance. For more visual assessments of these two approaches, please refer to the Supplementary Material to this article.

Recent works in the field of image quality evaluation have demonstrated the effectivenes of CNN-based IQMs [37,38,39]. In other words, measuring the similarity in the feature space extracted by convolutional neural networks is in more agreement with the human subjective scores than by directly comparing end-level images. In [37], Amirshahi et al. compute the similarity of feature maps extracted from CNNs (AlexNet and VGG19 networks pretrained on ImageNet dataset). They compute the self-similarity of feature maps at each convolutional layer and at multiple levels of spatial resolution inspired by the Pyramid Histogram of Oriented Gradients (PHOG) approach. The final aggregate metric is given by the geometric mean of the single metrics determined for each convolutional layer. They test their method on four image quality datasets of natural images with various distortions and show that the CNN-based self-similarity measure has more correlation with human subjective scores than PSNR, SSIM, and S-CIELAB metrics. This work is extended in [38], where CNN feature maps are compared with traditional IQMs (such as PSNR and SSIM). Similar to [37], the finding is that the CNN-enhancement of IQMs (computed on CNN feature maps) is significantly more correlated with human perception than their traditional variant (applied on end-level image). In addition, the performance of all the proposed CNN metrics is not affected by changing the CNN network [37,38].

**Figure 13:**
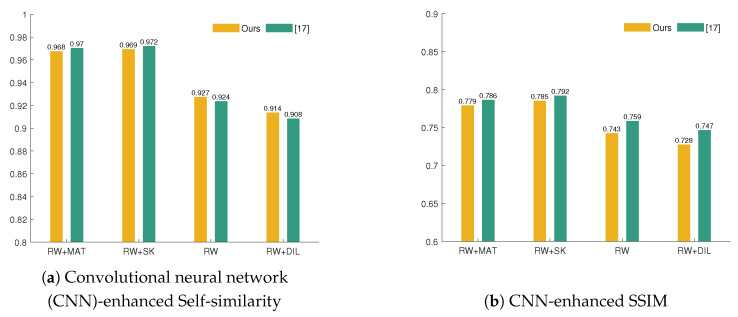

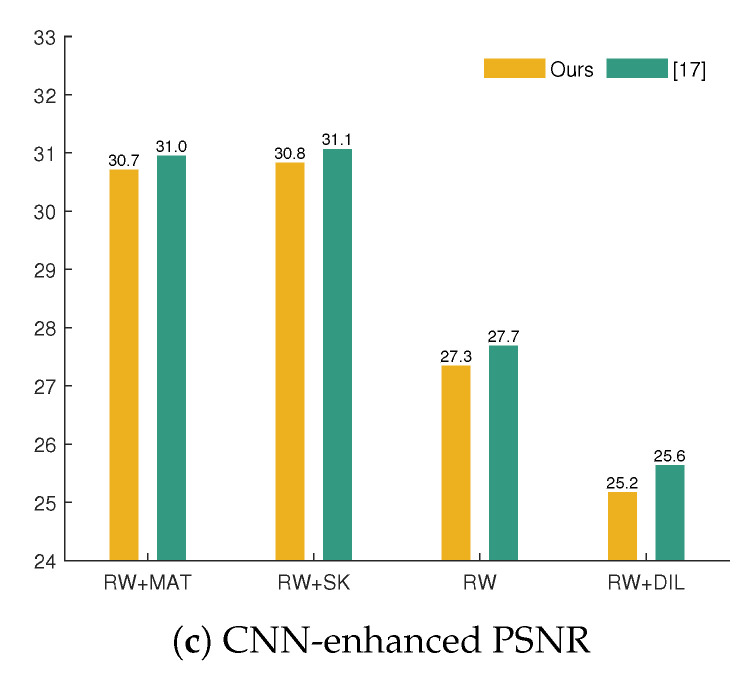
Bar plots showing the overall performance of three CNN-based mage quality metrics (IQMs) for groups of damage. These metrics measure the similarity of the feature maps given by the activations of AlexNet (pretrained on ImageNet) for the ground-truth test images and images inpainted by our approach and [17]. The overall metrics are aggregated as the geometric mean of the result at each convolutional layer.

**Figure 14:**
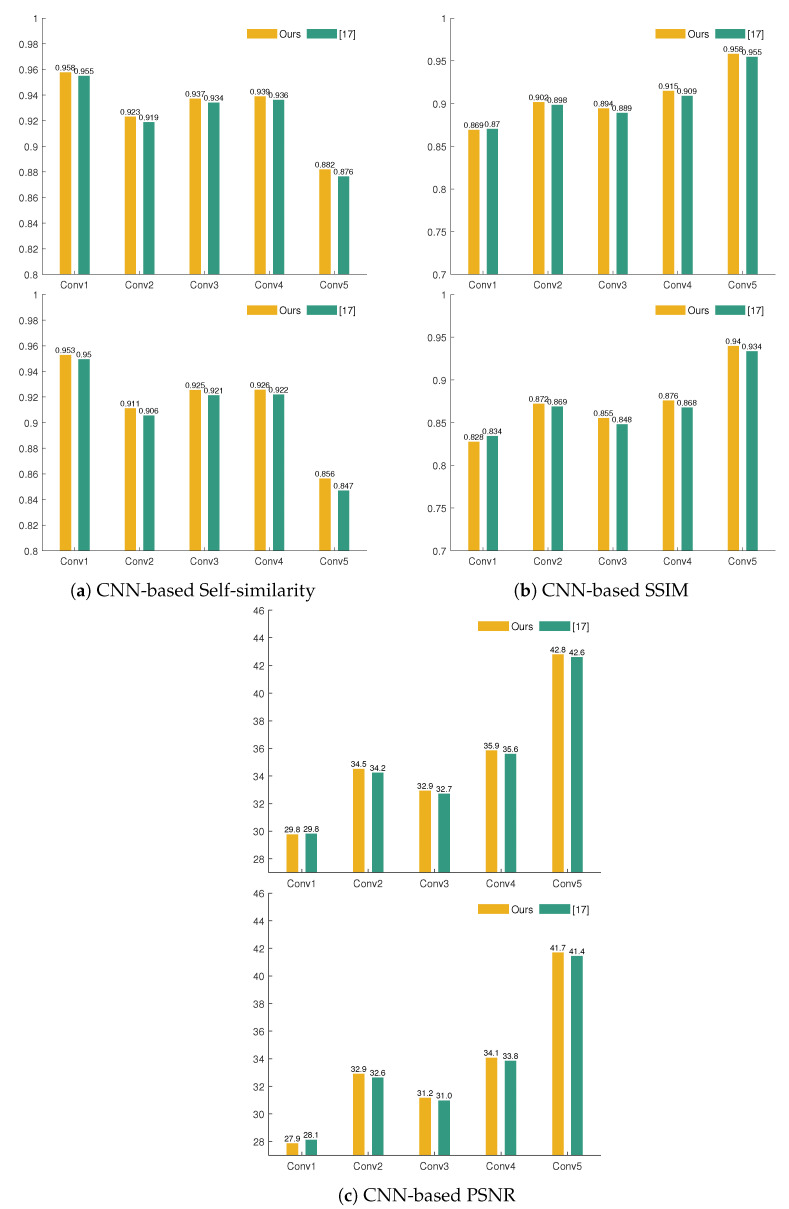
Bar plots showing the performance of three CNN-based IQMs for each convolutional layer. These metrics measure the similarity of the feature maps given by the activations of AlexNet (pretrained on ImageNet) for the ground-truth test images and images inpainted by our approach and [17]. Top for each pair: the results for RW damage. Bottom for each pair: results for RW+DIL damage.

Hence, drawing from the insights of [37,38], we validate our method with three metrics that measure the similarity between features maps that were extracted with the pretrained AlexNet for the ground-truth and inpainted images; Self-Similarity based on Pyramid Histogram of Oriented Gradients [37], CNN-enhanced PSNR, and CNN-enhanced SSIM [38]. Figure 13 displays the global performance of these metrics (pooled from the intermediate results at each convolutional layer). Based on CNN-enhanced PHOG self-similarity (Figure 13a), our method slightly outperforms [17] for bigger areas of damage (RW, RW+DIL). However, the overall values of CNN-enhanced PSNR and SSIM for [17] are better than ours. Nonetheless, we continue the analysis by exploring the internal representations of these metrics and study their performance for each convolutional layer. In this sense, one important finding of [37,38] is the proportionality between the order of the convolutional layer and the correlation with subjective scores. Thus, the first convolutional layer, because of embedding lower-level features, is farther away from human perception than the fifth convolutional layer that embeds higher-level features. Figure 14b,c show that the CNN-enhanced SSIM and PSNR for convolutional layers 2–5 are better for our method in comparison with [17], even for larger missing content (RW, RW + DIL). We could further infer that what draws back the pooled overall results for our method (Figure 13c and Figure 14b) is explained by the poorer performance of the first convolutional layer only. This suggests that our method preserves more of the mid-level and higher-level features in restoring the original images. It is noteworthy to relate these findings with a study on color responsivity of AlexNet [40], where it is claimed that all layers have color responsivity in a trend that decreases from first convolutional layer to the fourth one and then rises back in the fifth layer. Accordingly, the highest color responsivity is given by the first and last layer. Nonetheless, the first layer deals with a basic encoding of the color signal, while the last layer responds to more complex color encodings. Therefore, we could extrapolate that, based on the higher performance of all CNN-based metrics for the last convolutional layer, our method handles better complex entanglements between spatial and chromatic signals.

Regarding the performance of all image quality metrics, it is noteworthy to mention that compression artifacts (the dataset is available as JPEG images) might represent an error factor in the pipeline. Similarly, the conversion back and forth from RGB to L*a*b* color space might introduce some numerical loss in the quantitative analysis. This transformation takes place more times than in the method of [17] due to the way our pipeline is designed.

It is not trivial to draw a straightforward and fair comparison with other relevant state-of-the-art models, such as [3,23,25], where the exact dataset, train-test split, and model implementation are not available to perform a one-to-one mapping to our approach. So much so, we provide a rough comparison in Table 2, where we outline key aspects of every method, including the minima and maxima of PSNR and SSIM metrics where applicable. Letting aside the variations in data, mask type, and precise extent of missing pixels reported for each method, we can compare these results by identifying their common purpose: solving the digital inpainting task for Buddhist mural paintings with deep learning approaches. With these considerations in mind, Wang et al., in [23], simulate four levels of damage as is our case, even though the ratio of corrupted pixels to non-corruped pixels per level remains unknown. Compared to the IQMs reported by [23], our work achieves higher PSNR extremes, while lower SSIM extremes. It seems that the user input in the interactive Deep Image Prior method of [25] leverages the SSIM to a value of 0.78 for random-walk type of damage, which is higher than our results for the RW category (0.74). Nonetheless, [25] pool their results from only 10 images. At the same time, Yu et al. [22] include no quantitative result, so we are not able to draw any numeric comparison with their method. While our method does not outperform the SSIM and PSNR reported by [17,23], it is noteworthy to emphasize that the proposed approach works selectively better than [17] for certain areas in the image, as presented in the color difference maps of Figure 12. This selective performance is reinforced by the CNN-based metrics that prove our method handles better the processing of higher level combinations of spatial and chromatic features.

It should be stated that our method is not restricted to only wall paintings and it can be applied to more types of paintings, as long as there is a sufficient dataset of digital color images to train the model. As far as the simulation of damage is concerned, our approach can be improved by considering more shapes and sizes for the loss patterns other than the already explored morphological variations of random walks. Moreover, there are several configurations of that might limit the performance of the results and that could potentially be changed and tuned in future work, such as: the input image size for the training process that could be increased to achieve higher resolution, the number of quantization levels for the prior color palette fed to the network, and the use of image formats other than JPEG to mitigate the effect of the compression artifacts.

## 5. Conclusions

The virtual inpainting of artworks is an attractive application for art restorers as well as for the general public. Deep learning techniques and, in particular, generative adversarial networks, open new horizons for increasing accurate and sensible solutions in this application.

This paper evaluated an inpainting algorithm for a set of digitizations of the Dunhuang murals. By jointly learning edge and color content, the proposed algorithm is able to produce results, where these two features are coherent with the original. Moreover, it accounts for four types of deterioration patterns by employing various irregular mask that essentially follow a random walk trajectory. The limitations to our model are partially due to the discrepancy between the (lower) train and (higher) test resolution, partially due to JPEG compression artifacts and not entirely lossless conversion between RGB and CIELAB color spaces. Even so, there seems to be a miscoordination between the visual and quantitative appreciation of our results as a consequence of the imperfect integration of human perception factors in the existing image quality metrics.

## Figures and Tables

**Table 1:** Peak Signal-to-Noise Ratio (PSNR), Structural Similarity Index (SSIM), CIEDE2000, and S-CIELAB metrics between the ground-truth test images and the inpainted results, presented for each mask type. For PSNR & SSIM, higher values mean better closer resemblance to the ground-truth. For CIEDE2000 and S-CIELAB, higher values correlate with a higher colorimetric distance with respect to ground-truth.

Mask Type	Missing Pixels (%)	PSNR		SSIM		CIEDE2000		S-CIELAB	
		Ours	[17]	Ours	[17]	Ours	[17]	Ours	[17]
RW	28.42	26.57	26.86	0.74	0.76	4.09	4.05	3.75	3.71
RW + SK	6.93	29.89	30.12	0.81	0.82	3.19	3.10	2.30	2.19
RW + MAT	7.60	29.81	30.04	0.81	0.81	3.21	3.12	2.38	2.26
RW + DIL	31.20	24.75	25.18	0.72	0.73	4.44	4.34	4.24	4.13

**Table 2:** Rough comparison between our method and other relevant related works solving the restoration of wall paintings.

Approach	Dataset	Mask Type	Damage Levels	Nr. Images Evaluated Quantitatively	Quantitative Results			
					PSNR		SSIM	
					Lowest	Highest	Lowest	Highest
Ours	Dunhuang	RW, RW + DIL, RW + MAT, RW + SK	4	200	24.75	29.89	0.72	0.81
Yu et al. [22]	Dunhuang	Dusk-like (∼RW), Jelly-like (∼RW+DIL)	2	-	-			
Weber et al. [25]	Dunhuang	RW	1	10	-		0.78	
Wang et al. [23]	Thanka	Irregular lines and elliptical shapes	4	1391	21.23	33.12	0.74	0.98

## Data Availability

The images used for evaluating our method are available as the Supplementary Material.

## Supplementary Materials

The following are available online at https://www.mdpi.com/1424-8220/21/6/2091/s1. The damaged test images used for evaluation and the corresponding inpainted images obtained with our model.

## Abbreviations

The following abbreviations are used in this manuscript:

S-CIELAB	Spatial extension of the CIELAB colorimetric difference
JPEG	Joint Photographic Experts Group
PHOG	Pyramid Histogram of Oriented Gradients
PSNR	Peak Signal-to-Noise Ratio
SSIM	Structural Similarity Index Measure
CNN	Convolutional Neural Network
DIL	Dilation
DIP	Deep Image Prior
GAN	Generative Adversarial Network
IQM	Image Quality Metric
MAT	Medial Axis Transform
CH	Cultural Heritage
RW	Random Walk
SK	Skeletonization
