# Supplementary figures and images for: Colour-Balanced Edge-Guided Digital Inpainting: Applications on Artworks (part 2 of 2)
Source: Sensors (Basel). 2021 Mar 17;21(6):2091. doi: 10.3390/s21062091 (PMC8002538; doi:10.3390/s21062091)

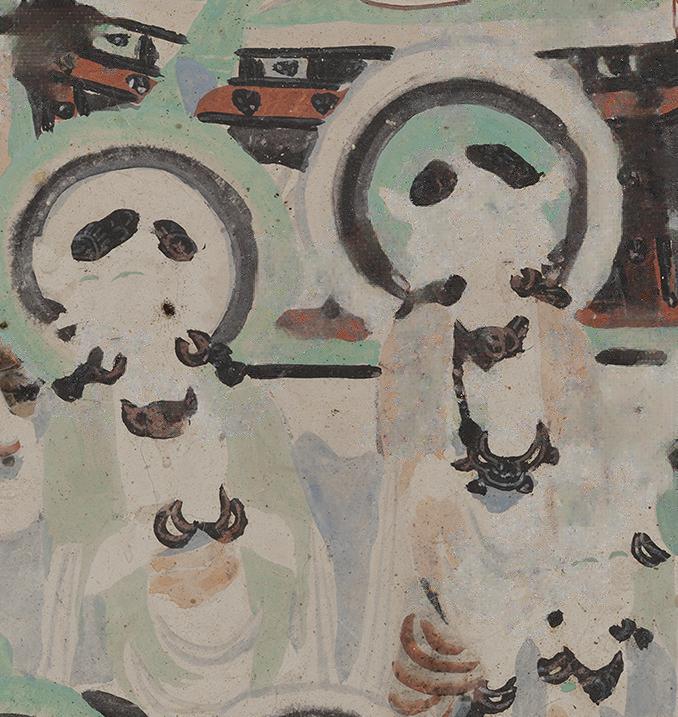

Supplement: Supplementary file 1 [file sensors-21-02091-s001.zip › smartsensors_supplementary_data/nazerietal-model-results/146_masked_972252_0.jpg]

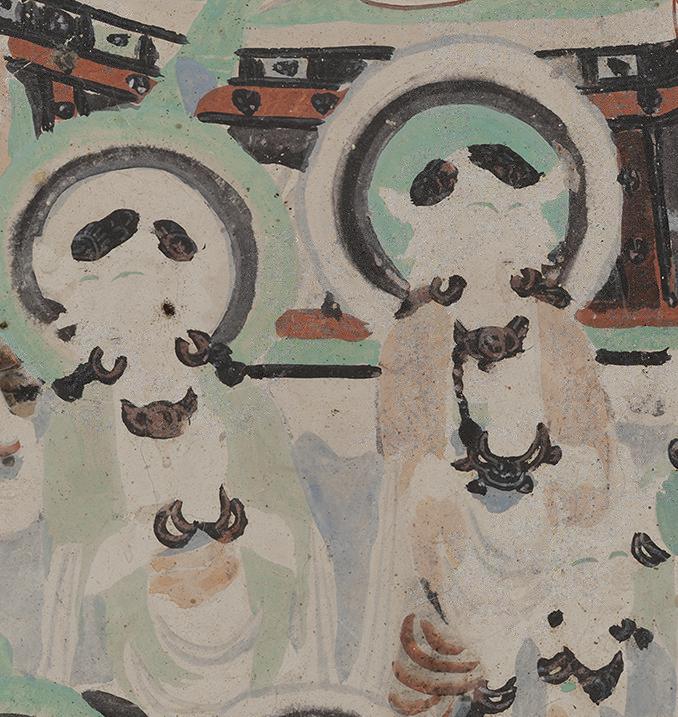

Supplement: Supplementary file 1 [file sensors-21-02091-s001.zip › smartsensors_supplementary_data/nazerietal-model-results/146_masked_972252_1.jpg]

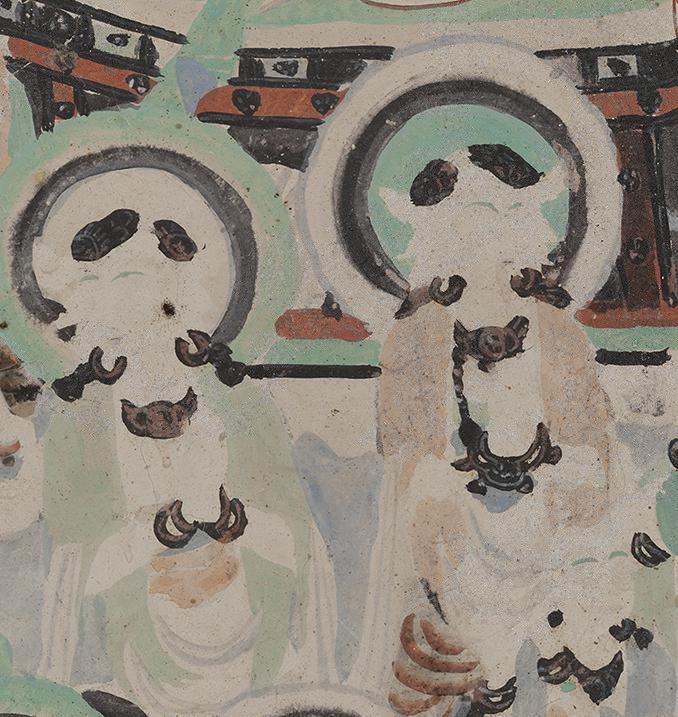

Supplement: Supplementary file 1 [file sensors-21-02091-s001.zip › smartsensors_supplementary_data/nazerietal-model-results/146_masked_972252_2.jpg]

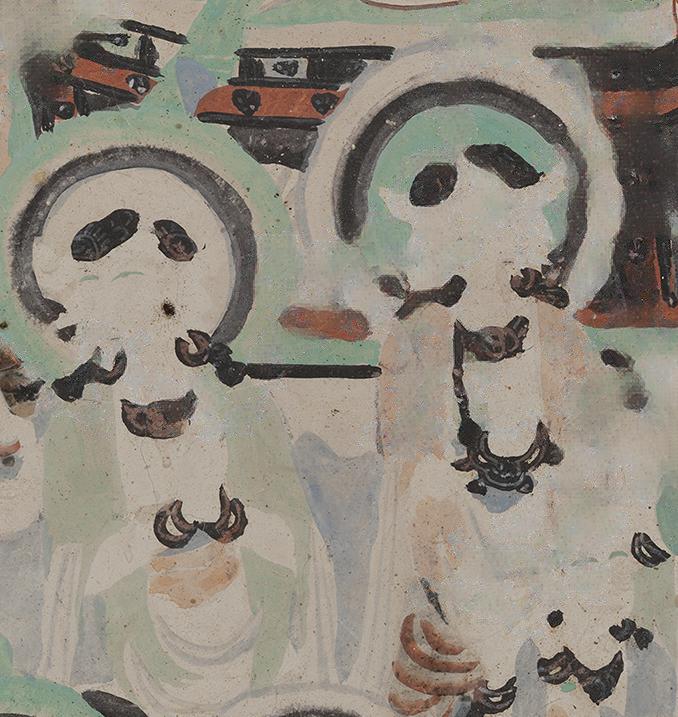

Supplement: Supplementary file 1 [file sensors-21-02091-s001.zip › smartsensors_supplementary_data/nazerietal-model-results/146_masked_972252_3.jpg]

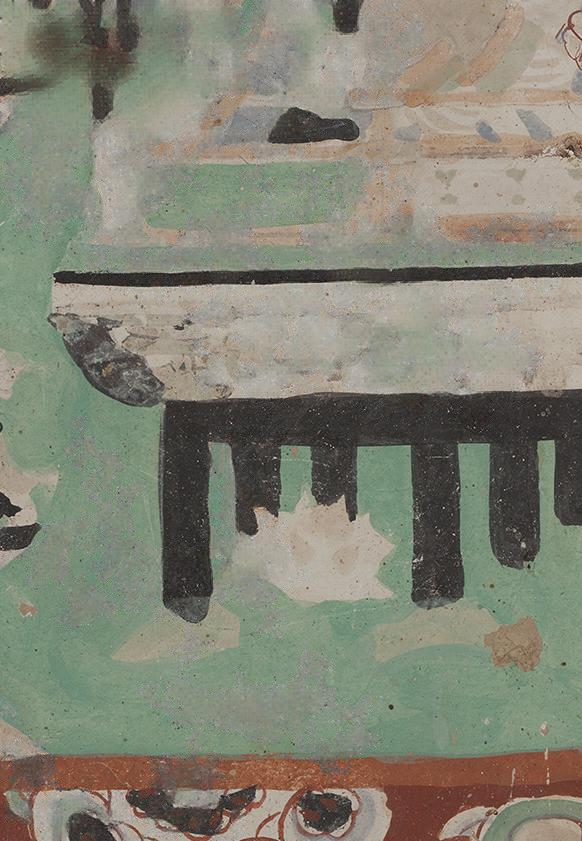

Supplement: Supplementary file 1 [file sensors-21-02091-s001.zip › smartsensors_supplementary_data/nazerietal-model-results/162_masked_1713117_0.jpg]

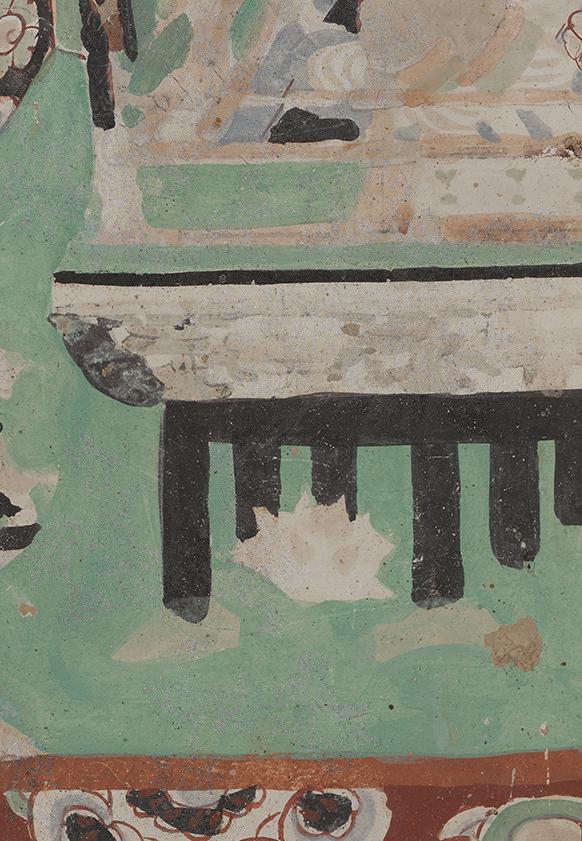

Supplement: Supplementary file 1 [file sensors-21-02091-s001.zip › smartsensors_supplementary_data/nazerietal-model-results/162_masked_1713117_1.jpg]

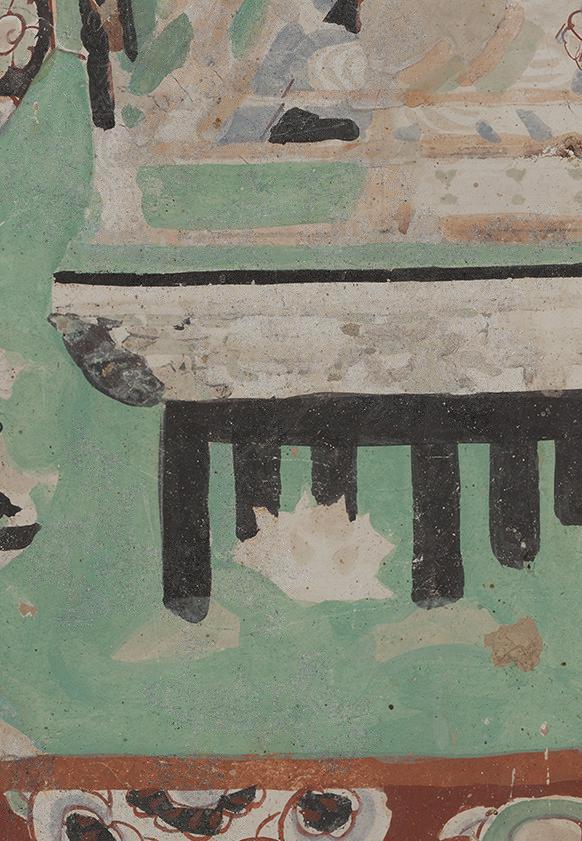

Supplement: Supplementary file 1 [file sensors-21-02091-s001.zip › smartsensors_supplementary_data/nazerietal-model-results/162_masked_1713117_2.jpg]

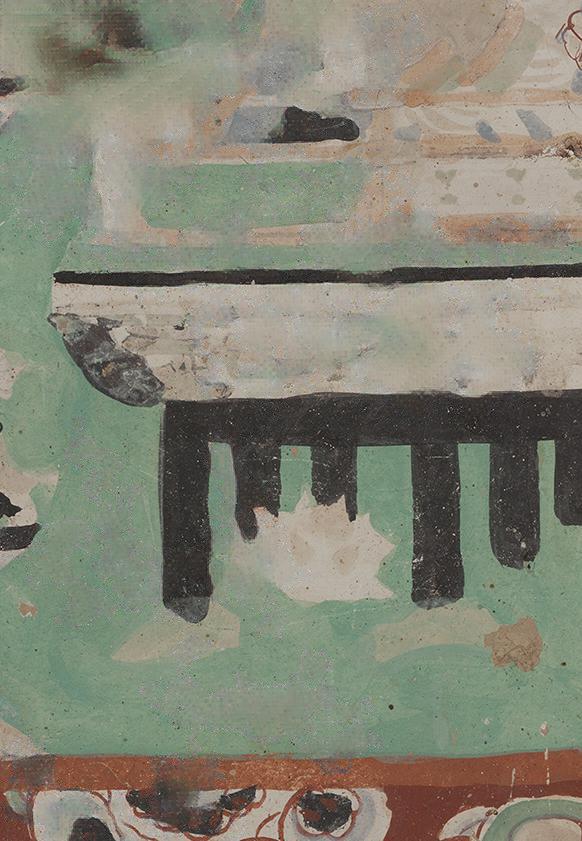

Supplement: Supplementary file 1 [file sensors-21-02091-s001.zip › smartsensors_supplementary_data/nazerietal-model-results/162_masked_1713117_3.jpg]

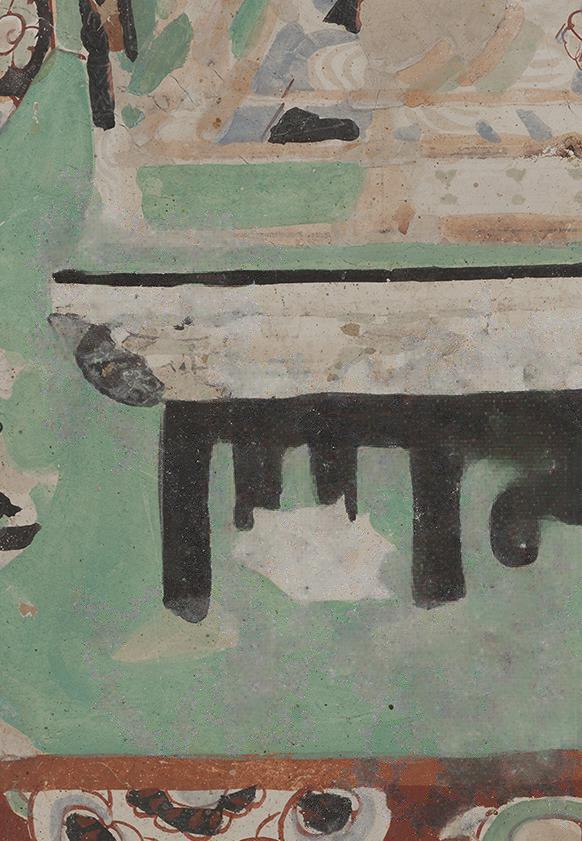

Supplement: Supplementary file 1 [file sensors-21-02091-s001.zip › smartsensors_supplementary_data/nazerietal-model-results/162_masked_978924_0.jpg]

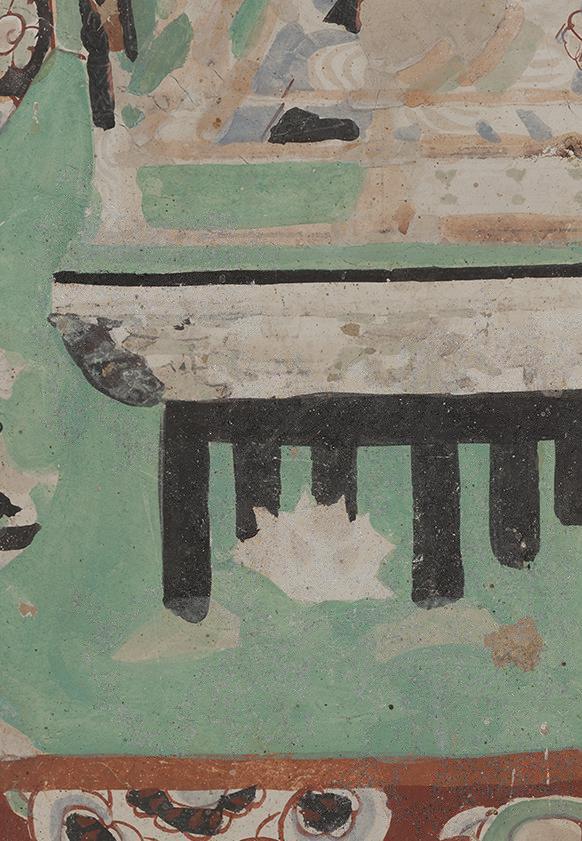

Supplement: Supplementary file 1 [file sensors-21-02091-s001.zip › smartsensors_supplementary_data/nazerietal-model-results/162_masked_978924_1.jpg]

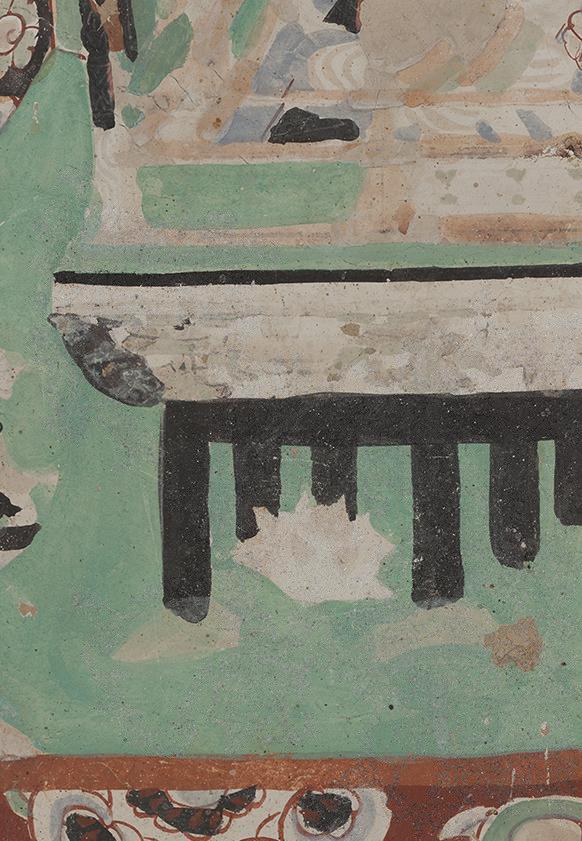

Supplement: Supplementary file 1 [file sensors-21-02091-s001.zip › smartsensors_supplementary_data/nazerietal-model-results/162_masked_978924_2.jpg]

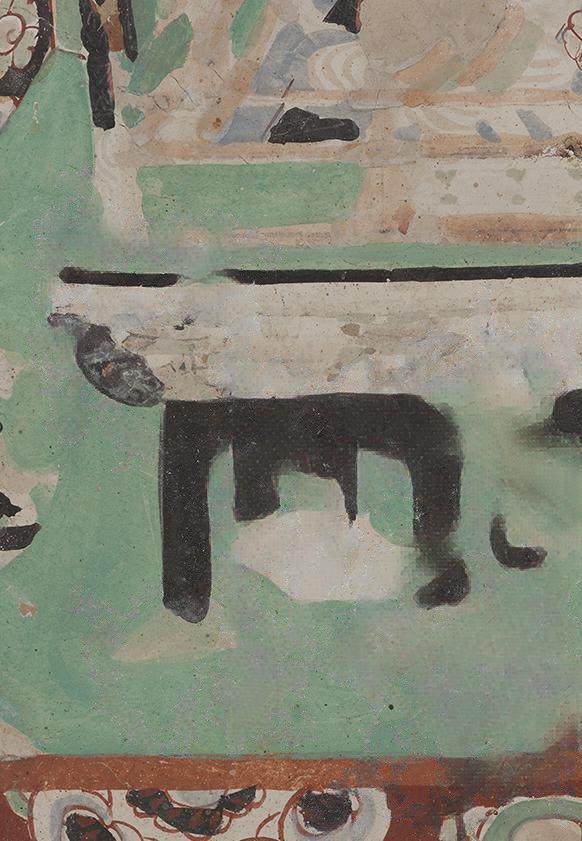

Supplement: Supplementary file 1 [file sensors-21-02091-s001.zip › smartsensors_supplementary_data/nazerietal-model-results/162_masked_978924_3.jpg]

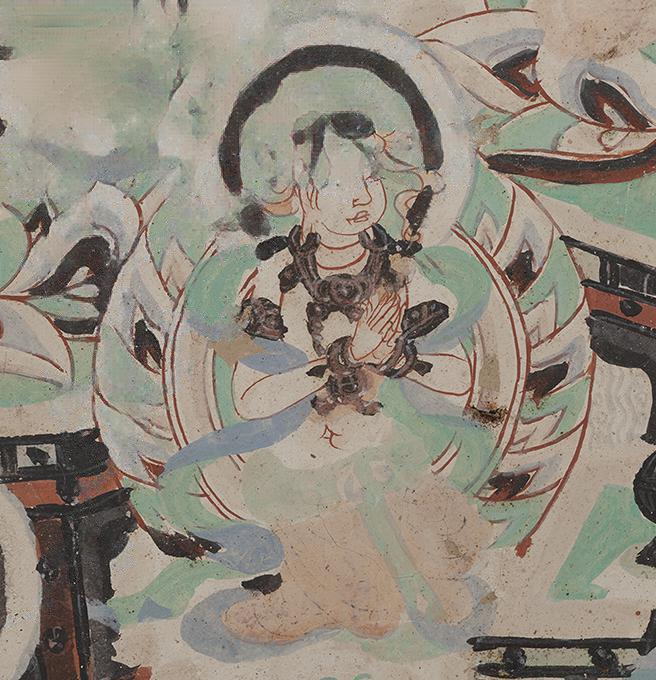

Supplement: Supplementary file 1 [file sensors-21-02091-s001.zip › smartsensors_supplementary_data/nazerietal-model-results/178_masked_1561280_0.jpg]

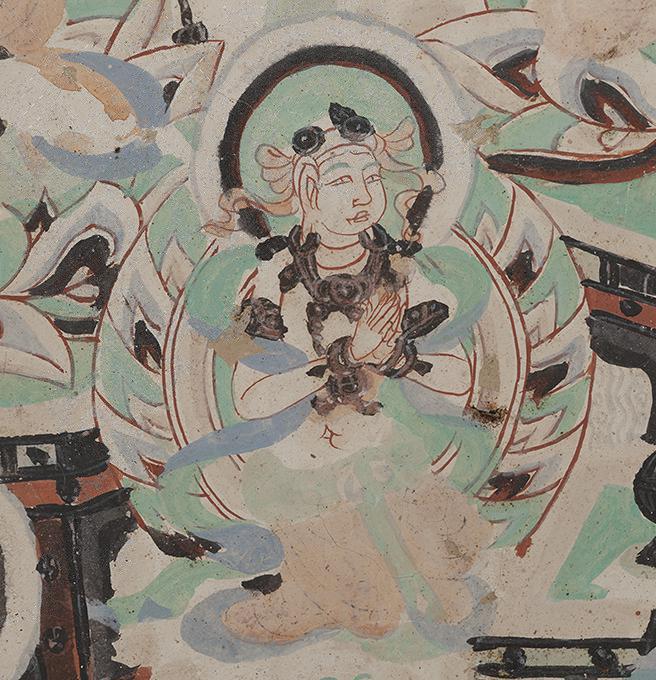

Supplement: Supplementary file 1 [file sensors-21-02091-s001.zip › smartsensors_supplementary_data/nazerietal-model-results/178_masked_1561280_1.jpg]

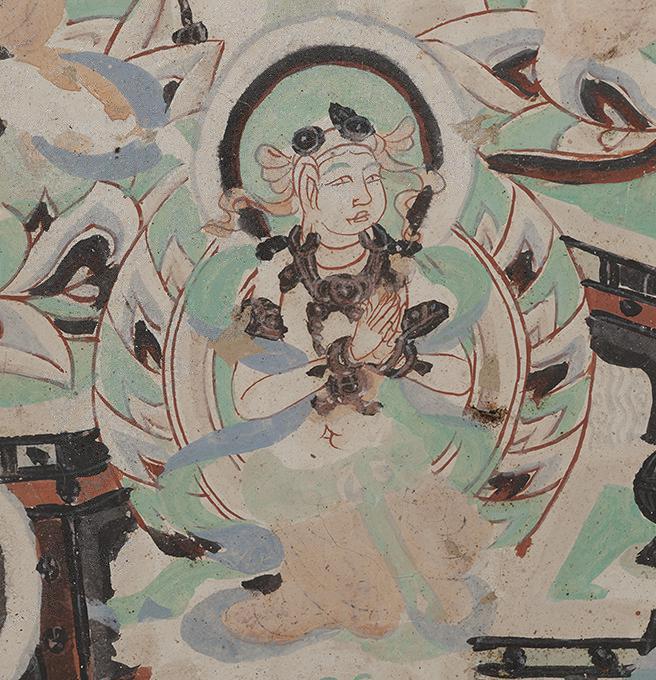

Supplement: Supplementary file 1 [file sensors-21-02091-s001.zip › smartsensors_supplementary_data/nazerietal-model-results/178_masked_1561280_2.jpg]

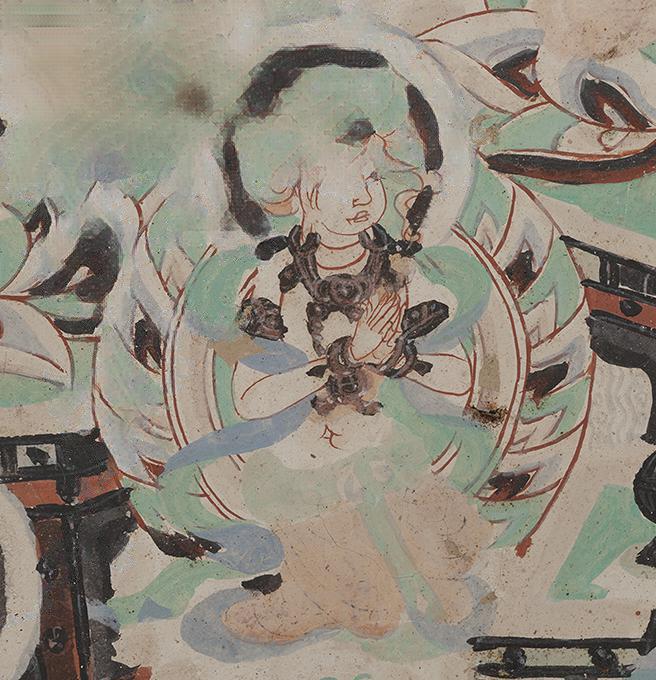

Supplement: Supplementary file 1 [file sensors-21-02091-s001.zip › smartsensors_supplementary_data/nazerietal-model-results/178_masked_1561280_3.jpg]

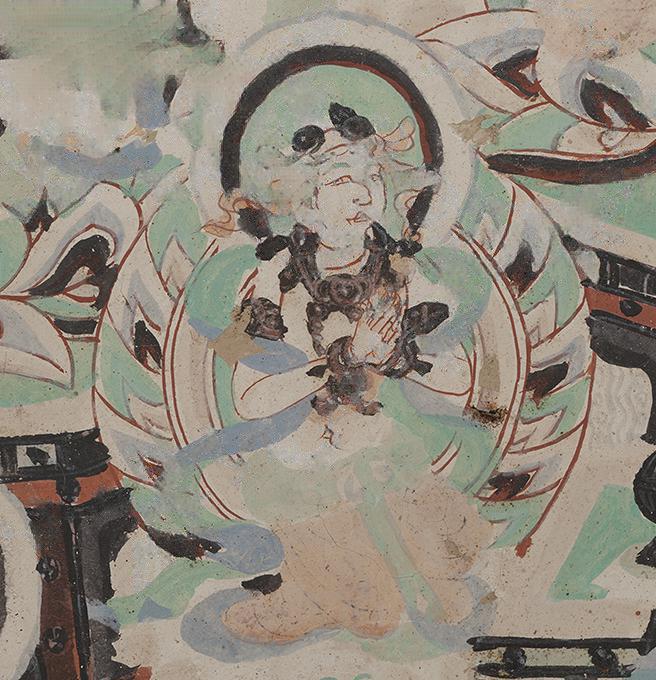

Supplement: Supplementary file 1 [file sensors-21-02091-s001.zip › smartsensors_supplementary_data/nazerietal-model-results/178_masked_892160_0.jpg]

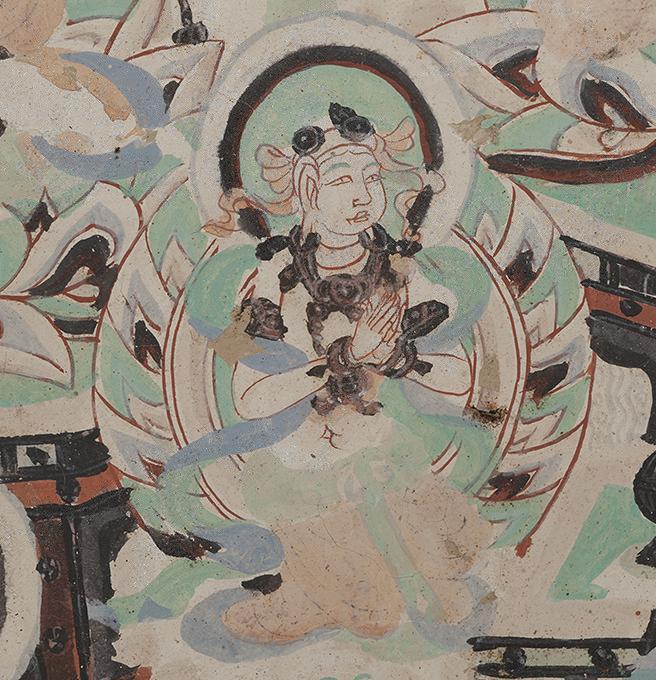

Supplement: Supplementary file 1 [file sensors-21-02091-s001.zip › smartsensors_supplementary_data/nazerietal-model-results/178_masked_892160_1.jpg]

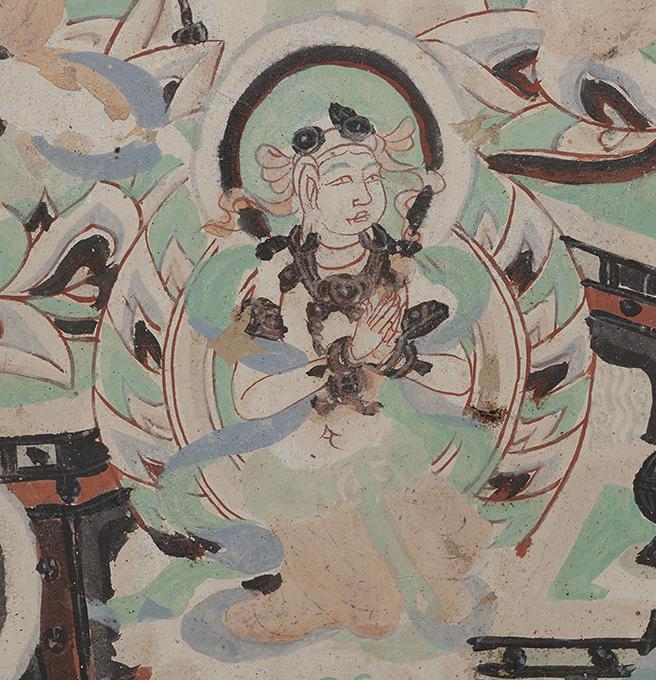

Supplement: Supplementary file 1 [file sensors-21-02091-s001.zip › smartsensors_supplementary_data/nazerietal-model-results/178_masked_892160_2.jpg]

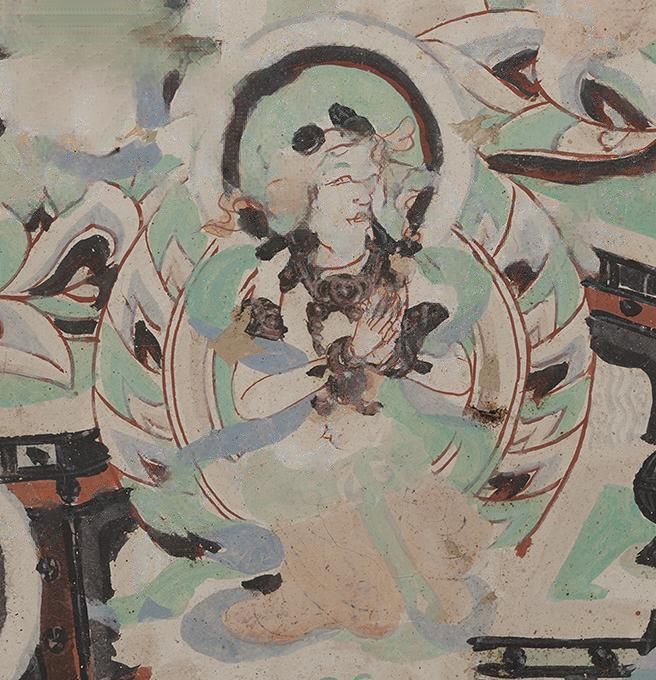

Supplement: Supplementary file 1 [file sensors-21-02091-s001.zip › smartsensors_supplementary_data/nazerietal-model-results/178_masked_892160_3.jpg]

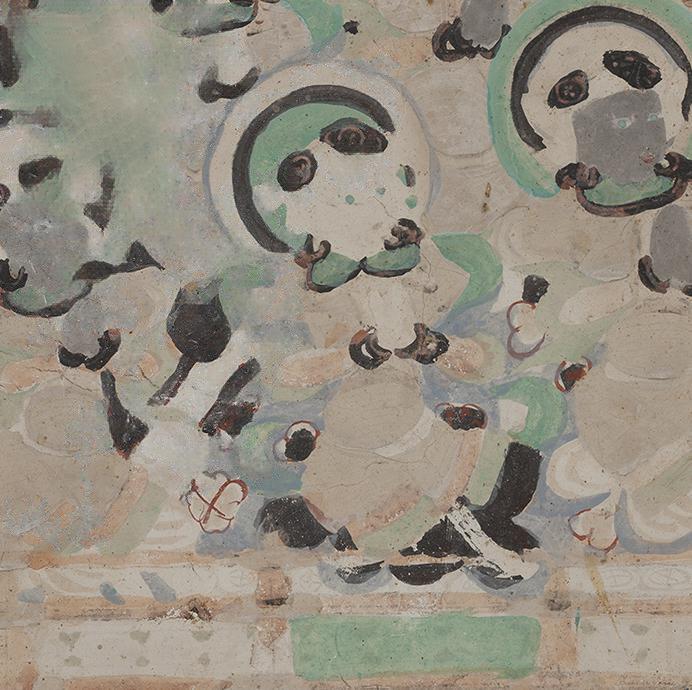

Supplement: Supplementary file 1 [file sensors-21-02091-s001.zip › smartsensors_supplementary_data/nazerietal-model-results/204_masked_1671180_0.jpg]

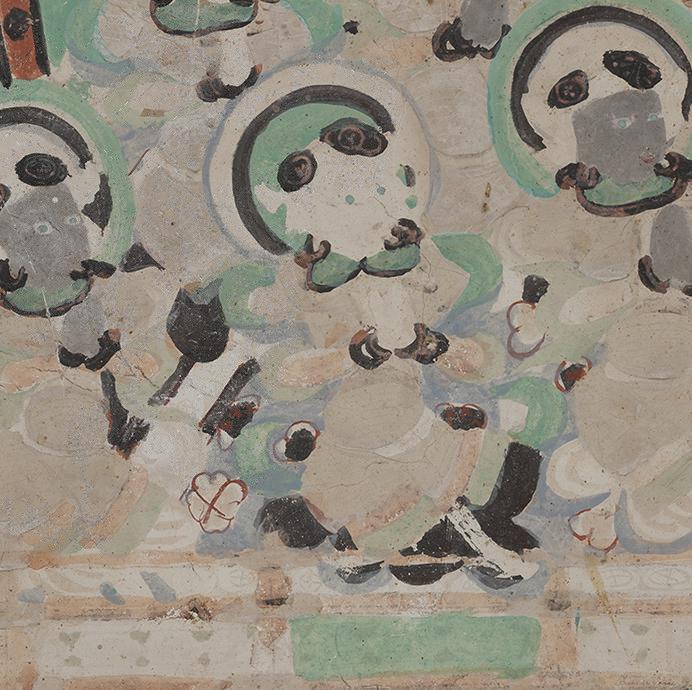

Supplement: Supplementary file 1 [file sensors-21-02091-s001.zip › smartsensors_supplementary_data/nazerietal-model-results/204_masked_1671180_1.jpg]

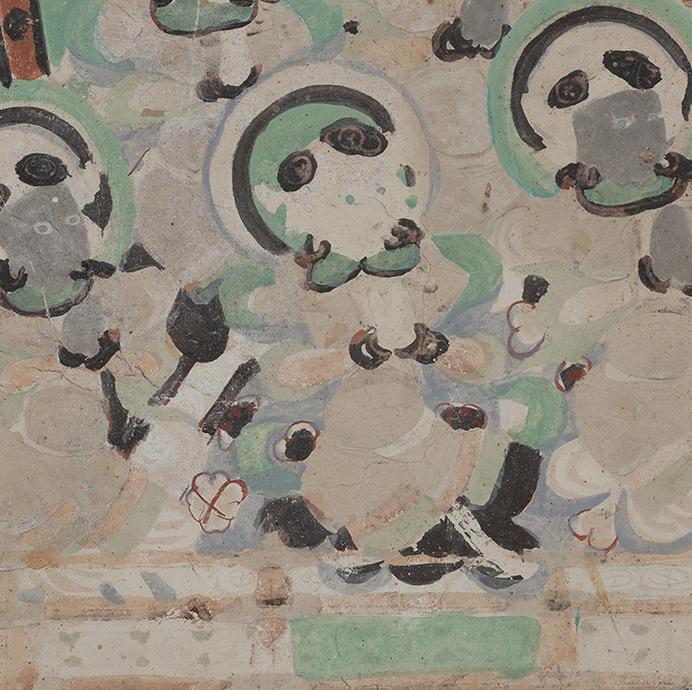

Supplement: Supplementary file 1 [file sensors-21-02091-s001.zip › smartsensors_supplementary_data/nazerietal-model-results/204_masked_1671180_2.jpg]

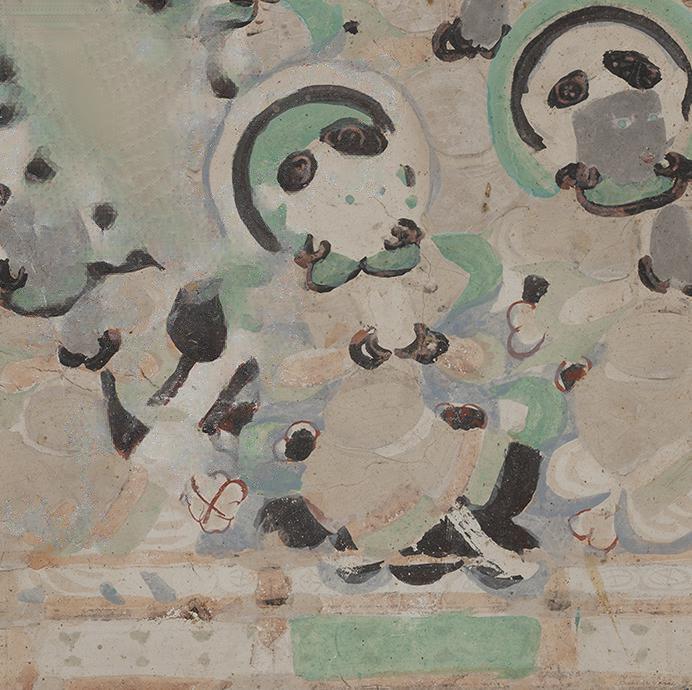

Supplement: Supplementary file 1 [file sensors-21-02091-s001.zip › smartsensors_supplementary_data/nazerietal-model-results/204_masked_1671180_3.jpg]

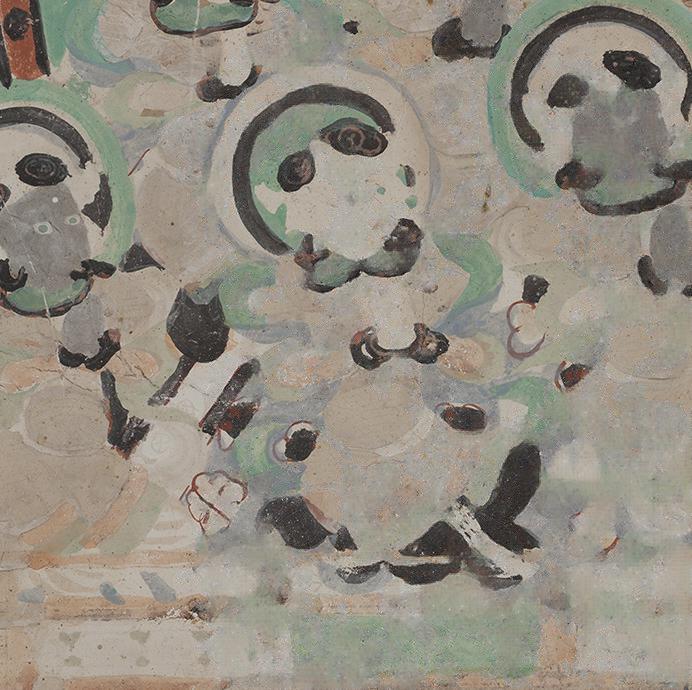

Supplement: Supplementary file 1 [file sensors-21-02091-s001.zip › smartsensors_supplementary_data/nazerietal-model-results/204_masked_954960_0.jpg]

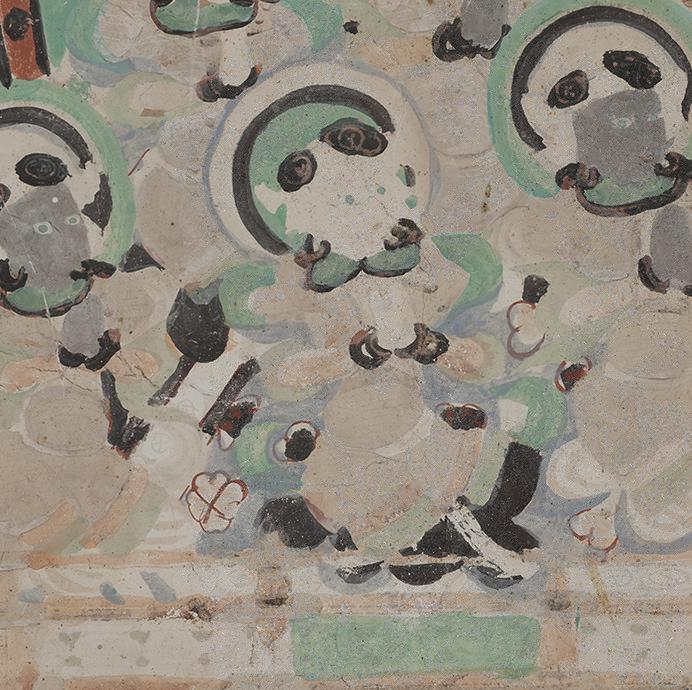

Supplement: Supplementary file 1 [file sensors-21-02091-s001.zip › smartsensors_supplementary_data/nazerietal-model-results/204_masked_954960_1.jpg]

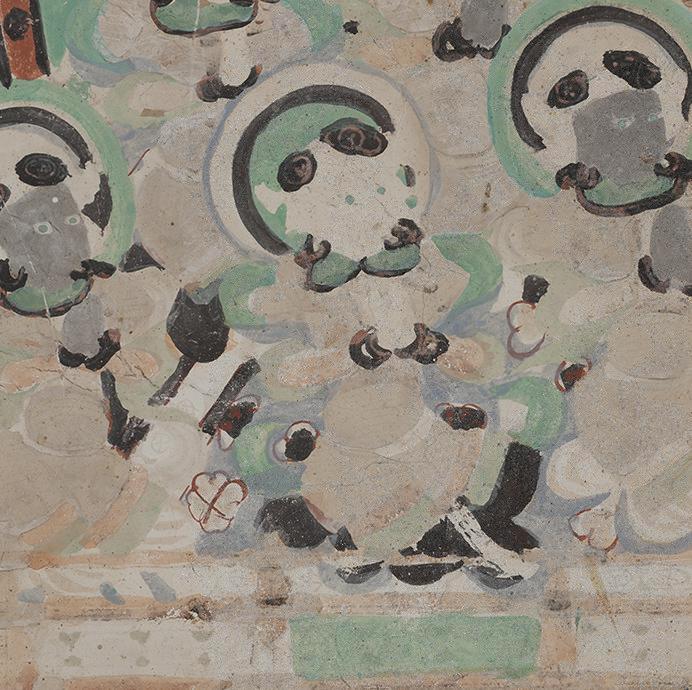

Supplement: Supplementary file 1 [file sensors-21-02091-s001.zip › smartsensors_supplementary_data/nazerietal-model-results/204_masked_954960_2.jpg]

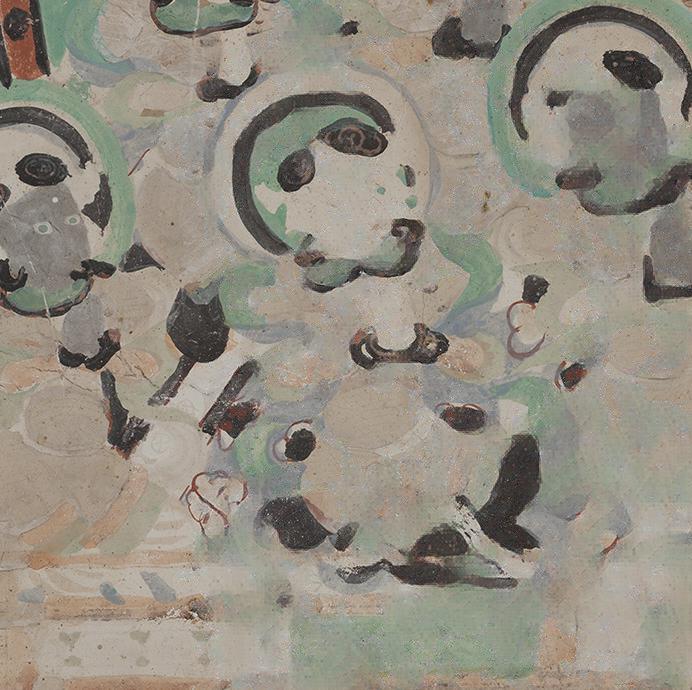

Supplement: Supplementary file 1 [file sensors-21-02091-s001.zip › smartsensors_supplementary_data/nazerietal-model-results/204_masked_954960_3.jpg]

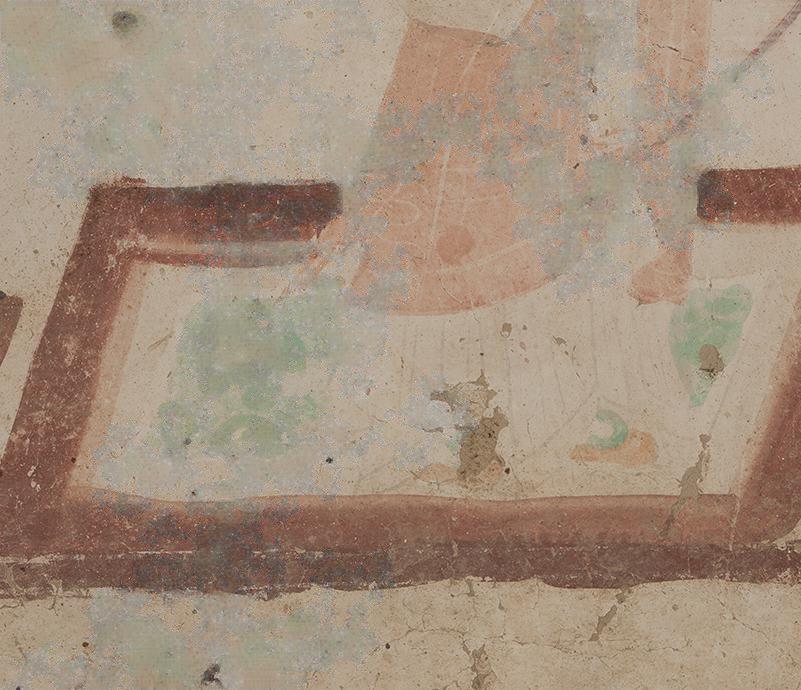

Supplement: Supplementary file 1 [file sensors-21-02091-s001.zip › smartsensors_supplementary_data/nazerietal-model-results/217_masked_1105380_0.jpg]

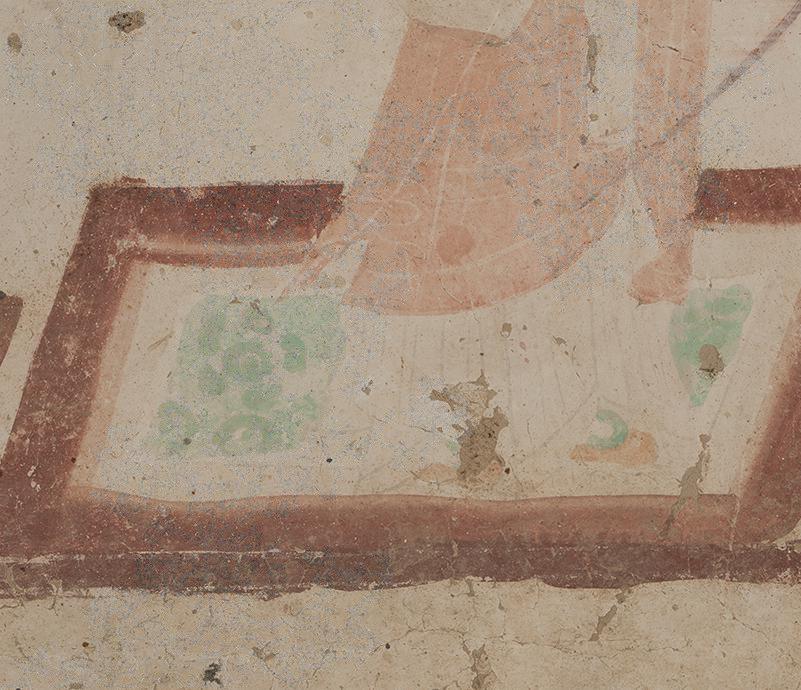

Supplement: Supplementary file 1 [file sensors-21-02091-s001.zip › smartsensors_supplementary_data/nazerietal-model-results/217_masked_1105380_1.jpg]

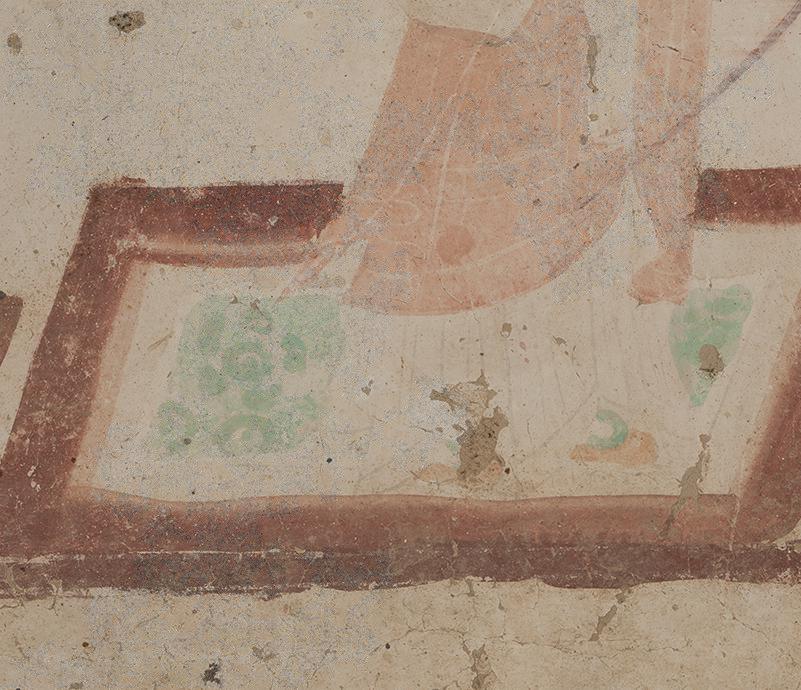

Supplement: Supplementary file 1 [file sensors-21-02091-s001.zip › smartsensors_supplementary_data/nazerietal-model-results/217_masked_1105380_2.jpg]

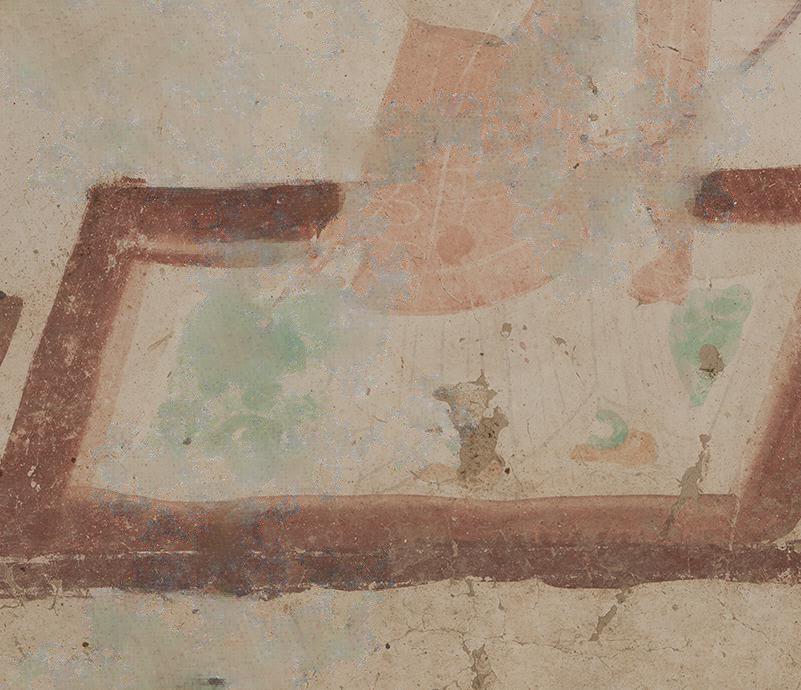

Supplement: Supplementary file 1 [file sensors-21-02091-s001.zip › smartsensors_supplementary_data/nazerietal-model-results/217_masked_1105380_3.jpg]

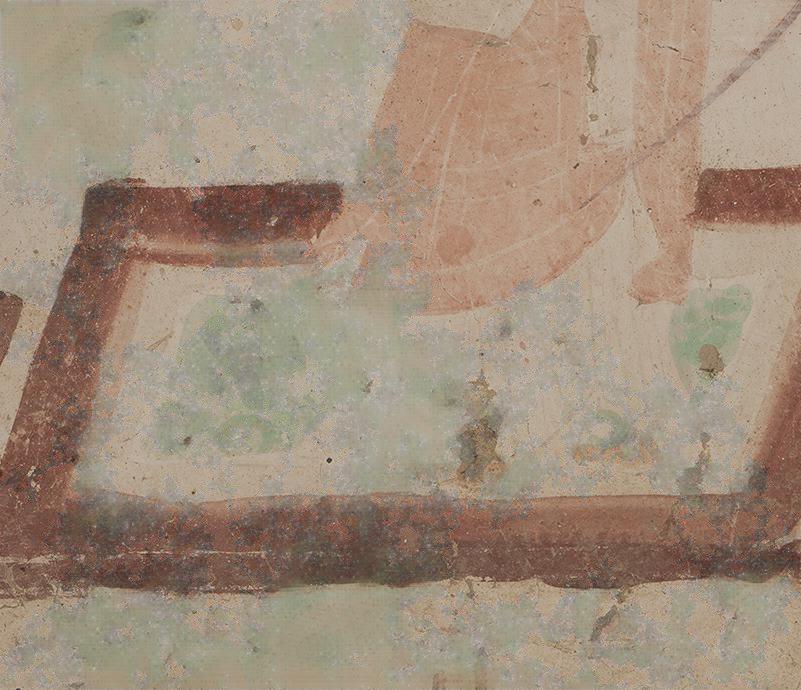

Supplement: Supplementary file 1 [file sensors-21-02091-s001.zip › smartsensors_supplementary_data/nazerietal-model-results/217_masked_1934415_0.jpg]

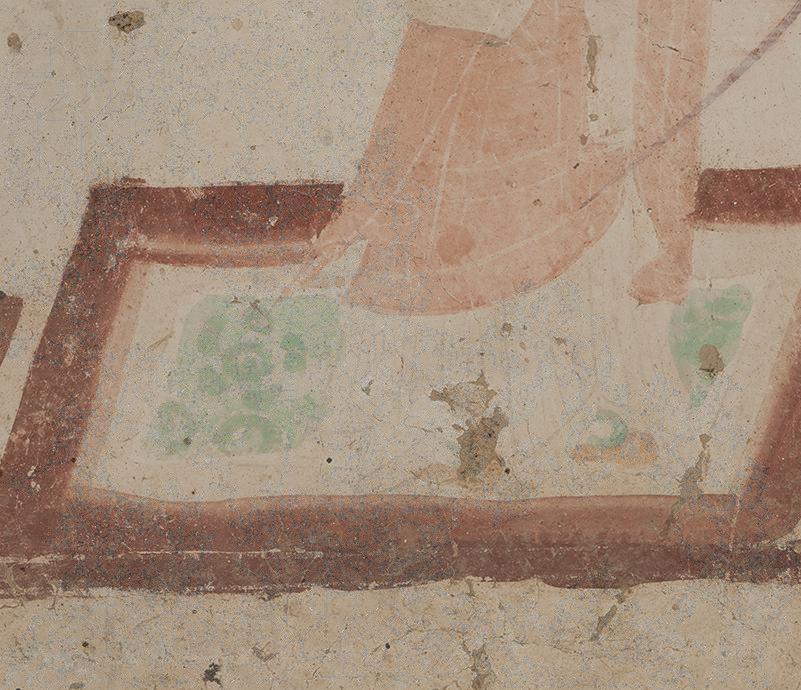

Supplement: Supplementary file 1 [file sensors-21-02091-s001.zip › smartsensors_supplementary_data/nazerietal-model-results/217_masked_1934415_1.jpg]

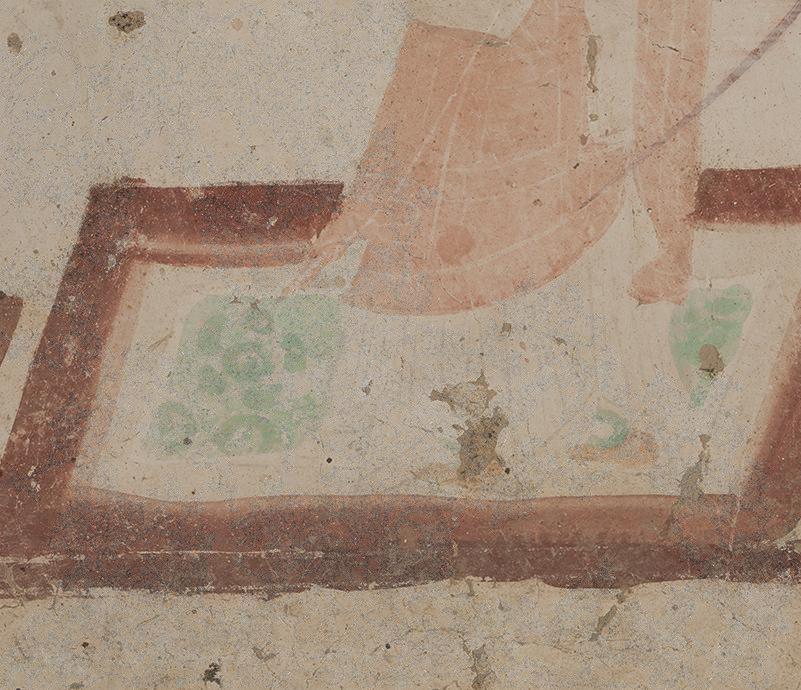

Supplement: Supplementary file 1 [file sensors-21-02091-s001.zip › smartsensors_supplementary_data/nazerietal-model-results/217_masked_1934415_2.jpg]

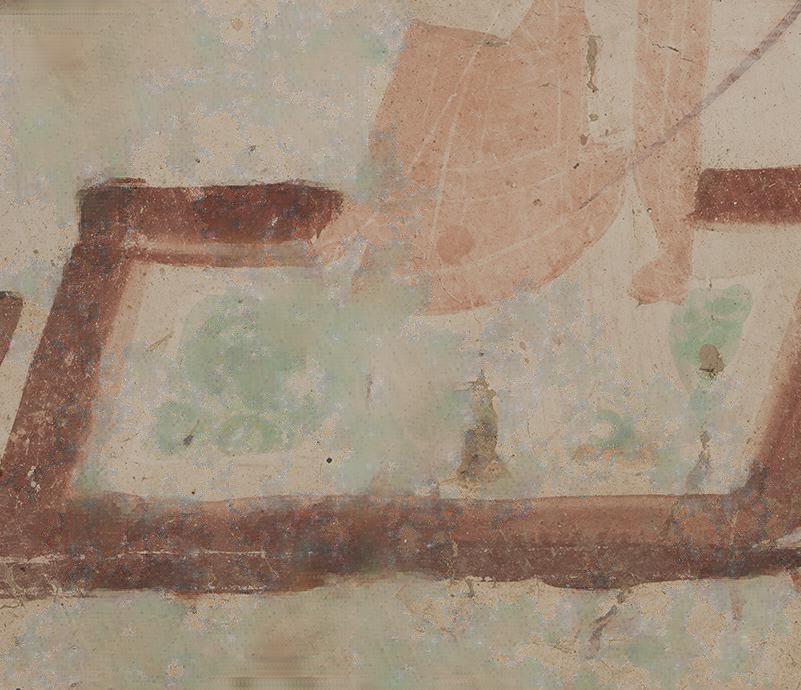

Supplement: Supplementary file 1 [file sensors-21-02091-s001.zip › smartsensors_supplementary_data/nazerietal-model-results/217_masked_1934415_3.jpg]

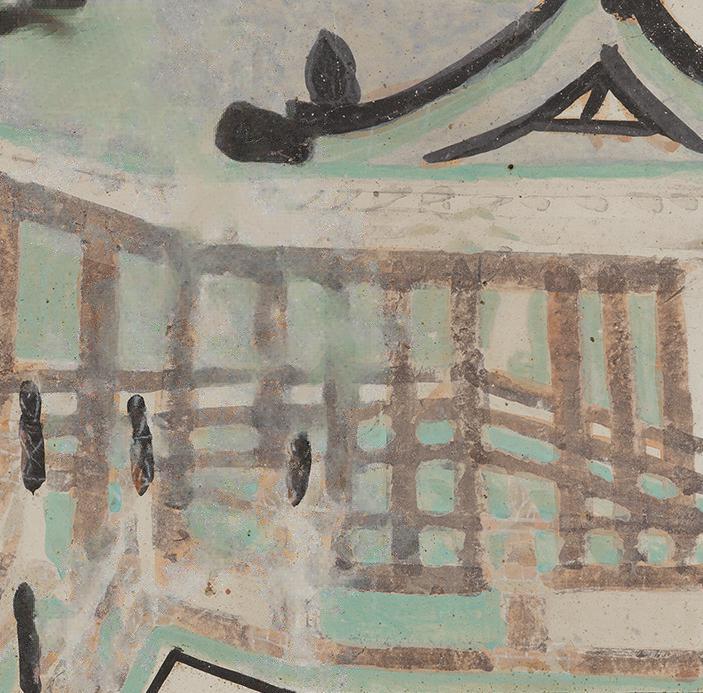

Supplement: Supplementary file 1 [file sensors-21-02091-s001.zip › smartsensors_supplementary_data/nazerietal-model-results/258_masked_1705126_0.jpg]

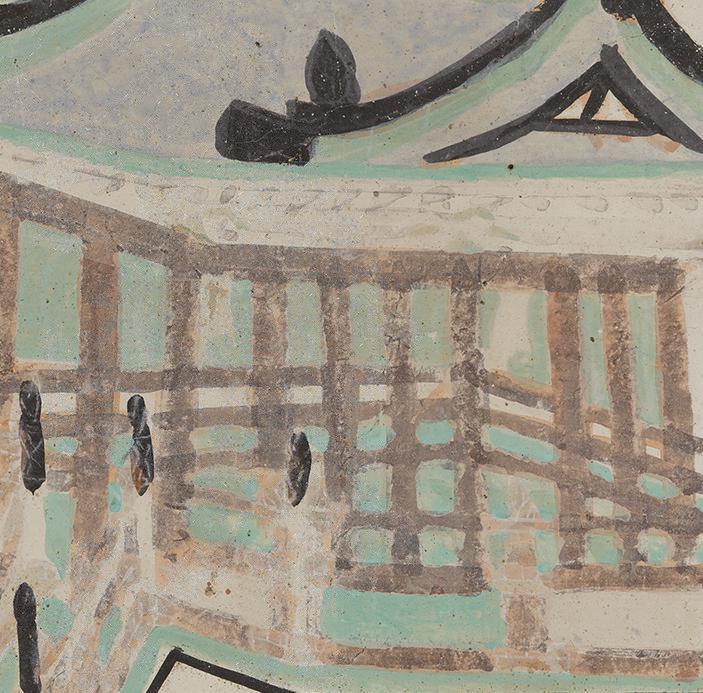

Supplement: Supplementary file 1 [file sensors-21-02091-s001.zip › smartsensors_supplementary_data/nazerietal-model-results/258_masked_1705126_1.jpg]

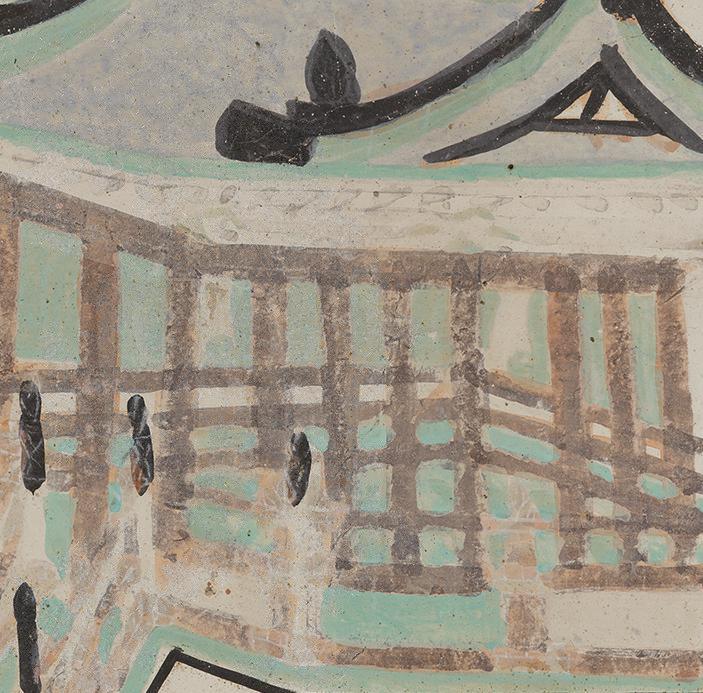

Supplement: Supplementary file 1 [file sensors-21-02091-s001.zip › smartsensors_supplementary_data/nazerietal-model-results/258_masked_1705126_2.jpg]

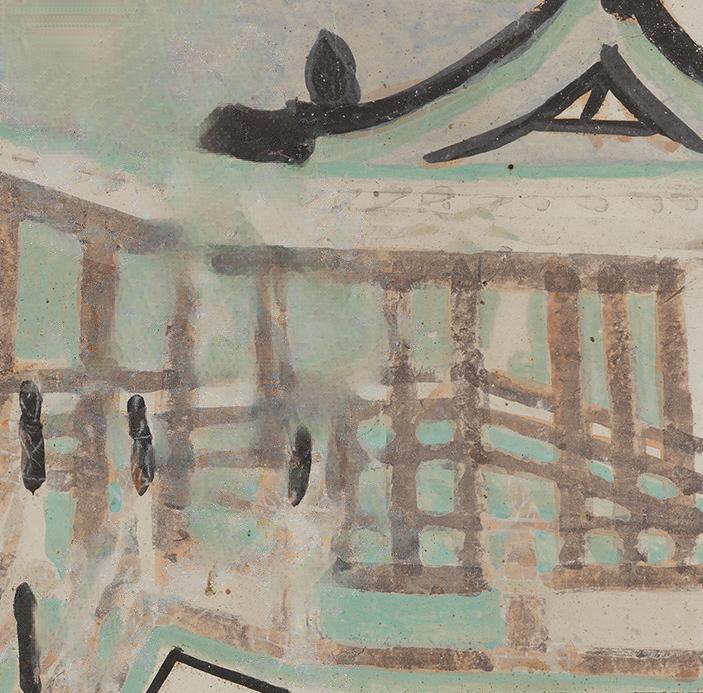

Supplement: Supplementary file 1 [file sensors-21-02091-s001.zip › smartsensors_supplementary_data/nazerietal-model-results/258_masked_1705126_3.jpg]

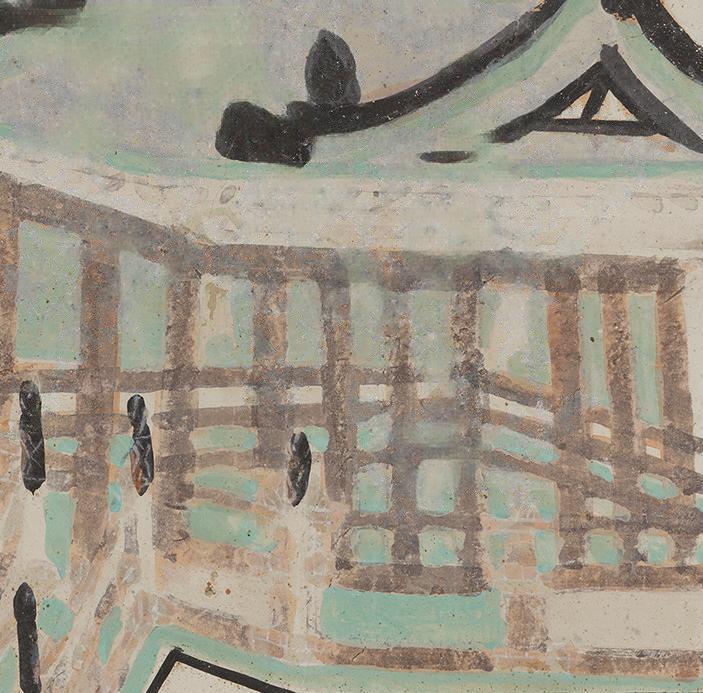

Supplement: Supplementary file 1 [file sensors-21-02091-s001.zip › smartsensors_supplementary_data/nazerietal-model-results/258_masked_974358_0.jpg]

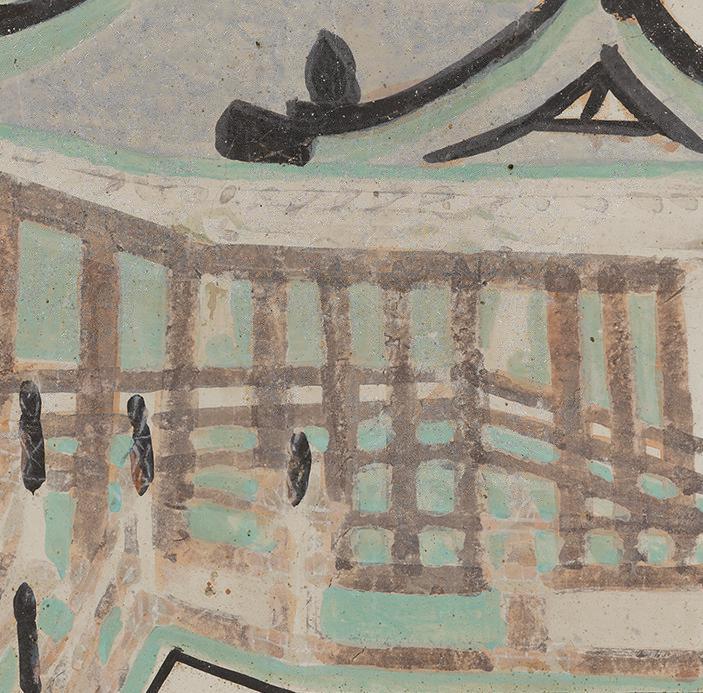

Supplement: Supplementary file 1 [file sensors-21-02091-s001.zip › smartsensors_supplementary_data/nazerietal-model-results/258_masked_974358_1.jpg]

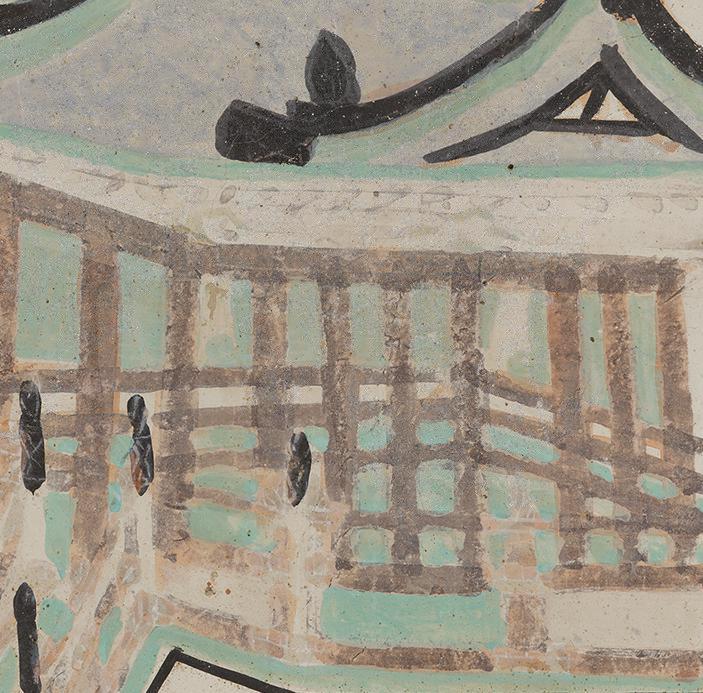

Supplement: Supplementary file 1 [file sensors-21-02091-s001.zip › smartsensors_supplementary_data/nazerietal-model-results/258_masked_974358_2.jpg]

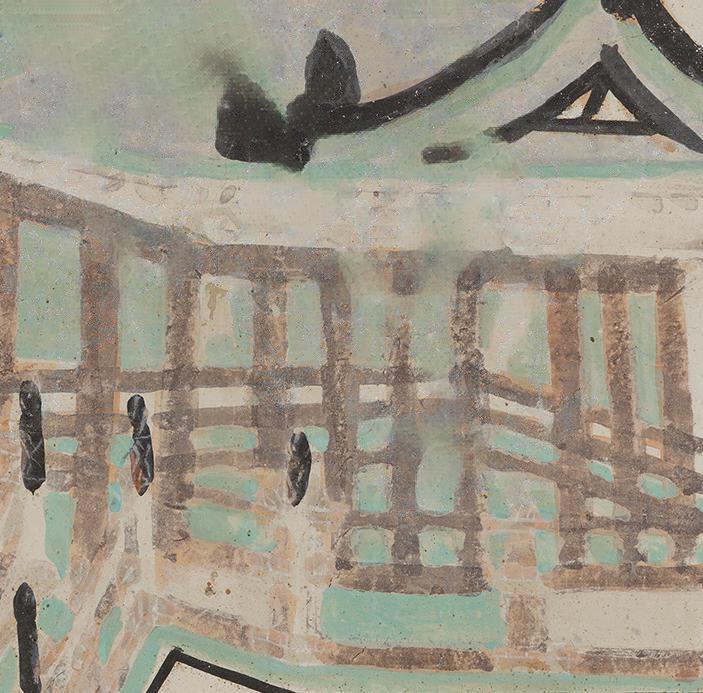

Supplement: Supplementary file 1 [file sensors-21-02091-s001.zip › smartsensors_supplementary_data/nazerietal-model-results/258_masked_974358_3.jpg]

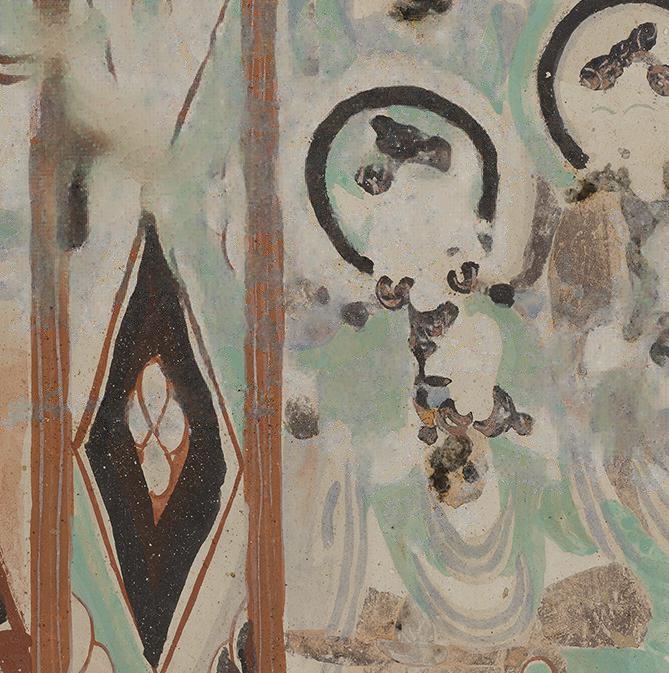

Supplement: Supplementary file 1 [file sensors-21-02091-s001.zip › smartsensors_supplementary_data/nazerietal-model-results/280_masked_1575829_0.jpg]

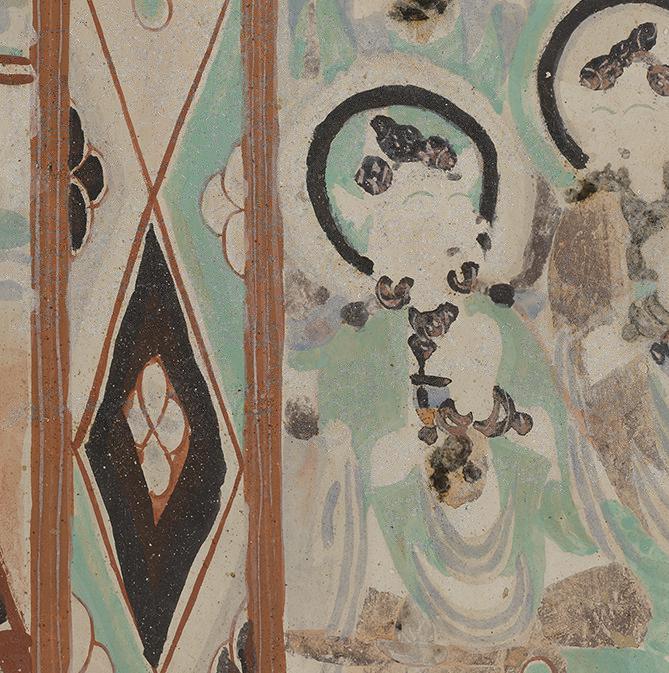

Supplement: Supplementary file 1 [file sensors-21-02091-s001.zip › smartsensors_supplementary_data/nazerietal-model-results/280_masked_1575829_1.jpg]

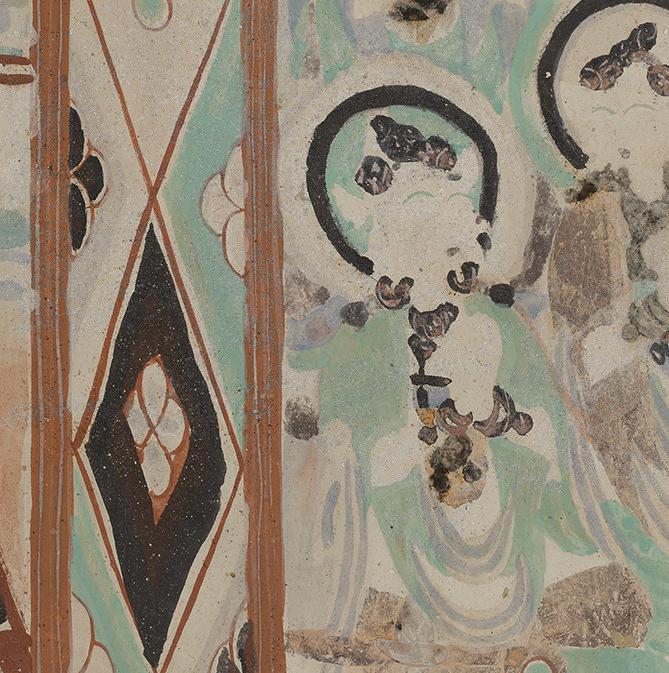

Supplement: Supplementary file 1 [file sensors-21-02091-s001.zip › smartsensors_supplementary_data/nazerietal-model-results/280_masked_1575829_2.jpg]

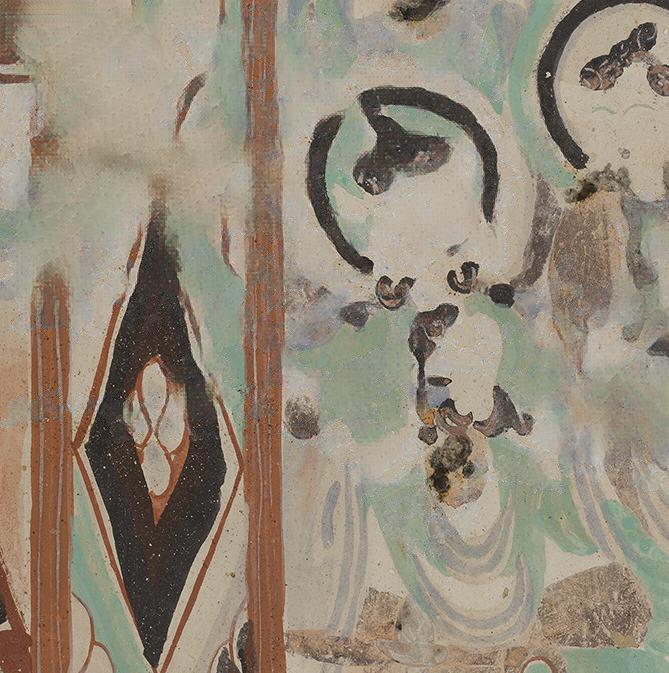

Supplement: Supplementary file 1 [file sensors-21-02091-s001.zip › smartsensors_supplementary_data/nazerietal-model-results/280_masked_1575829_3.jpg]

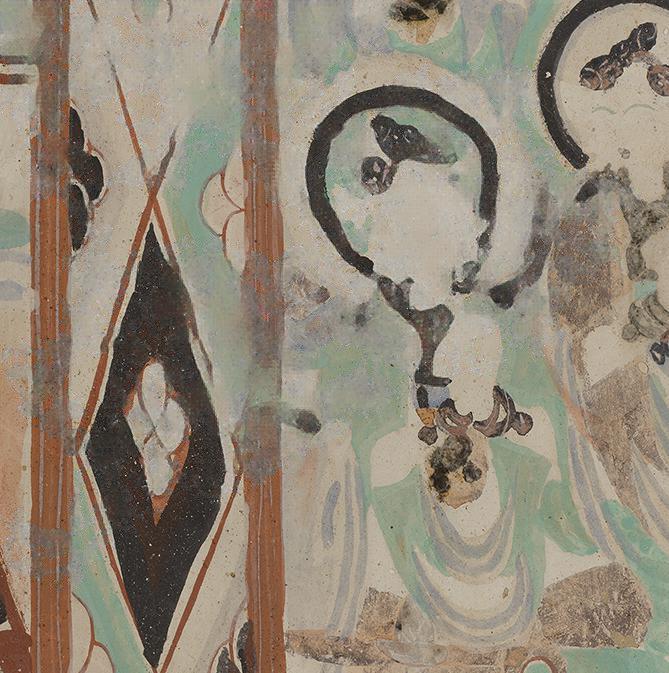

Supplement: Supplementary file 1 [file sensors-21-02091-s001.zip › smartsensors_supplementary_data/nazerietal-model-results/280_masked_900474_0.jpg]

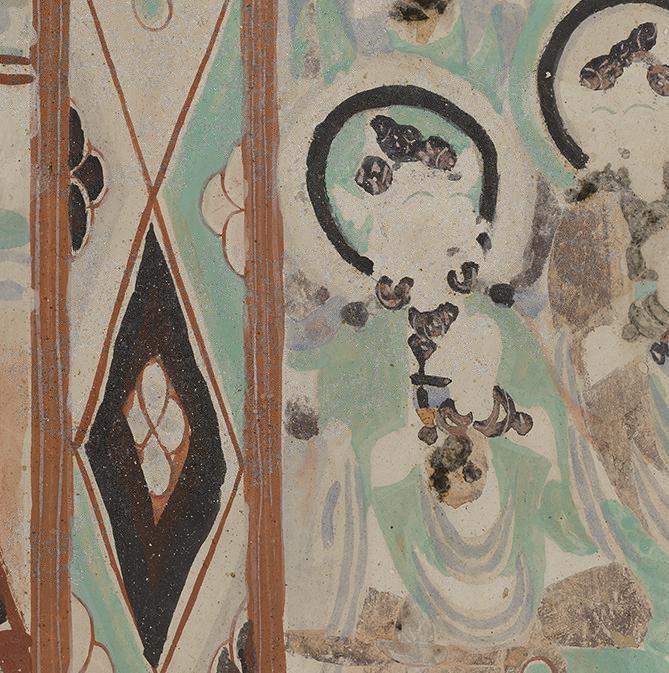

Supplement: Supplementary file 1 [file sensors-21-02091-s001.zip › smartsensors_supplementary_data/nazerietal-model-results/280_masked_900474_1.jpg]

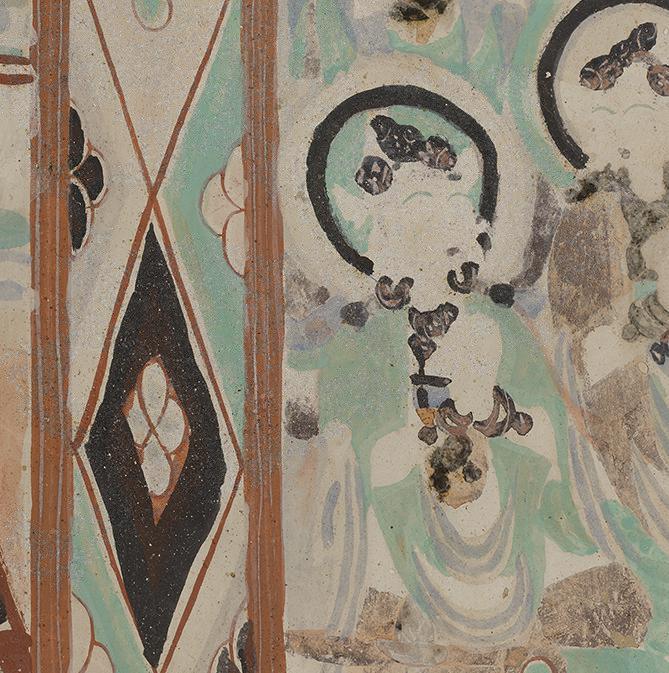

Supplement: Supplementary file 1 [file sensors-21-02091-s001.zip › smartsensors_supplementary_data/nazerietal-model-results/280_masked_900474_2.jpg]

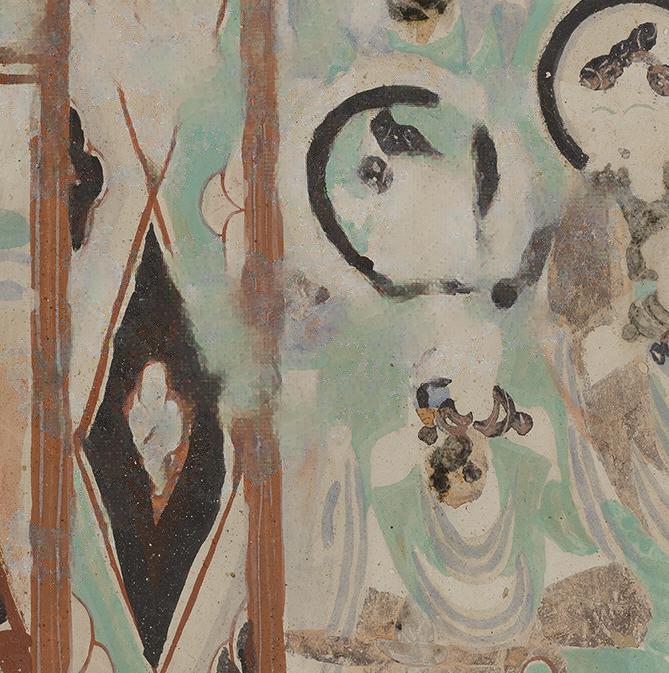

Supplement: Supplementary file 1 [file sensors-21-02091-s001.zip › smartsensors_supplementary_data/nazerietal-model-results/280_masked_900474_3.jpg]

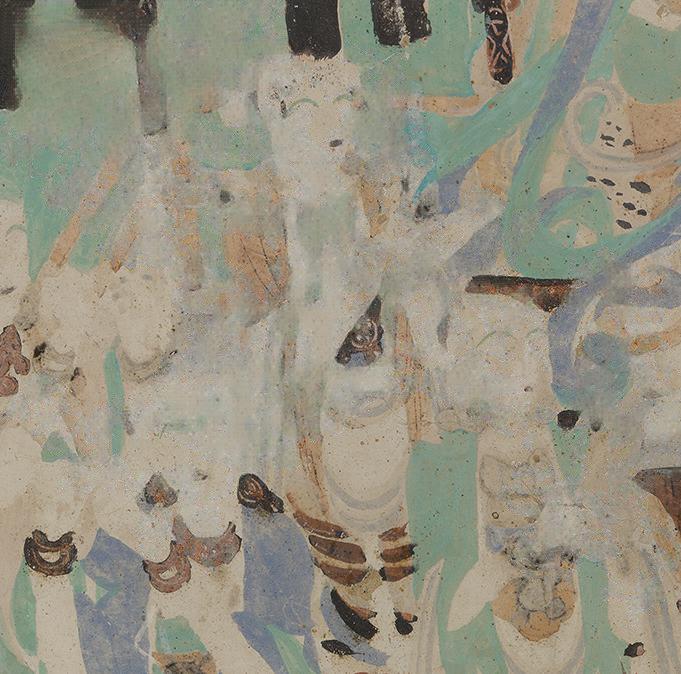

Supplement: Supplementary file 1 [file sensors-21-02091-s001.zip › smartsensors_supplementary_data/nazerietal-model-results/306_masked_1606479_0.jpg]

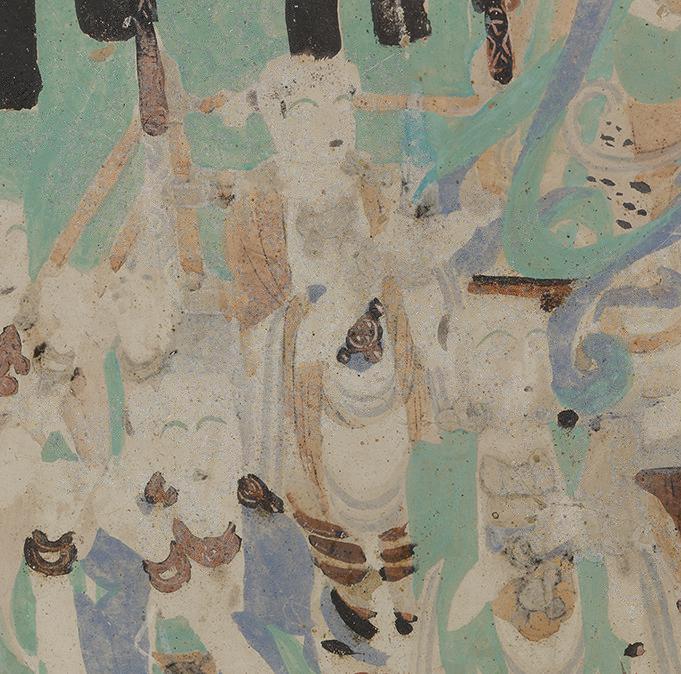

Supplement: Supplementary file 1 [file sensors-21-02091-s001.zip › smartsensors_supplementary_data/nazerietal-model-results/306_masked_1606479_1.jpg]

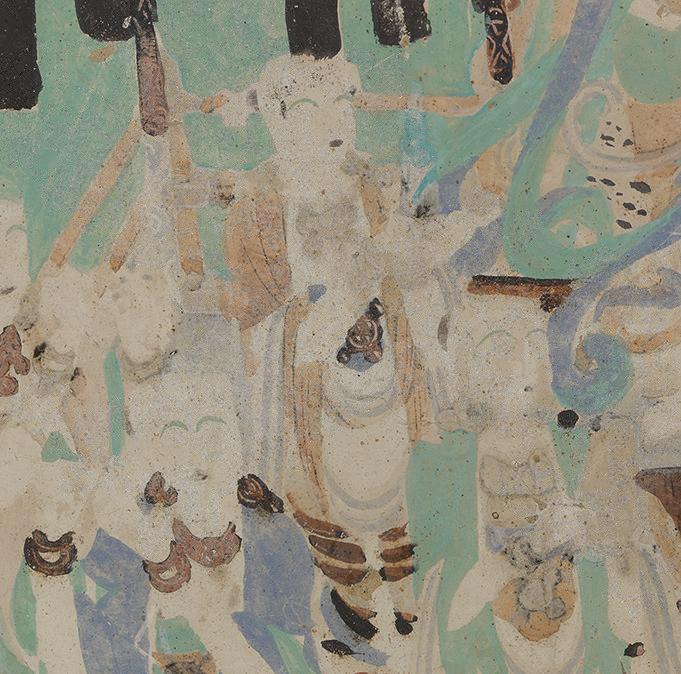

Supplement: Supplementary file 1 [file sensors-21-02091-s001.zip › smartsensors_supplementary_data/nazerietal-model-results/306_masked_1606479_2.jpg]

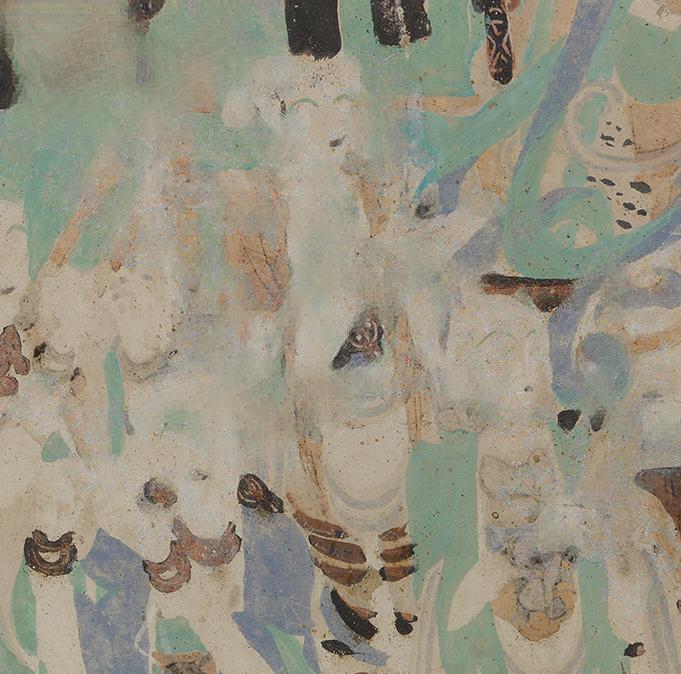

Supplement: Supplementary file 1 [file sensors-21-02091-s001.zip › smartsensors_supplementary_data/nazerietal-model-results/306_masked_1606479_3.jpg]

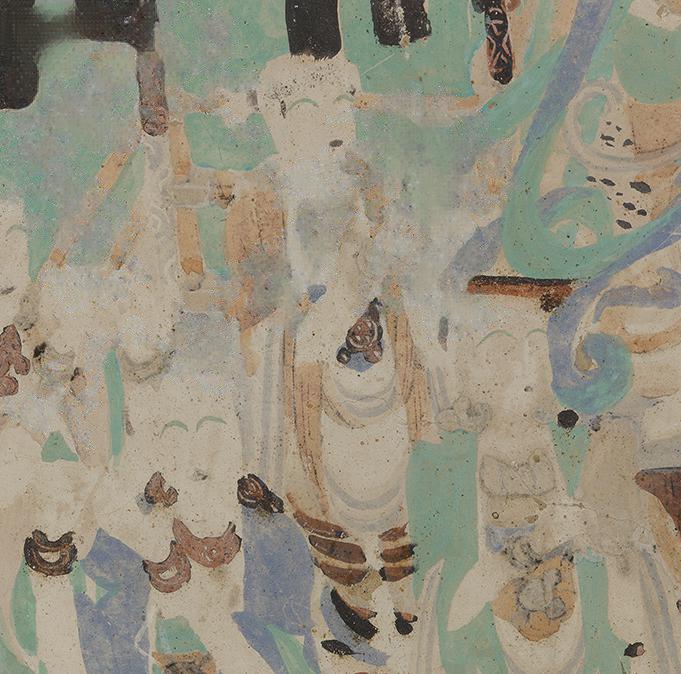

Supplement: Supplementary file 1 [file sensors-21-02091-s001.zip › smartsensors_supplementary_data/nazerietal-model-results/306_masked_917988_0.jpg]

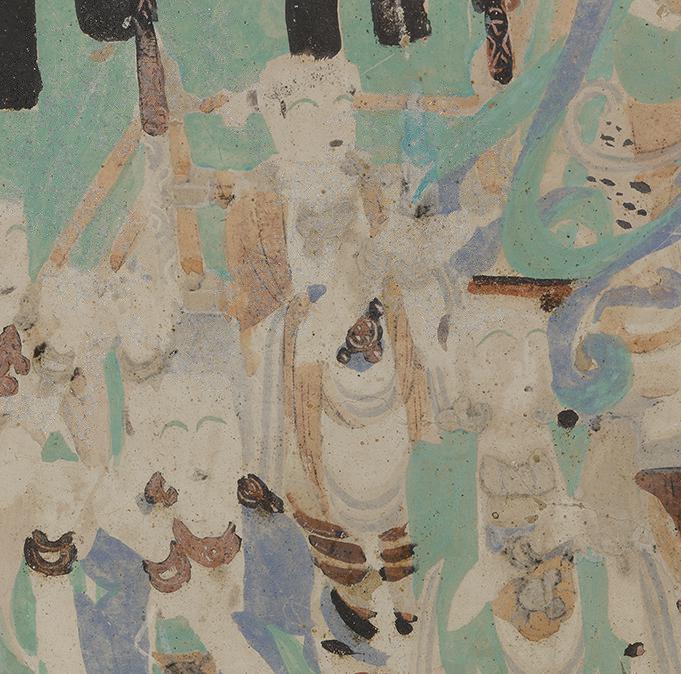

Supplement: Supplementary file 1 [file sensors-21-02091-s001.zip › smartsensors_supplementary_data/nazerietal-model-results/306_masked_917988_1.jpg]

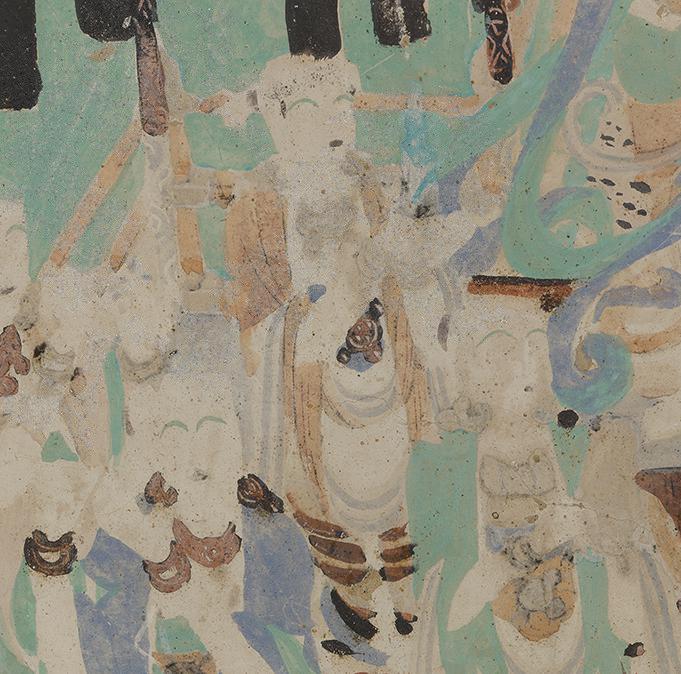

Supplement: Supplementary file 1 [file sensors-21-02091-s001.zip › smartsensors_supplementary_data/nazerietal-model-results/306_masked_917988_2.jpg]

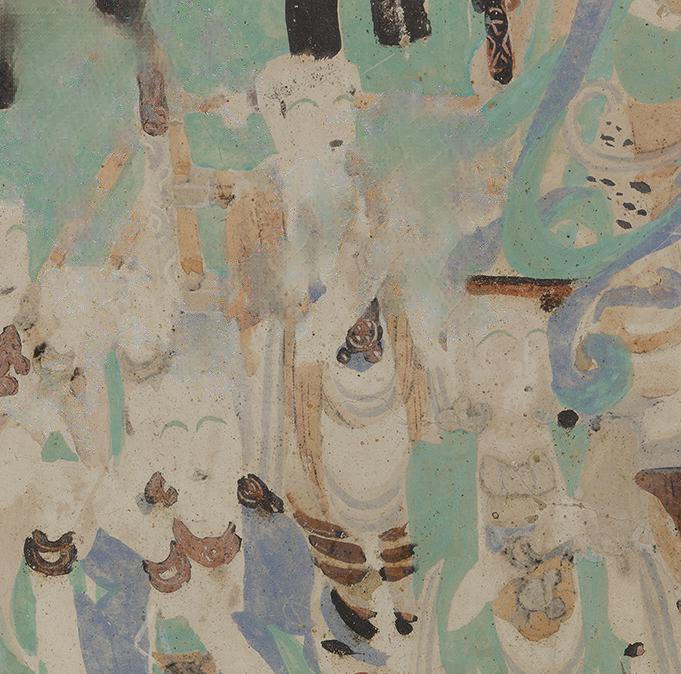

Supplement: Supplementary file 1 [file sensors-21-02091-s001.zip › smartsensors_supplementary_data/nazerietal-model-results/306_masked_917988_3.jpg]

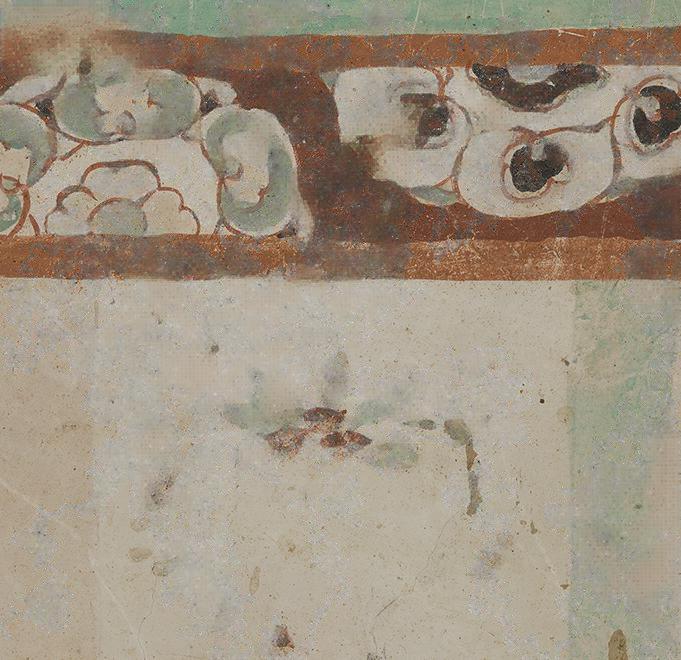

Supplement: Supplementary file 1 [file sensors-21-02091-s001.zip › smartsensors_supplementary_data/nazerietal-model-results/325_masked_1573110_0.jpg]

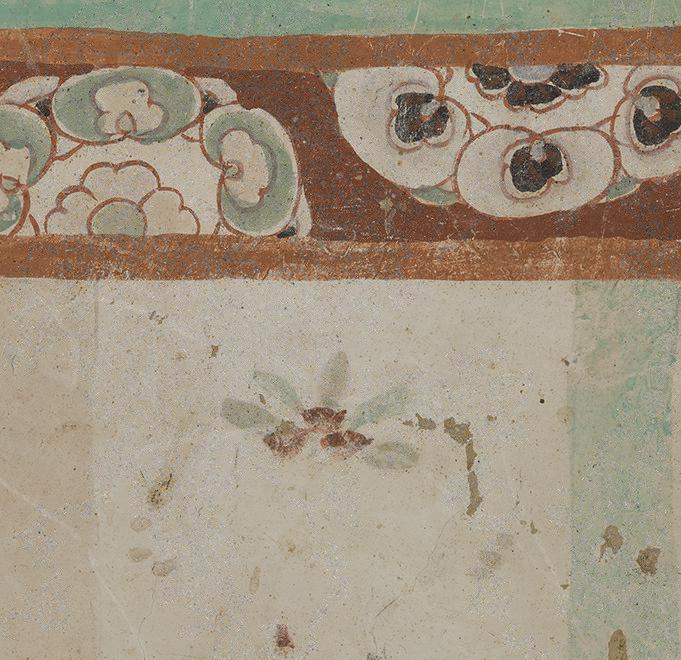

Supplement: Supplementary file 1 [file sensors-21-02091-s001.zip › smartsensors_supplementary_data/nazerietal-model-results/325_masked_1573110_1.jpg]

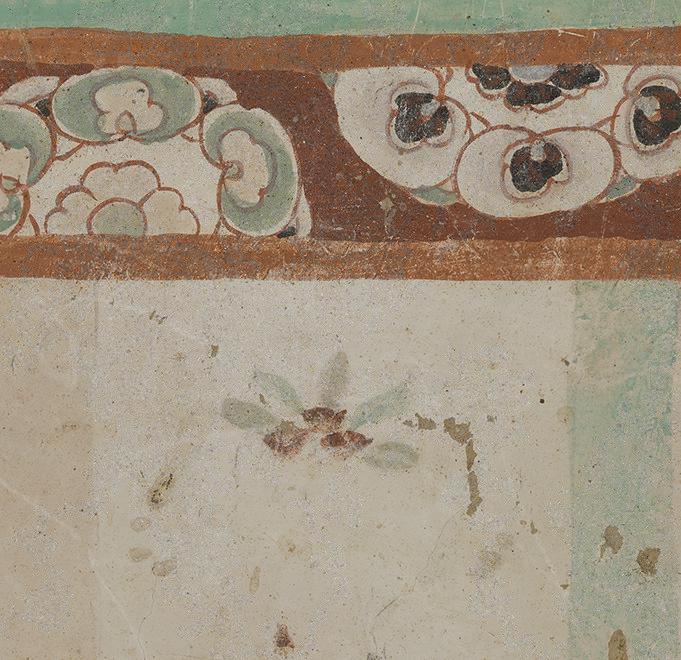

Supplement: Supplementary file 1 [file sensors-21-02091-s001.zip › smartsensors_supplementary_data/nazerietal-model-results/325_masked_1573110_2.jpg]

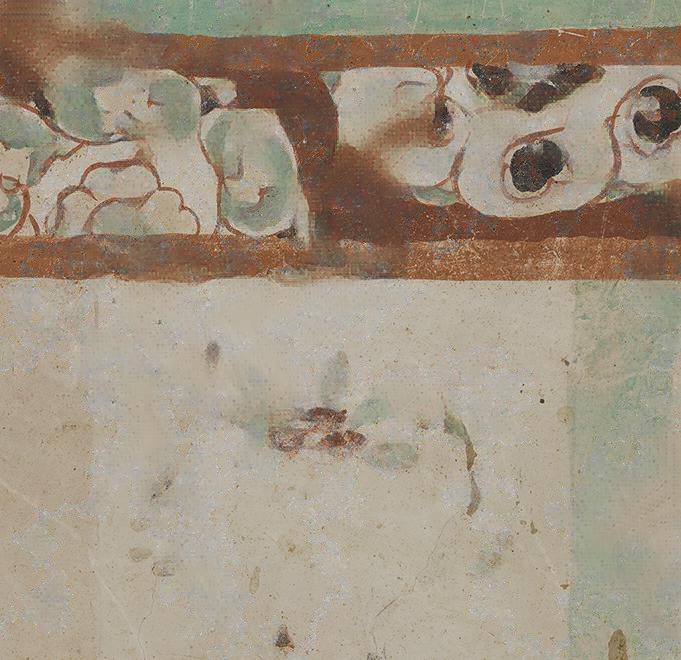

Supplement: Supplementary file 1 [file sensors-21-02091-s001.zip › smartsensors_supplementary_data/nazerietal-model-results/325_masked_1573110_3.jpg]

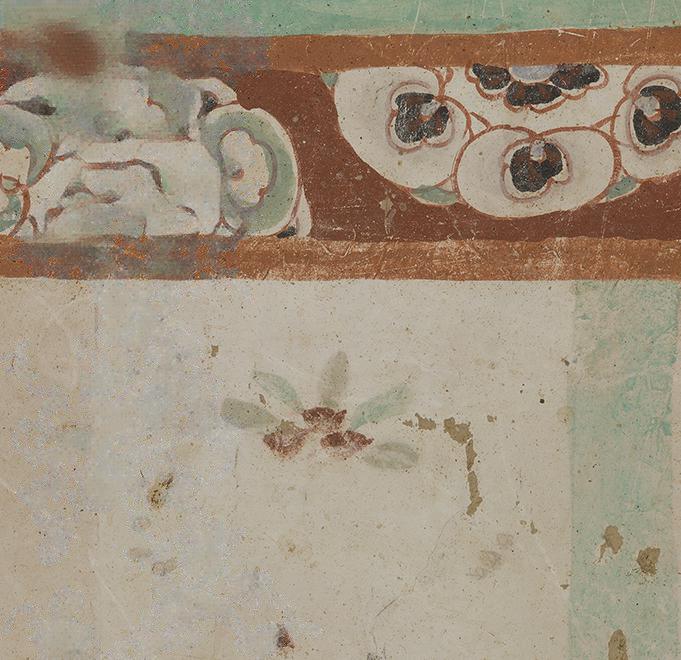

Supplement: Supplementary file 1 [file sensors-21-02091-s001.zip › smartsensors_supplementary_data/nazerietal-model-results/325_masked_898920_0.jpg]

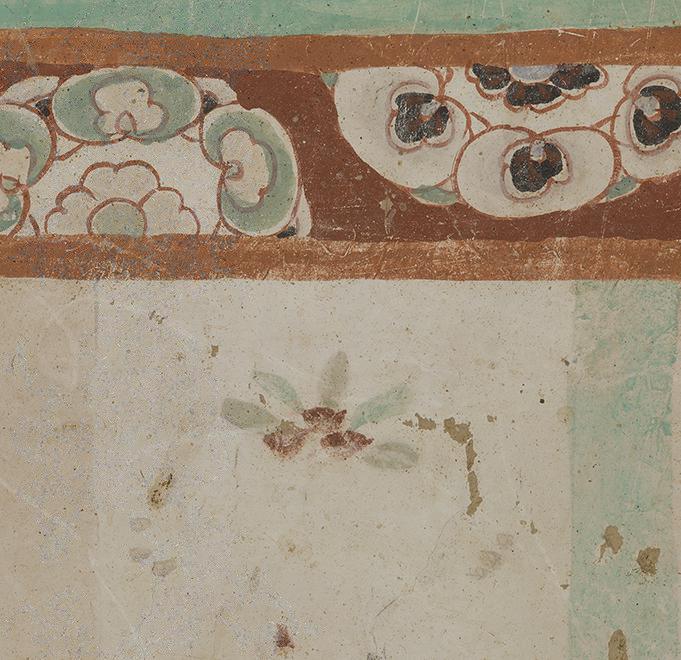

Supplement: Supplementary file 1 [file sensors-21-02091-s001.zip › smartsensors_supplementary_data/nazerietal-model-results/325_masked_898920_1.jpg]

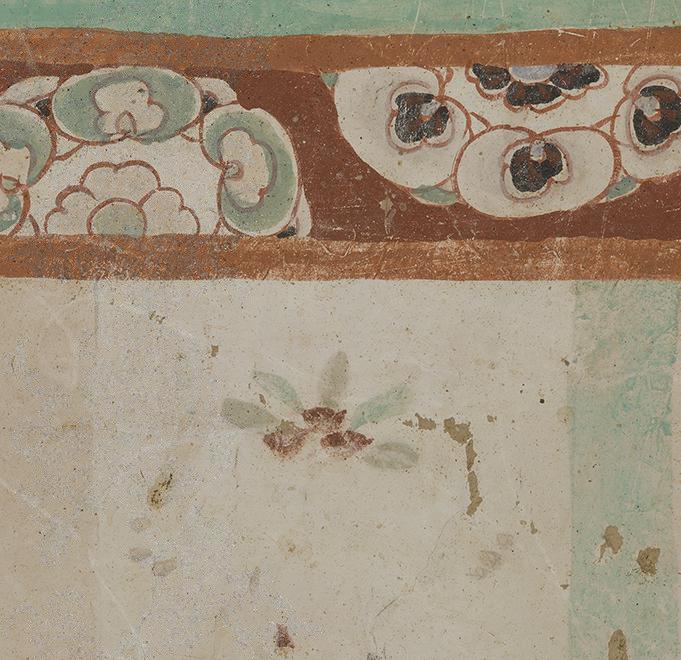

Supplement: Supplementary file 1 [file sensors-21-02091-s001.zip › smartsensors_supplementary_data/nazerietal-model-results/325_masked_898920_2.jpg]

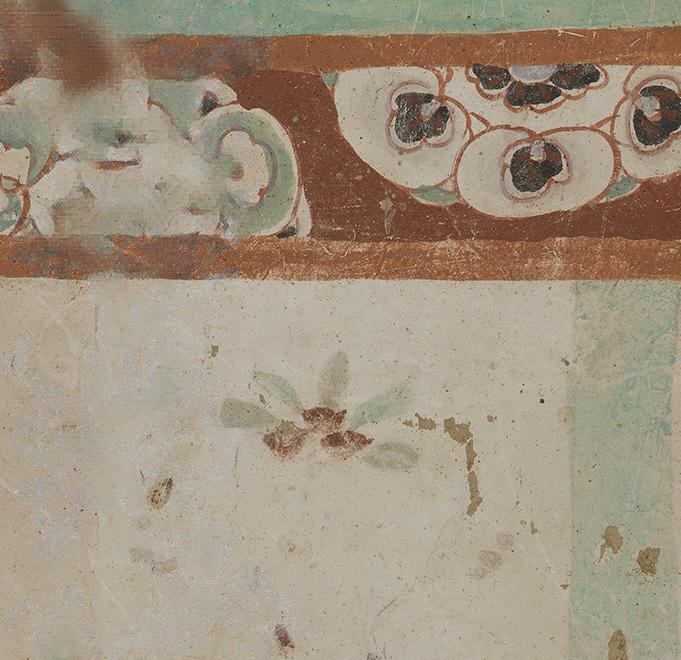

Supplement: Supplementary file 1 [file sensors-21-02091-s001.zip › smartsensors_supplementary_data/nazerietal-model-results/325_masked_898920_3.jpg]

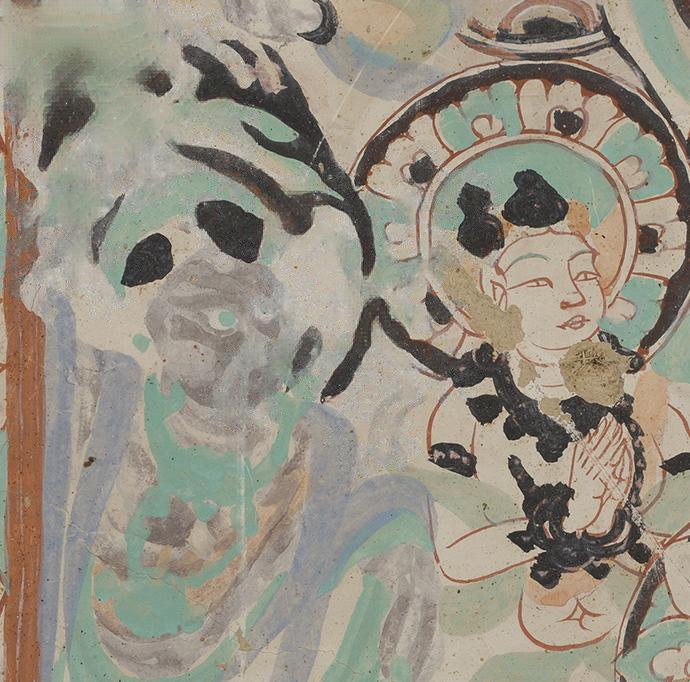

Supplement: Supplementary file 1 [file sensors-21-02091-s001.zip › smartsensors_supplementary_data/nazerietal-model-results/356_masked_1647030_0.jpg]

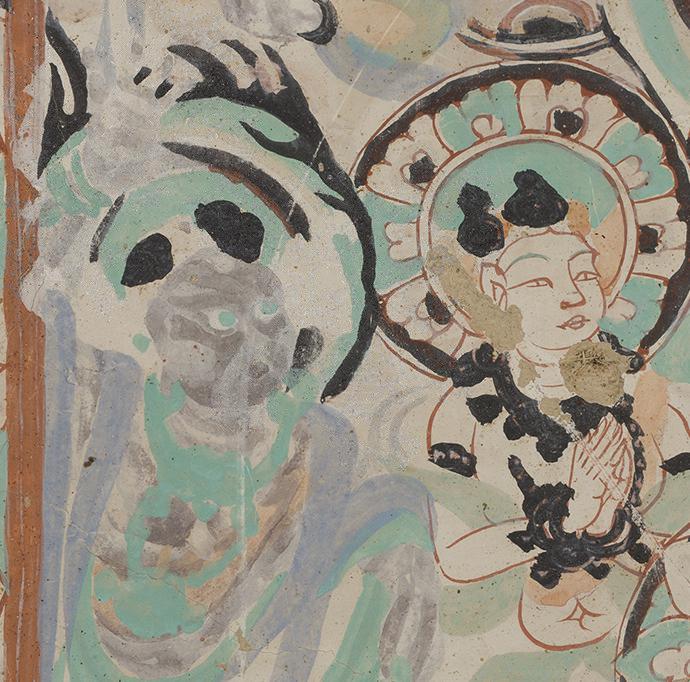

Supplement: Supplementary file 1 [file sensors-21-02091-s001.zip › smartsensors_supplementary_data/nazerietal-model-results/356_masked_1647030_1.jpg]

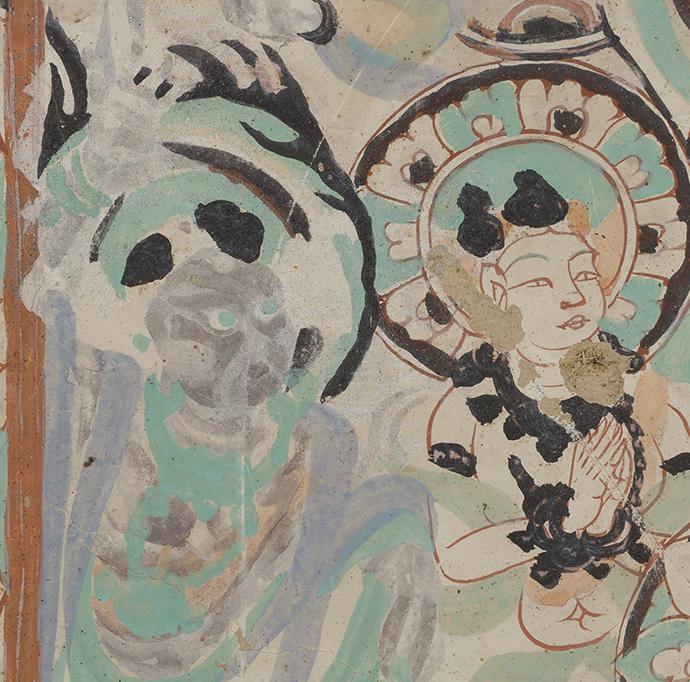

Supplement: Supplementary file 1 [file sensors-21-02091-s001.zip › smartsensors_supplementary_data/nazerietal-model-results/356_masked_1647030_2.jpg]

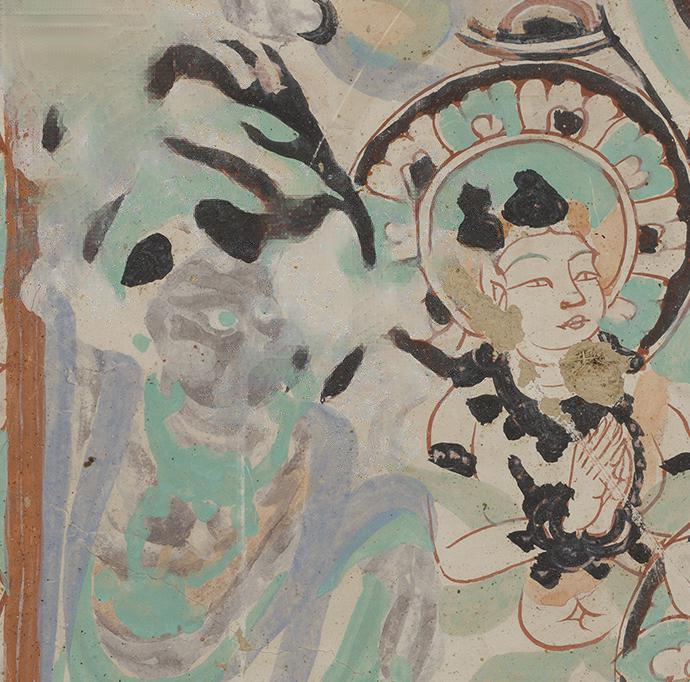

Supplement: Supplementary file 1 [file sensors-21-02091-s001.zip › smartsensors_supplementary_data/nazerietal-model-results/356_masked_1647030_3.jpg]

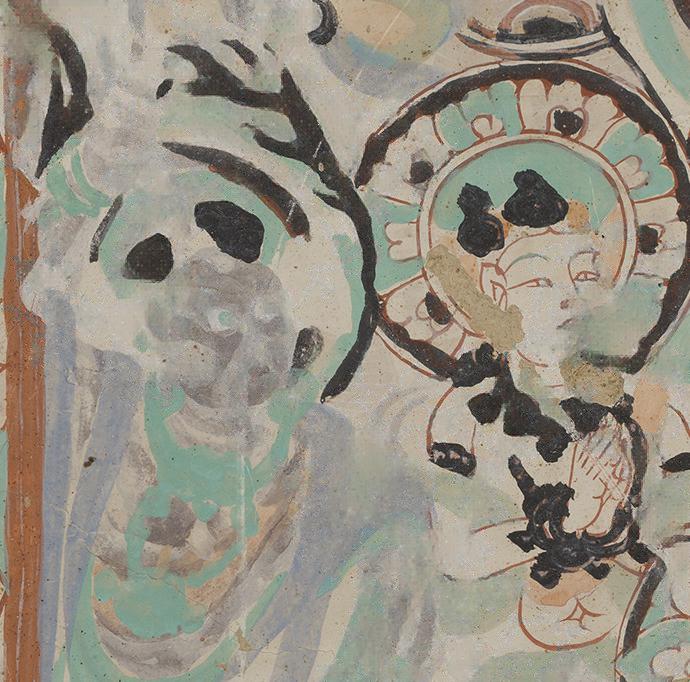

Supplement: Supplementary file 1 [file sensors-21-02091-s001.zip › smartsensors_supplementary_data/nazerietal-model-results/356_masked_941160_0.jpg]

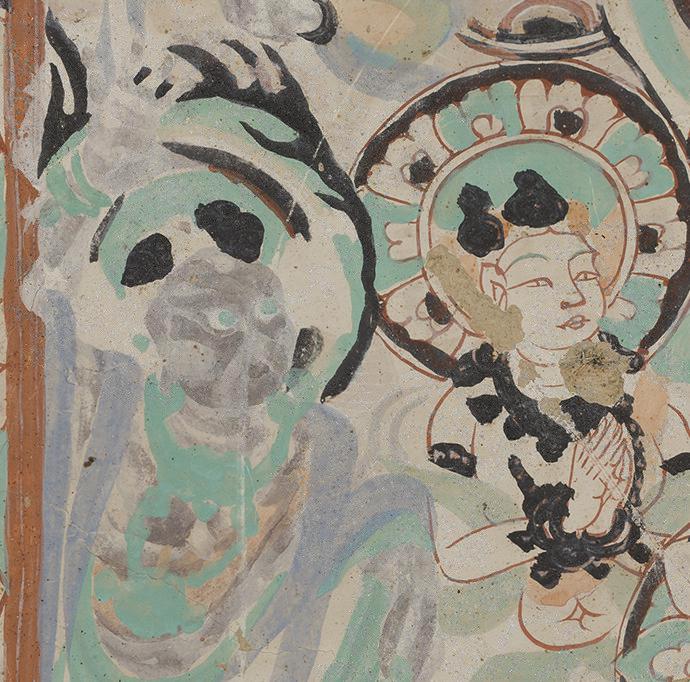

Supplement: Supplementary file 1 [file sensors-21-02091-s001.zip › smartsensors_supplementary_data/nazerietal-model-results/356_masked_941160_1.jpg]

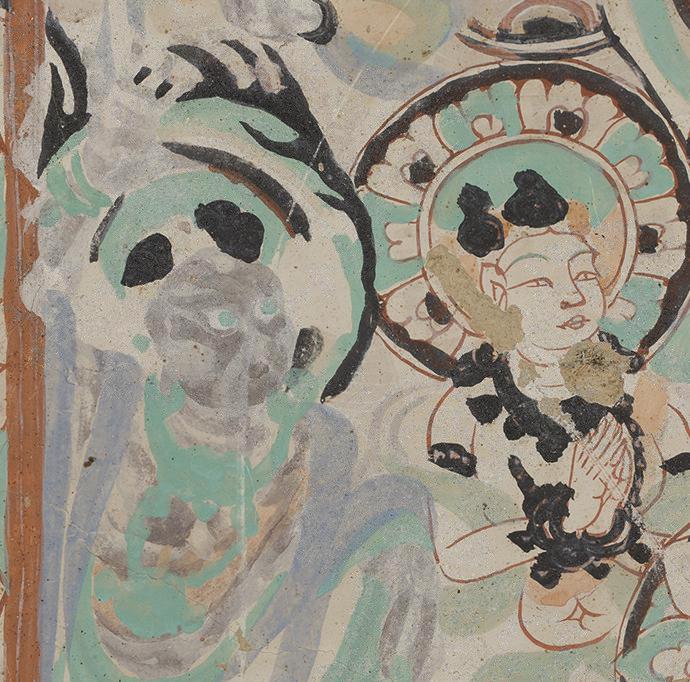

Supplement: Supplementary file 1 [file sensors-21-02091-s001.zip › smartsensors_supplementary_data/nazerietal-model-results/356_masked_941160_2.jpg]

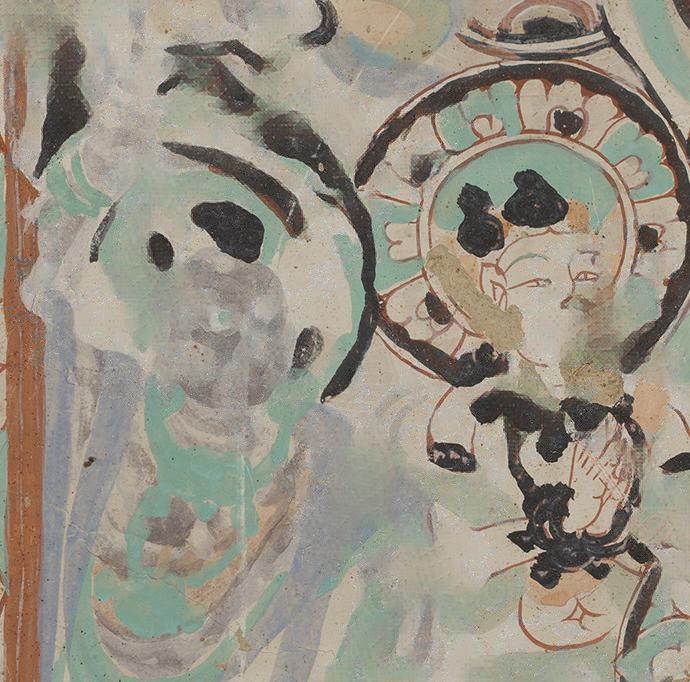

Supplement: Supplementary file 1 [file sensors-21-02091-s001.zip › smartsensors_supplementary_data/nazerietal-model-results/356_masked_941160_3.jpg]

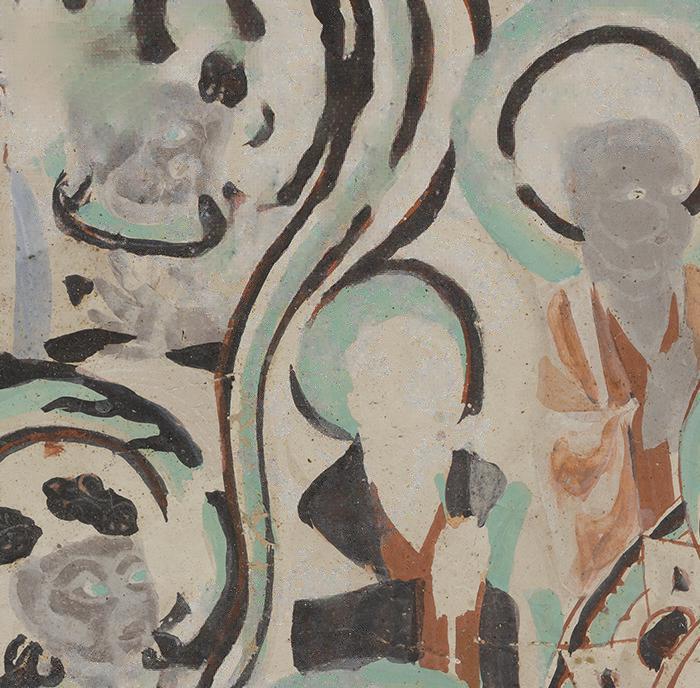

Supplement: Supplementary file 1 [file sensors-21-02091-s001.zip › smartsensors_supplementary_data/nazerietal-model-results/362_masked_1685600_0.jpg]

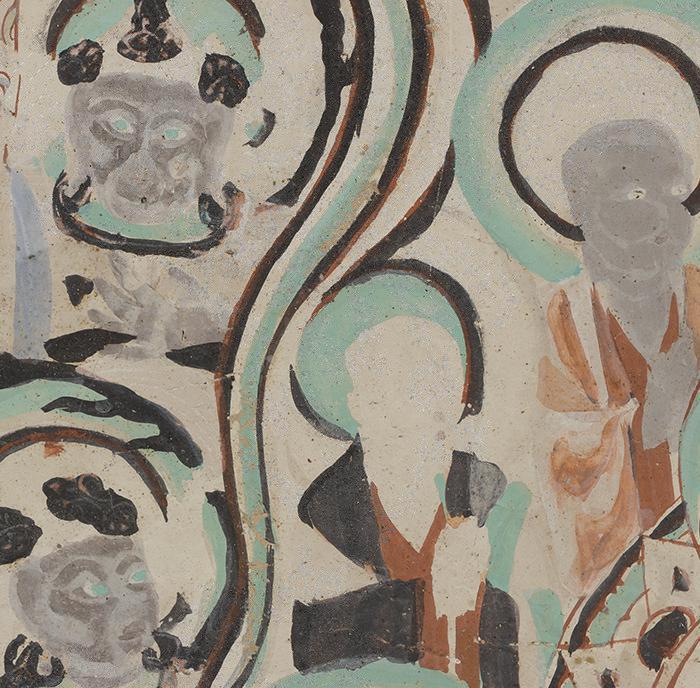

Supplement: Supplementary file 1 [file sensors-21-02091-s001.zip › smartsensors_supplementary_data/nazerietal-model-results/362_masked_1685600_1.jpg]

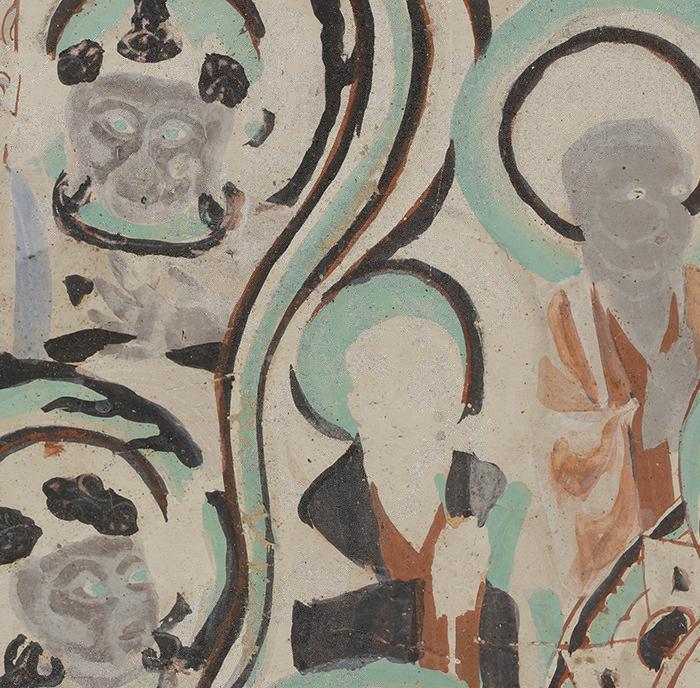

Supplement: Supplementary file 1 [file sensors-21-02091-s001.zip › smartsensors_supplementary_data/nazerietal-model-results/362_masked_1685600_2.jpg]

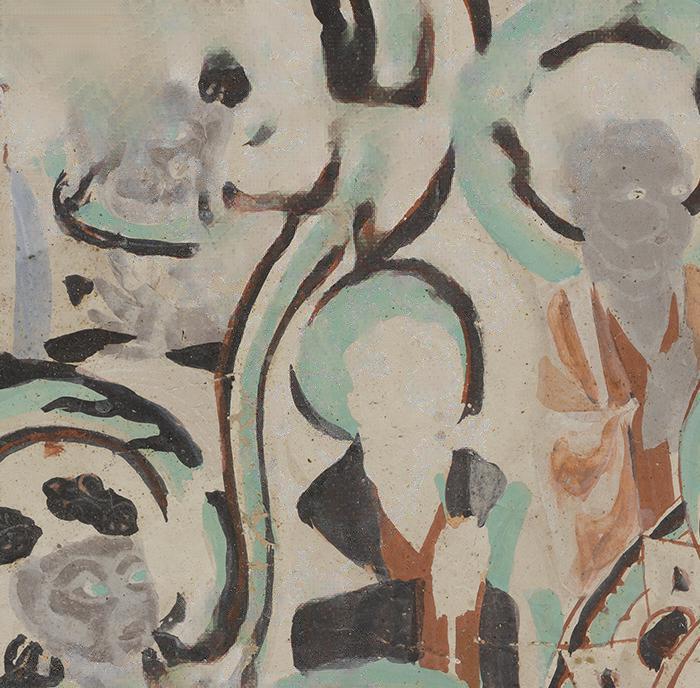

Supplement: Supplementary file 1 [file sensors-21-02091-s001.zip › smartsensors_supplementary_data/nazerietal-model-results/362_masked_1685600_3.jpg]

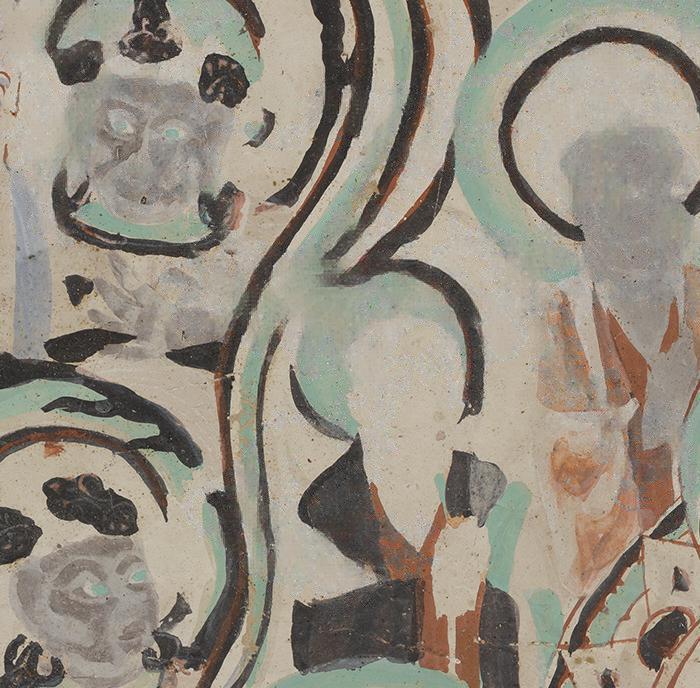

Supplement: Supplementary file 1 [file sensors-21-02091-s001.zip › smartsensors_supplementary_data/nazerietal-model-results/362_masked_963200_0.jpg]

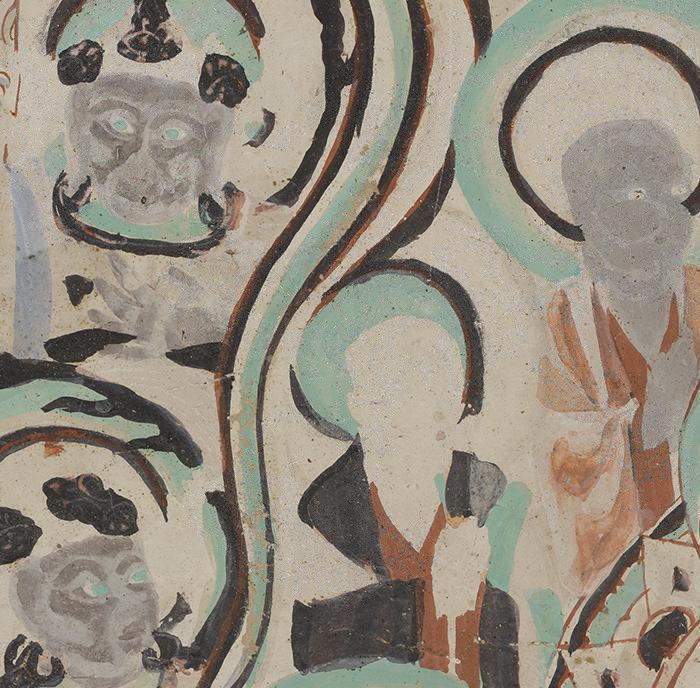

Supplement: Supplementary file 1 [file sensors-21-02091-s001.zip › smartsensors_supplementary_data/nazerietal-model-results/362_masked_963200_1.jpg]

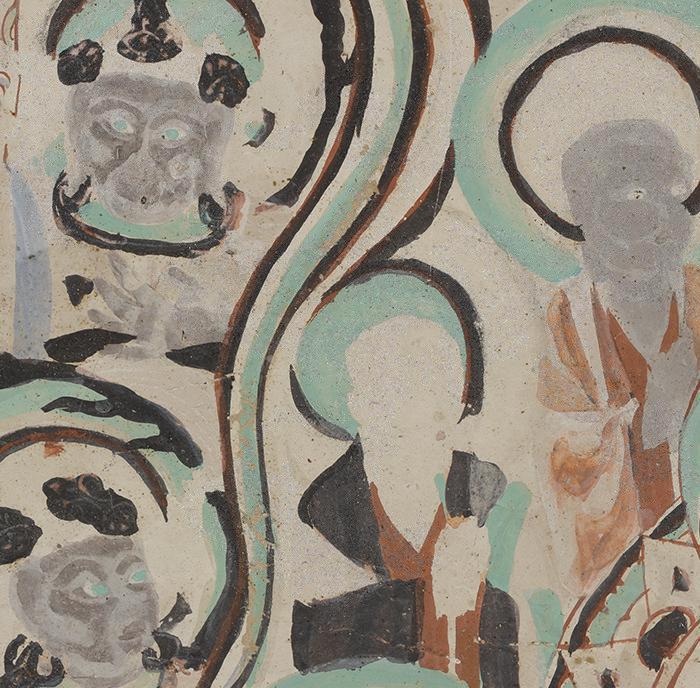

Supplement: Supplementary file 1 [file sensors-21-02091-s001.zip › smartsensors_supplementary_data/nazerietal-model-results/362_masked_963200_2.jpg]

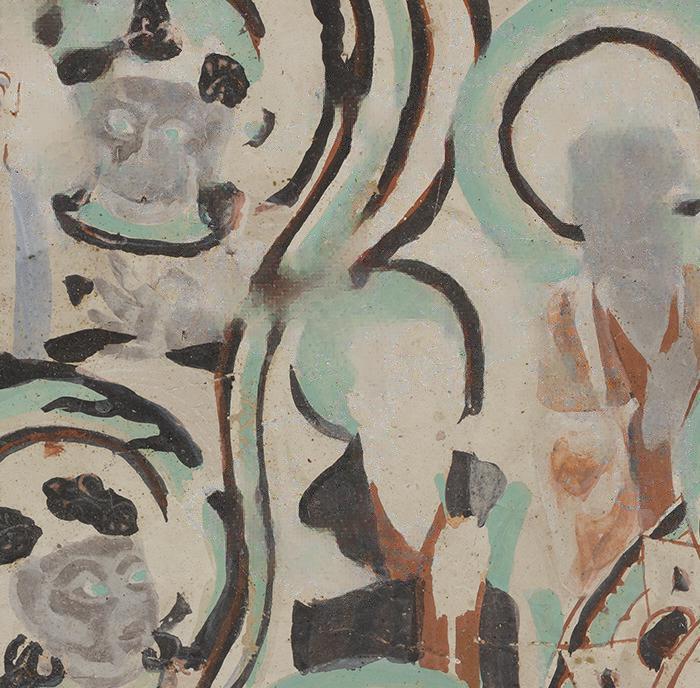

Supplement: Supplementary file 1 [file sensors-21-02091-s001.zip › smartsensors_supplementary_data/nazerietal-model-results/362_masked_963200_3.jpg]

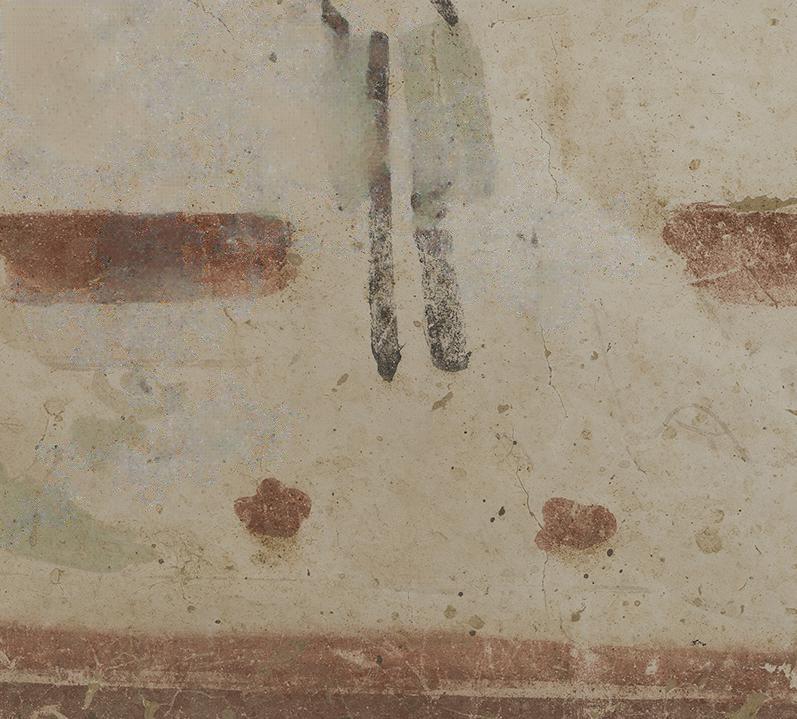

Supplement: Supplementary file 1 [file sensors-21-02091-s001.zip › smartsensors_supplementary_data/nazerietal-model-results/402_masked_1146086_0.jpg]

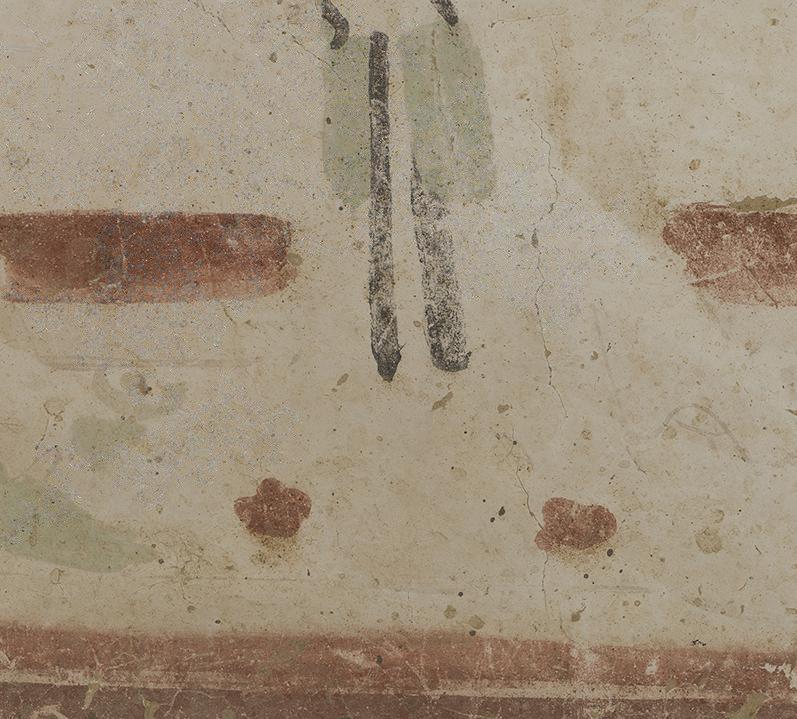

Supplement: Supplementary file 1 [file sensors-21-02091-s001.zip › smartsensors_supplementary_data/nazerietal-model-results/402_masked_1146086_1.jpg]

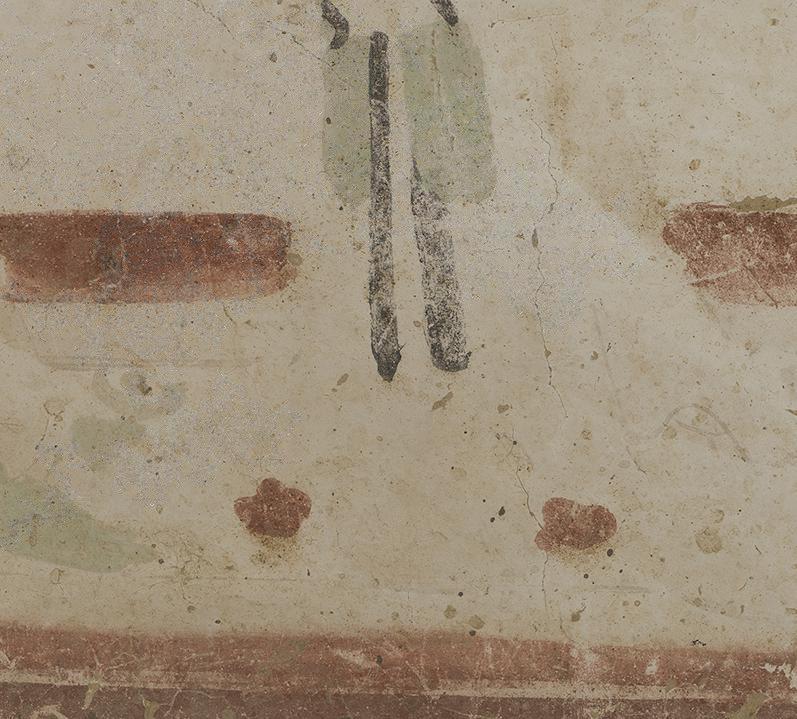

Supplement: Supplementary file 1 [file sensors-21-02091-s001.zip › smartsensors_supplementary_data/nazerietal-model-results/402_masked_1146086_2.jpg]

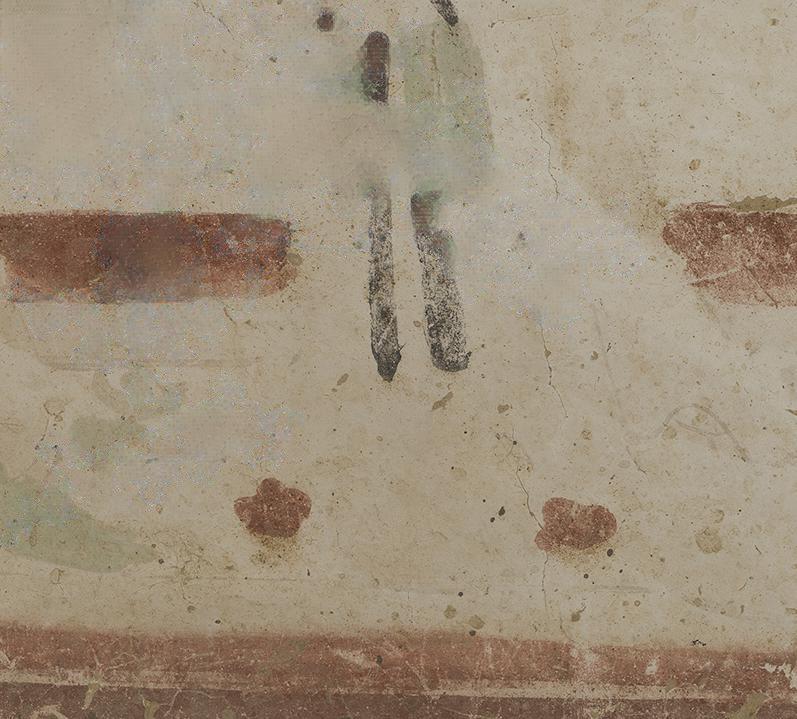

Supplement: Supplementary file 1 [file sensors-21-02091-s001.zip › smartsensors_supplementary_data/nazerietal-model-results/402_masked_1146086_3.jpg]

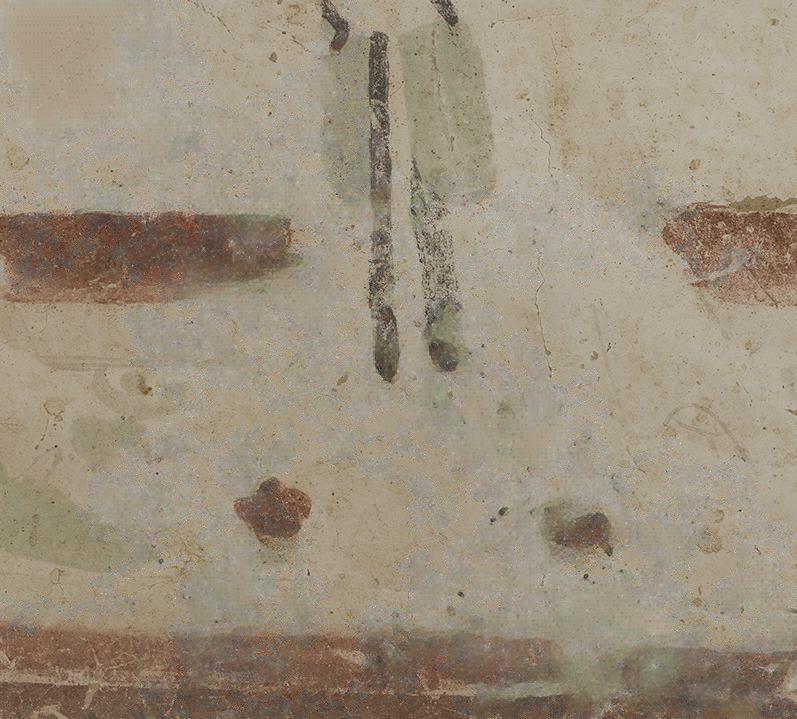

Supplement: Supplementary file 1 [file sensors-21-02091-s001.zip › smartsensors_supplementary_data/nazerietal-model-results/402_masked_2005650_0.jpg]

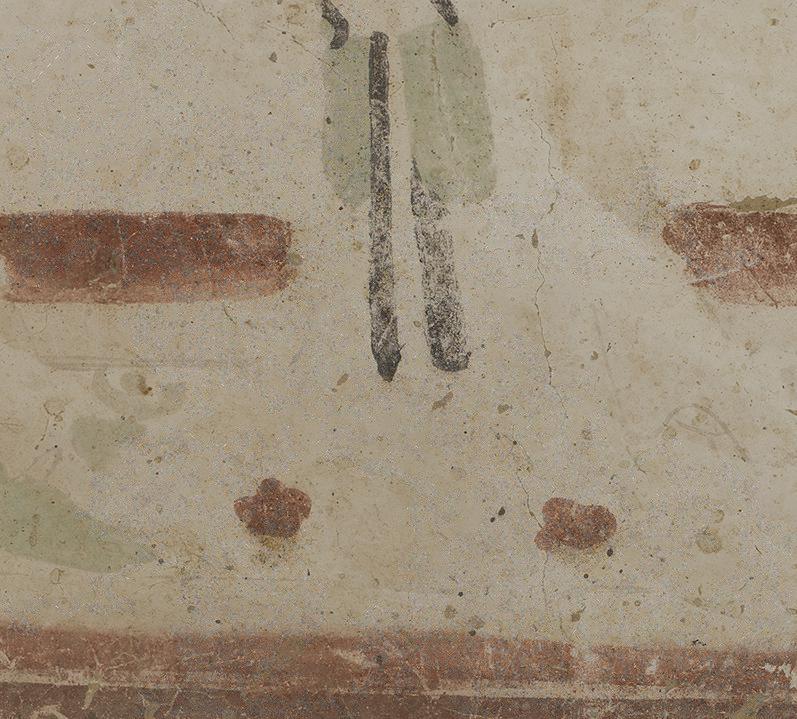

Supplement: Supplementary file 1 [file sensors-21-02091-s001.zip › smartsensors_supplementary_data/nazerietal-model-results/402_masked_2005650_1.jpg]

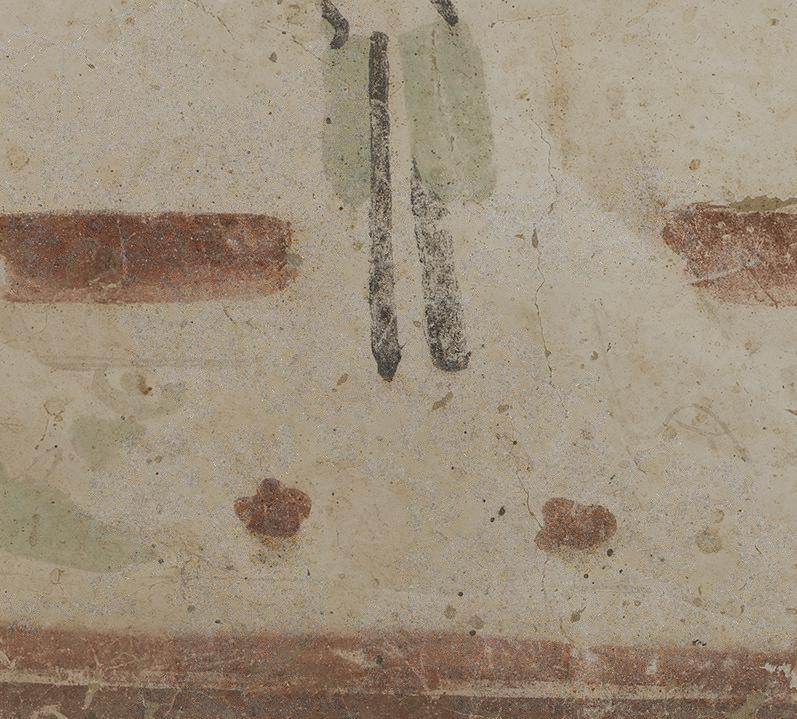

Supplement: Supplementary file 1 [file sensors-21-02091-s001.zip › smartsensors_supplementary_data/nazerietal-model-results/402_masked_2005650_2.jpg]

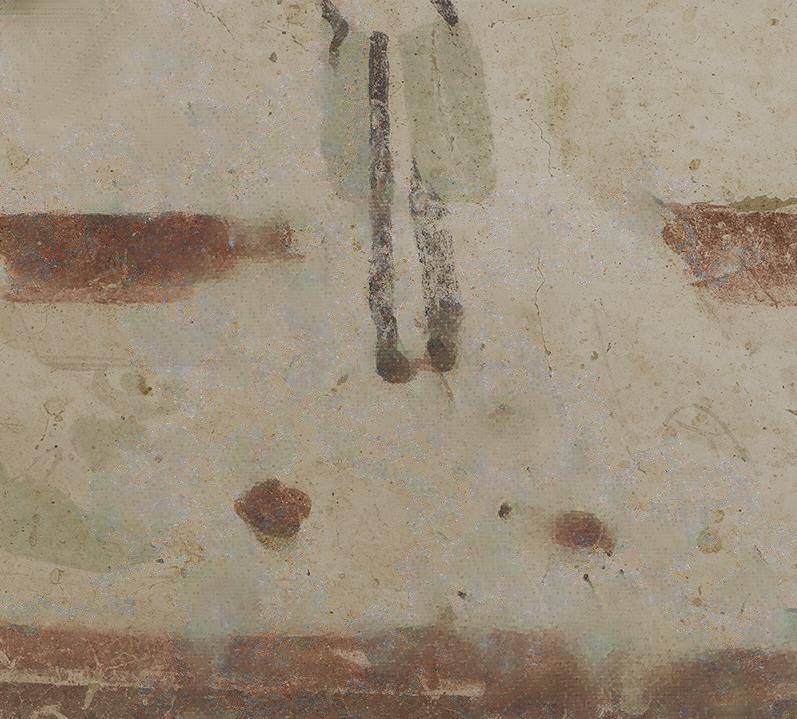

Supplement: Supplementary file 1 [file sensors-21-02091-s001.zip › smartsensors_supplementary_data/nazerietal-model-results/402_masked_2005650_3.jpg]

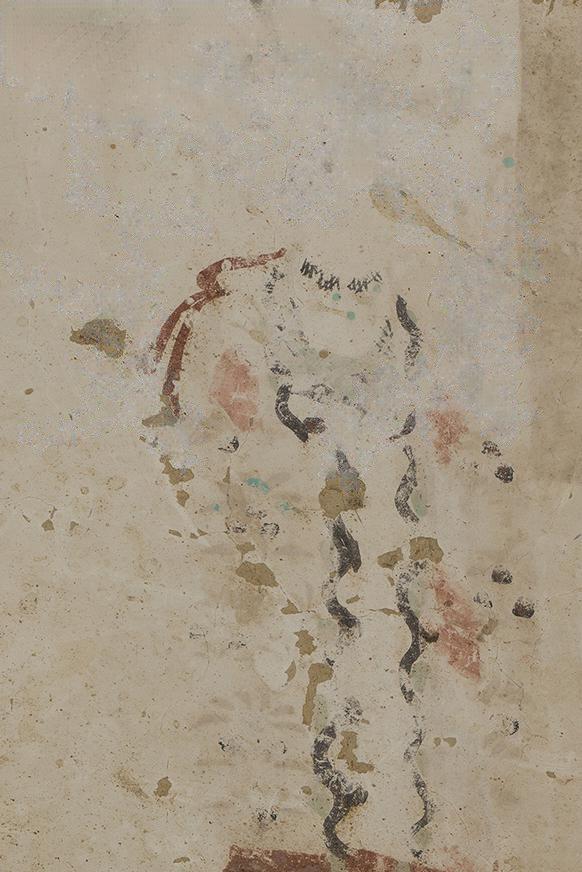

Supplement: Supplementary file 1 [file sensors-21-02091-s001.zip › smartsensors_supplementary_data/nazerietal-model-results/493_masked_1015008_0.jpg]

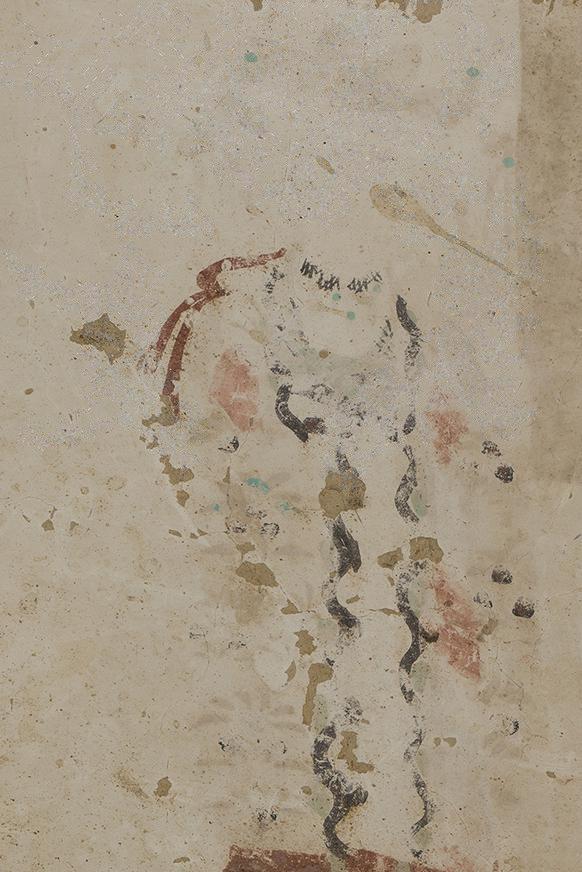

Supplement: Supplementary file 1 [file sensors-21-02091-s001.zip › smartsensors_supplementary_data/nazerietal-model-results/493_masked_1015008_1.jpg]

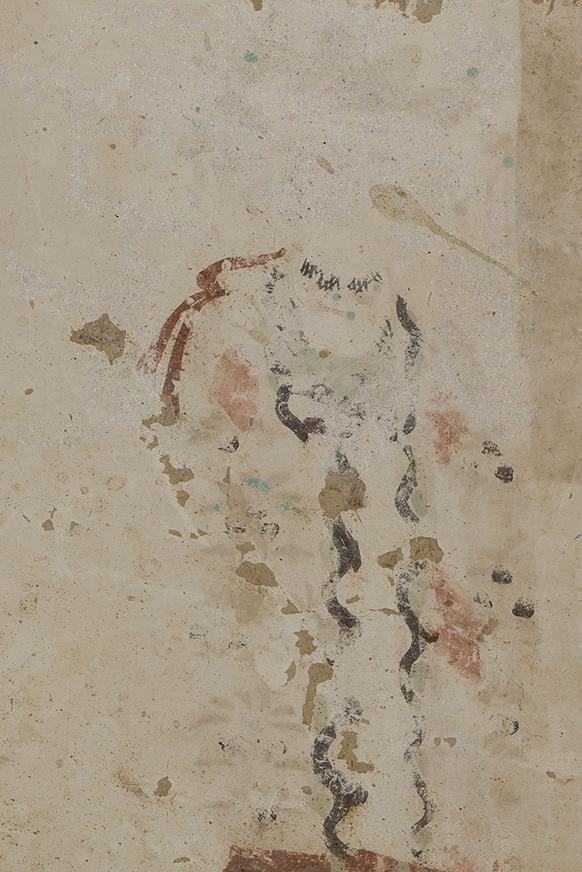

Supplement: Supplementary file 1 [file sensors-21-02091-s001.zip › smartsensors_supplementary_data/nazerietal-model-results/493_masked_1015008_2.jpg]

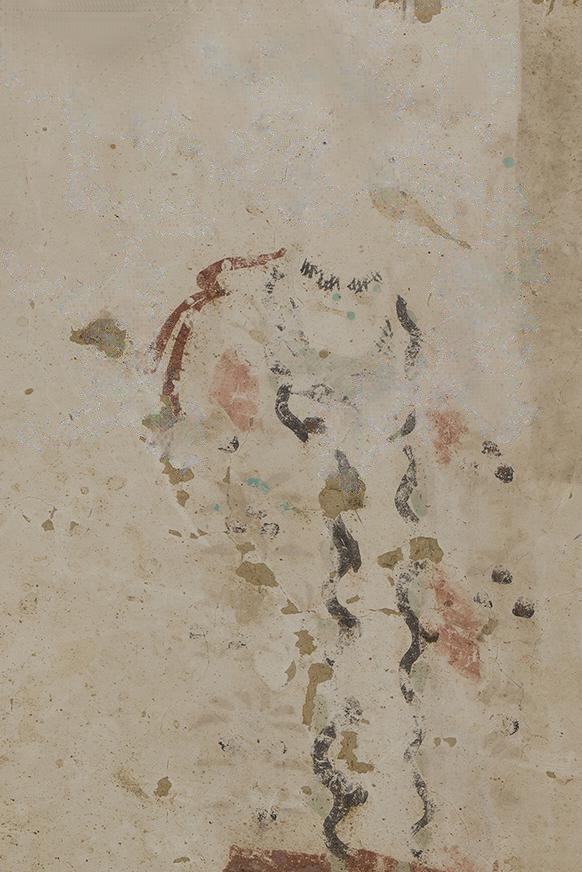

Supplement: Supplementary file 1 [file sensors-21-02091-s001.zip › smartsensors_supplementary_data/nazerietal-model-results/493_masked_1015008_3.jpg]

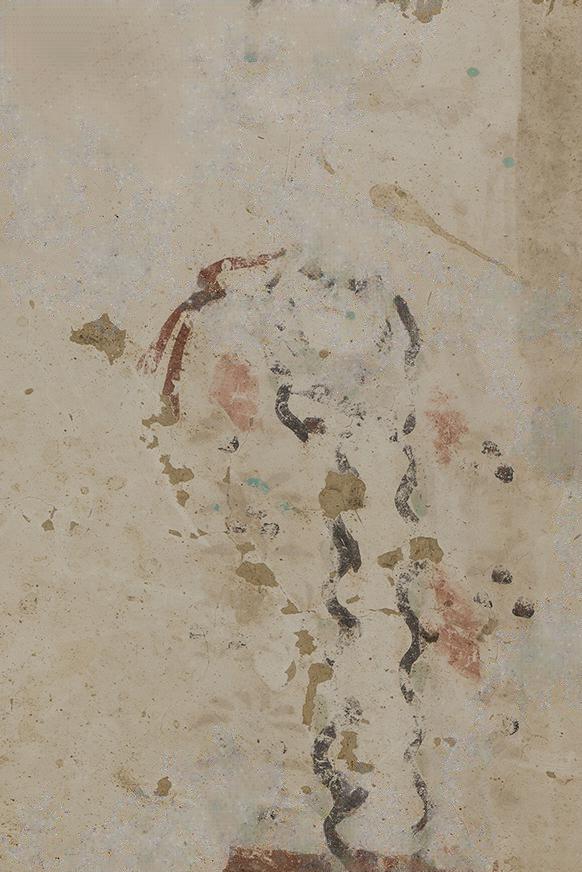

Supplement: Supplementary file 1 [file sensors-21-02091-s001.zip › smartsensors_supplementary_data/nazerietal-model-results/493_masked_1776264_0.jpg]

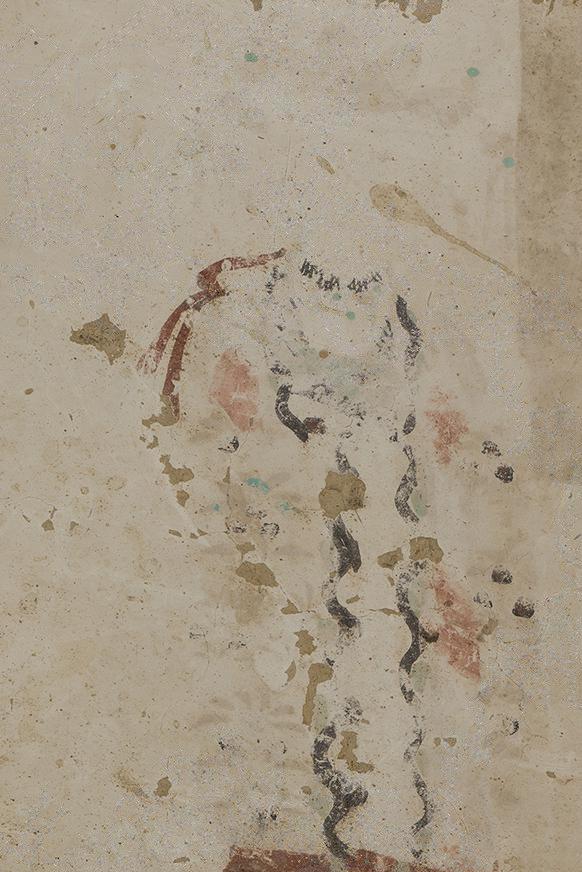

Supplement: Supplementary file 1 [file sensors-21-02091-s001.zip › smartsensors_supplementary_data/nazerietal-model-results/493_masked_1776264_1.jpg]

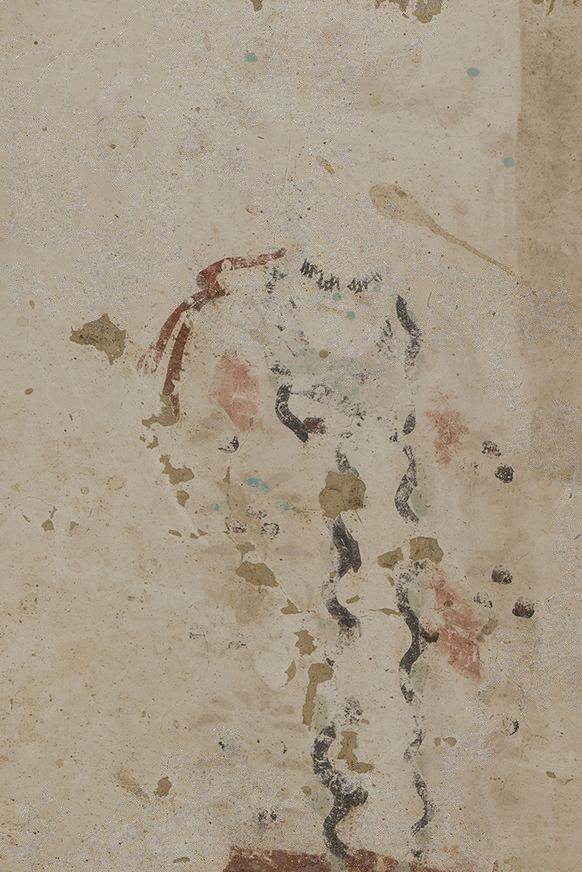

Supplement: Supplementary file 1 [file sensors-21-02091-s001.zip › smartsensors_supplementary_data/nazerietal-model-results/493_masked_1776264_2.jpg]
