# Supplementary figures and images for: Colour-Balanced Edge-Guided Digital Inpainting: Applications on Artworks (part 1 of 2)
Source: Sensors (Basel). 2021 Mar 17;21(6):2091. doi: 10.3390/s21062091 (PMC8002538; doi:10.3390/s21062091)

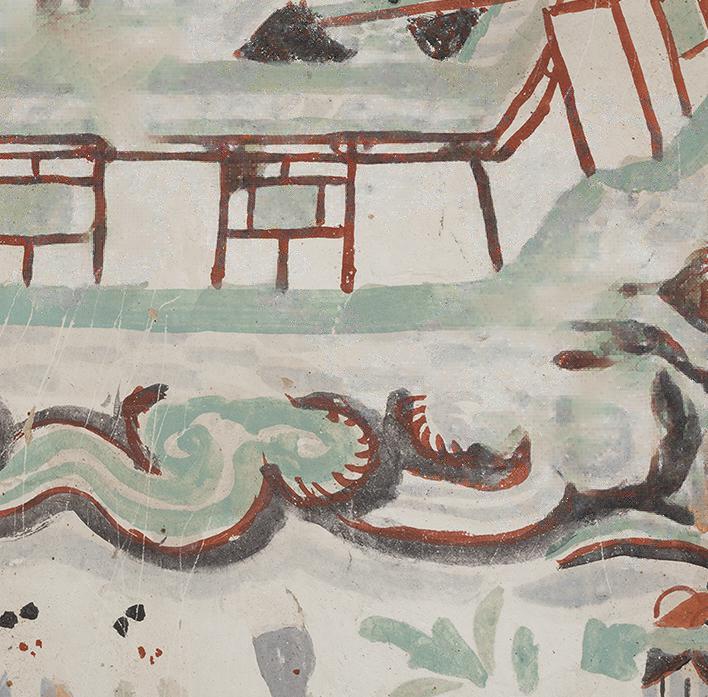

Supplement: Supplementary file 1 [file sensors-21-02091-s001.zip › smartsensors_supplementary_data/nazerietal-model-results/010_masked_1727166_0.jpg]

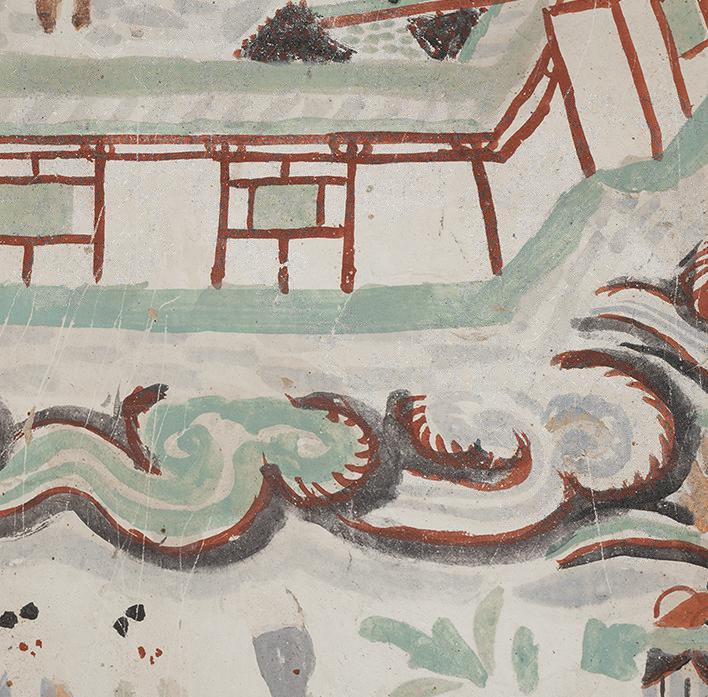

Supplement: Supplementary file 1 [file sensors-21-02091-s001.zip › smartsensors_supplementary_data/nazerietal-model-results/010_masked_1727166_1.jpg]

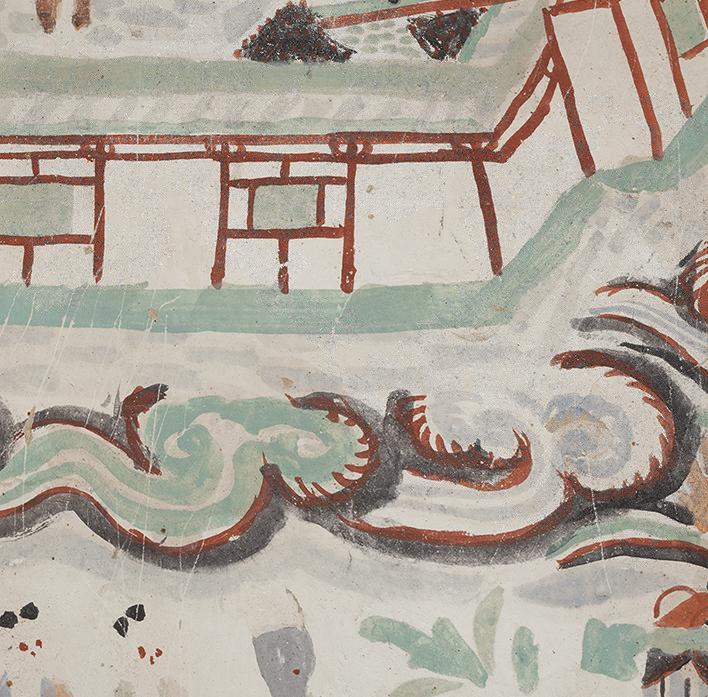

Supplement: Supplementary file 1 [file sensors-21-02091-s001.zip › smartsensors_supplementary_data/nazerietal-model-results/010_masked_1727166_2.jpg]

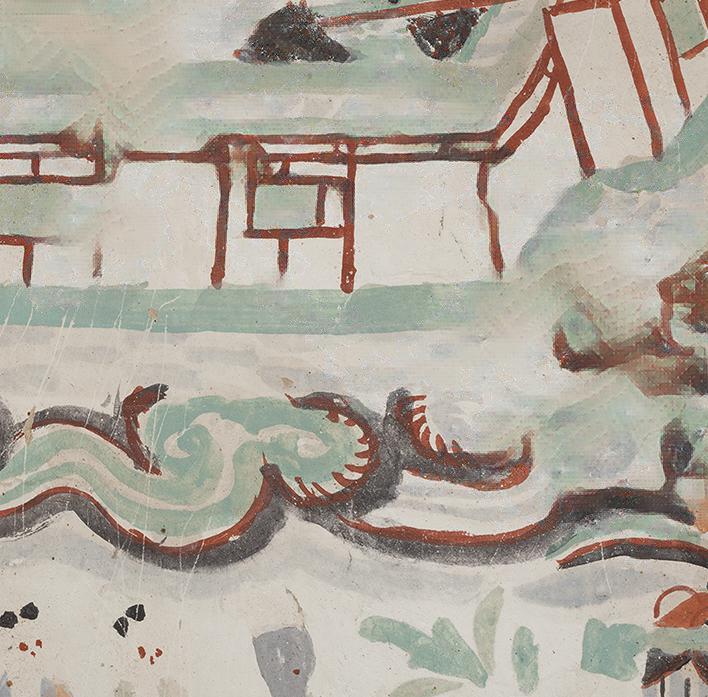

Supplement: Supplementary file 1 [file sensors-21-02091-s001.zip › smartsensors_supplementary_data/nazerietal-model-results/010_masked_1727166_3.jpg]

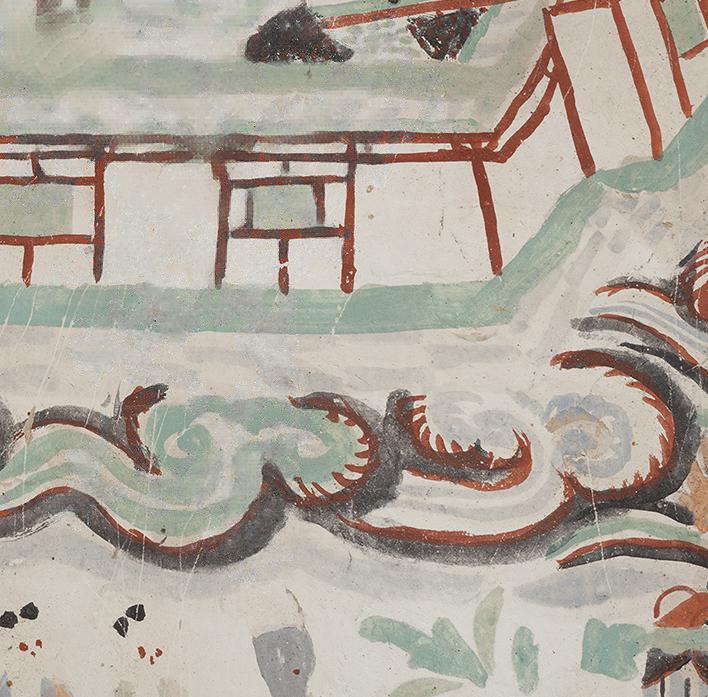

Supplement: Supplementary file 1 [file sensors-21-02091-s001.zip › smartsensors_supplementary_data/nazerietal-model-results/010_masked_986952_0.jpg]

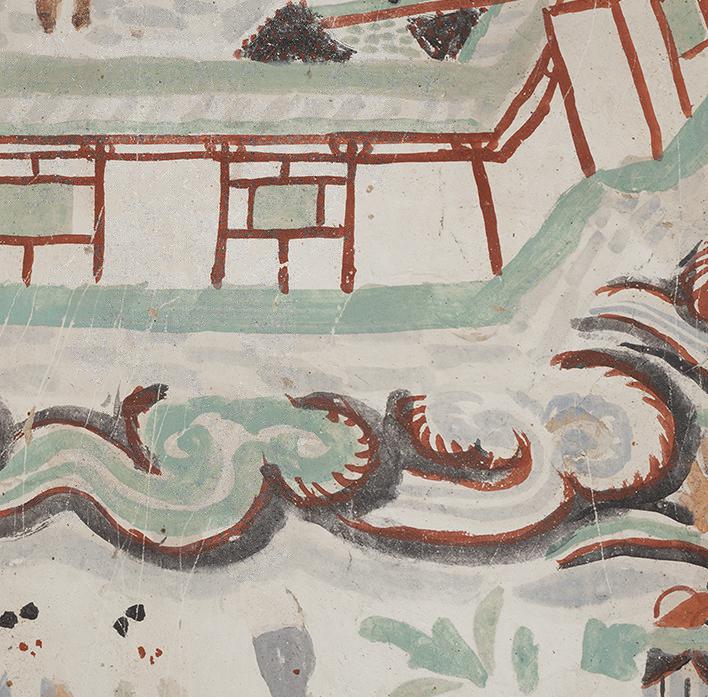

Supplement: Supplementary file 1 [file sensors-21-02091-s001.zip › smartsensors_supplementary_data/nazerietal-model-results/010_masked_986952_1.jpg]

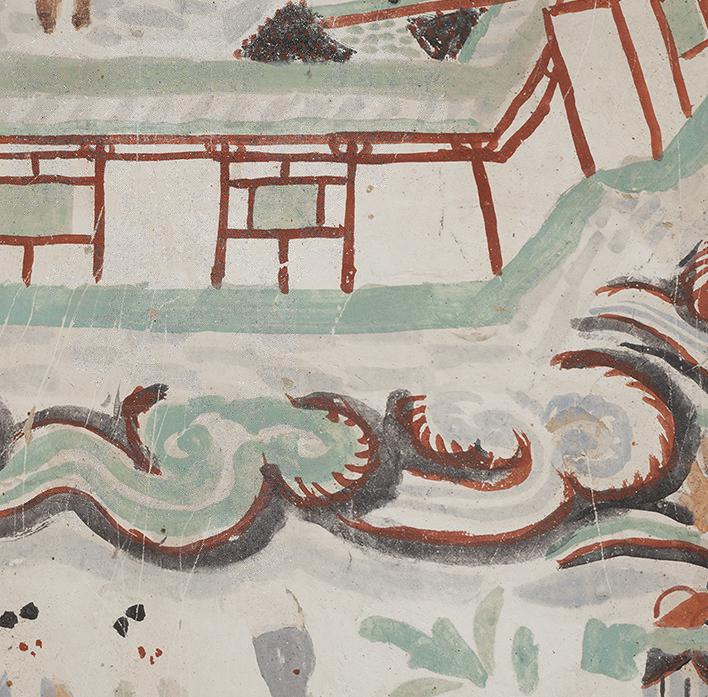

Supplement: Supplementary file 1 [file sensors-21-02091-s001.zip › smartsensors_supplementary_data/nazerietal-model-results/010_masked_986952_2.jpg]

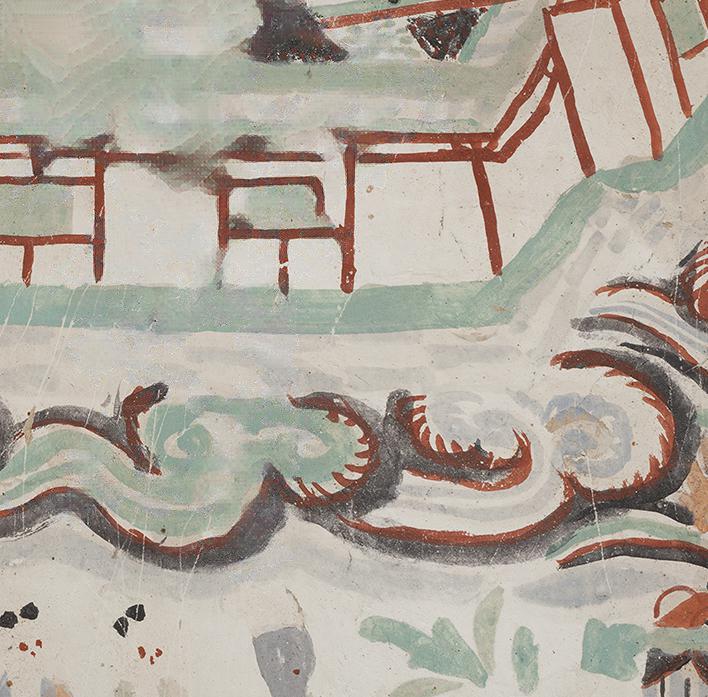

Supplement: Supplementary file 1 [file sensors-21-02091-s001.zip › smartsensors_supplementary_data/nazerietal-model-results/010_masked_986952_3.jpg]

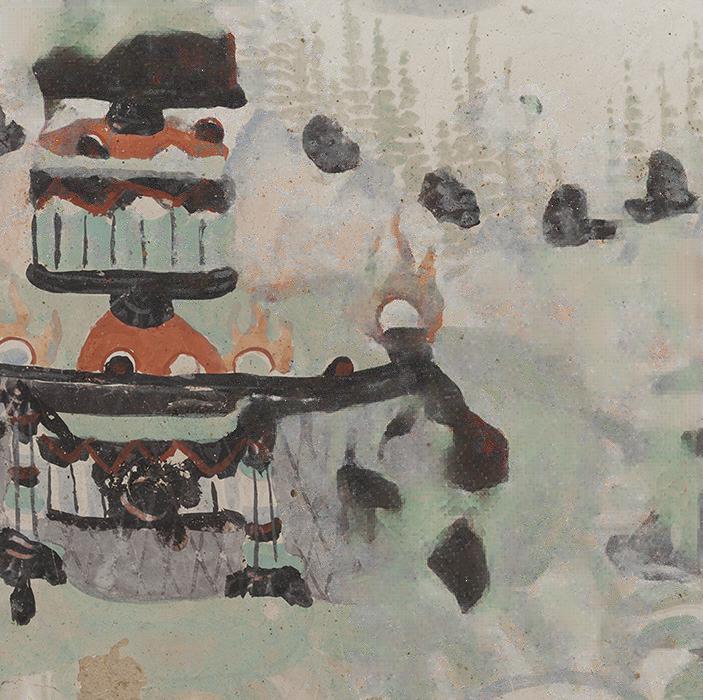

Supplement: Supplementary file 1 [file sensors-21-02091-s001.zip › smartsensors_supplementary_data/nazerietal-model-results/013_masked_1722350_0.jpg]

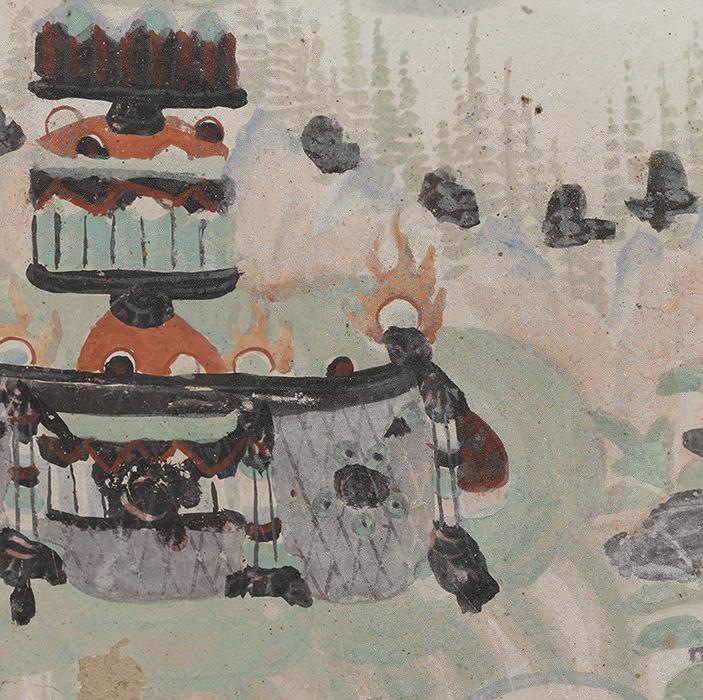

Supplement: Supplementary file 1 [file sensors-21-02091-s001.zip › smartsensors_supplementary_data/nazerietal-model-results/013_masked_1722350_1.jpg]

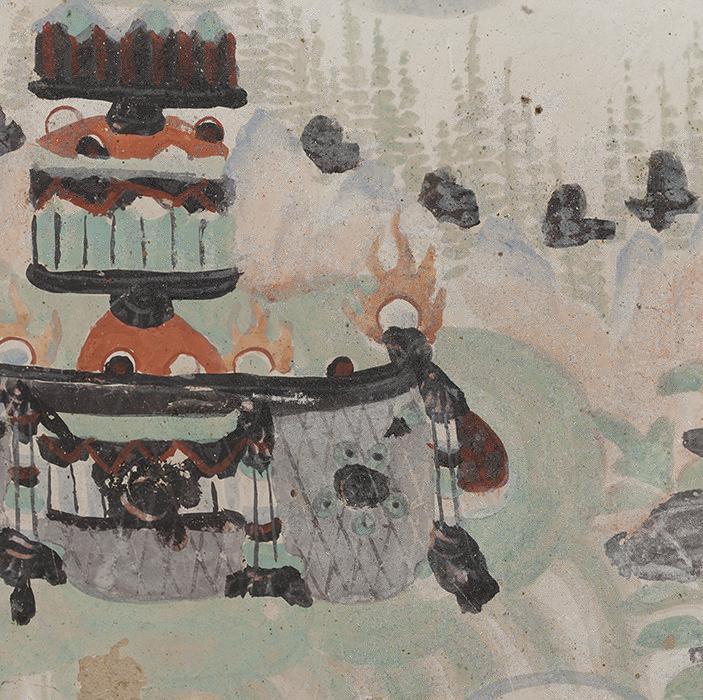

Supplement: Supplementary file 1 [file sensors-21-02091-s001.zip › smartsensors_supplementary_data/nazerietal-model-results/013_masked_1722350_2.jpg]

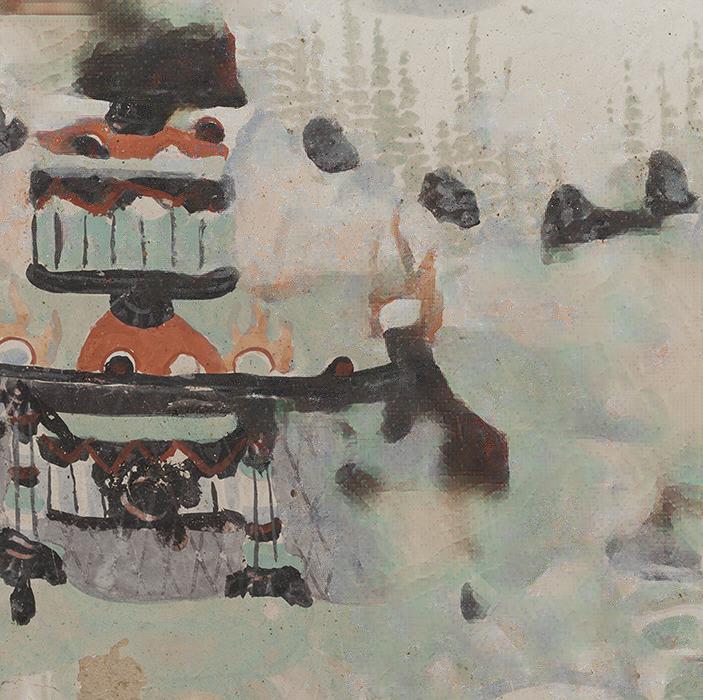

Supplement: Supplementary file 1 [file sensors-21-02091-s001.zip › smartsensors_supplementary_data/nazerietal-model-results/013_masked_1722350_3.jpg]

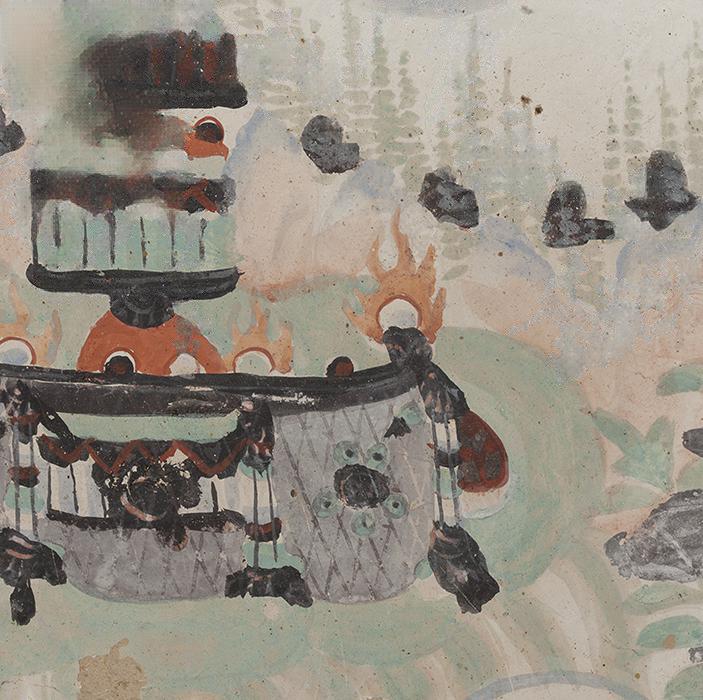

Supplement: Supplementary file 1 [file sensors-21-02091-s001.zip › smartsensors_supplementary_data/nazerietal-model-results/013_masked_984200_0.jpg]

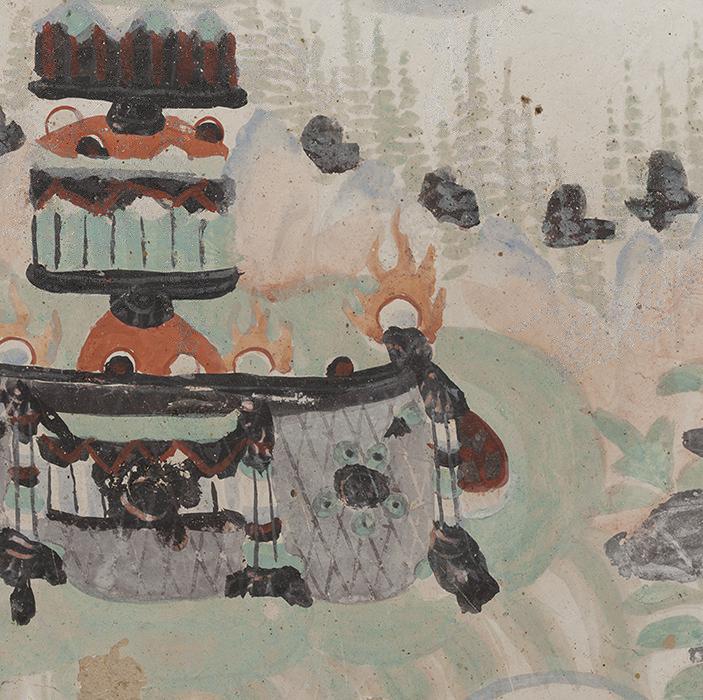

Supplement: Supplementary file 1 [file sensors-21-02091-s001.zip › smartsensors_supplementary_data/nazerietal-model-results/013_masked_984200_1.jpg]

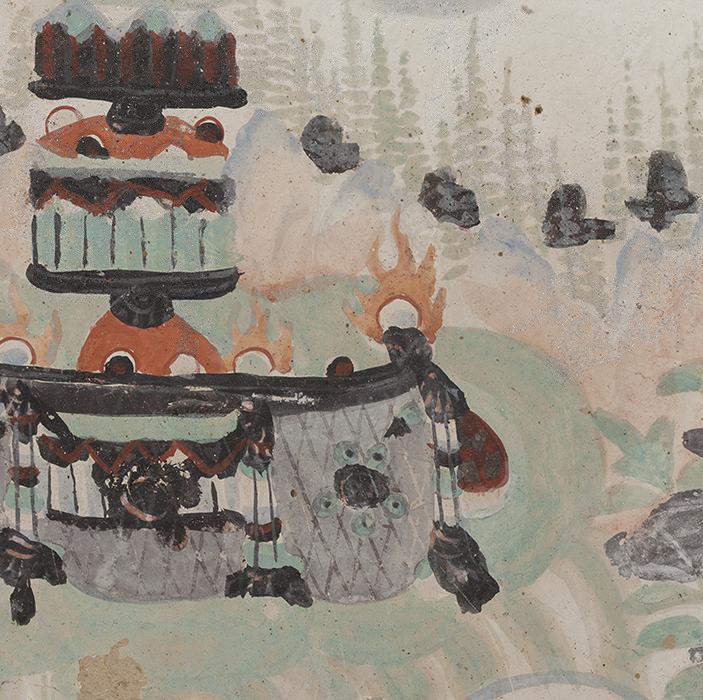

Supplement: Supplementary file 1 [file sensors-21-02091-s001.zip › smartsensors_supplementary_data/nazerietal-model-results/013_masked_984200_2.jpg]

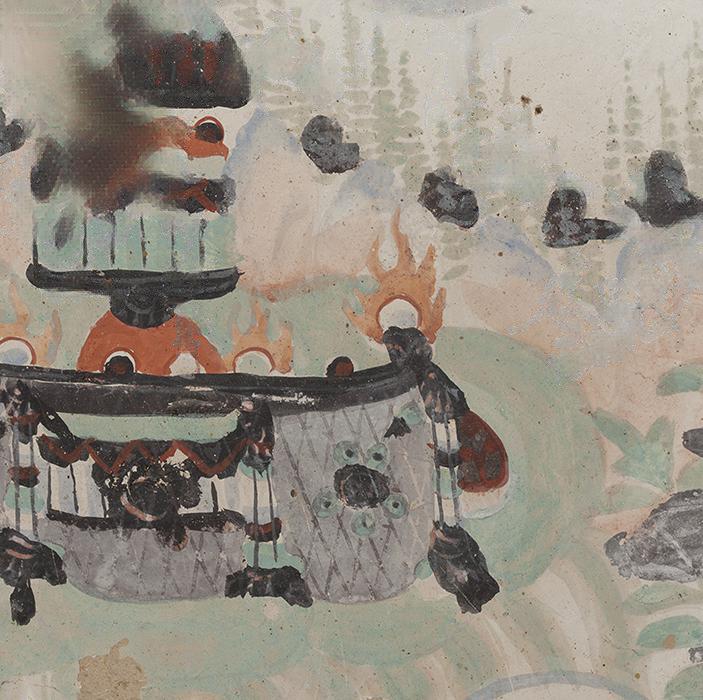

Supplement: Supplementary file 1 [file sensors-21-02091-s001.zip › smartsensors_supplementary_data/nazerietal-model-results/013_masked_984200_3.jpg]

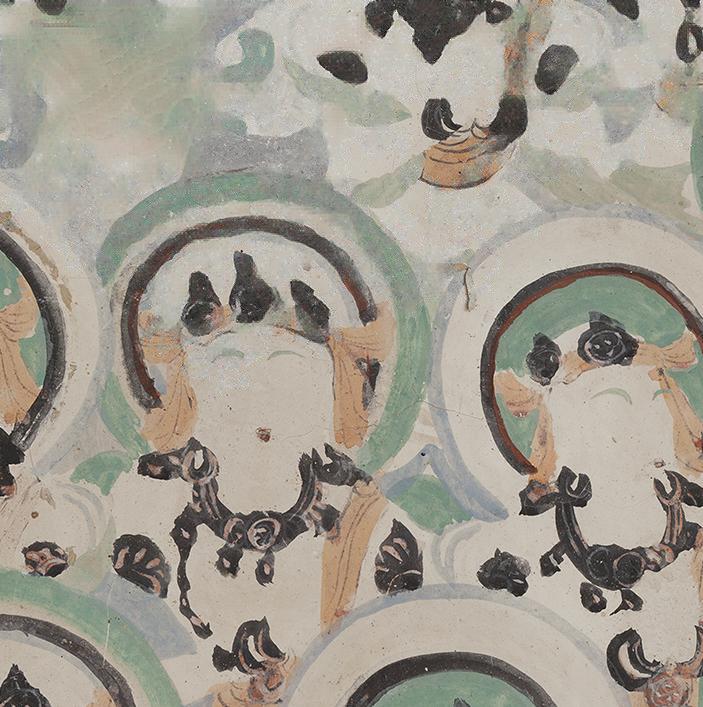

Supplement: Supplementary file 1 [file sensors-21-02091-s001.zip › smartsensors_supplementary_data/nazerietal-model-results/027_masked_1739573_0.jpg]

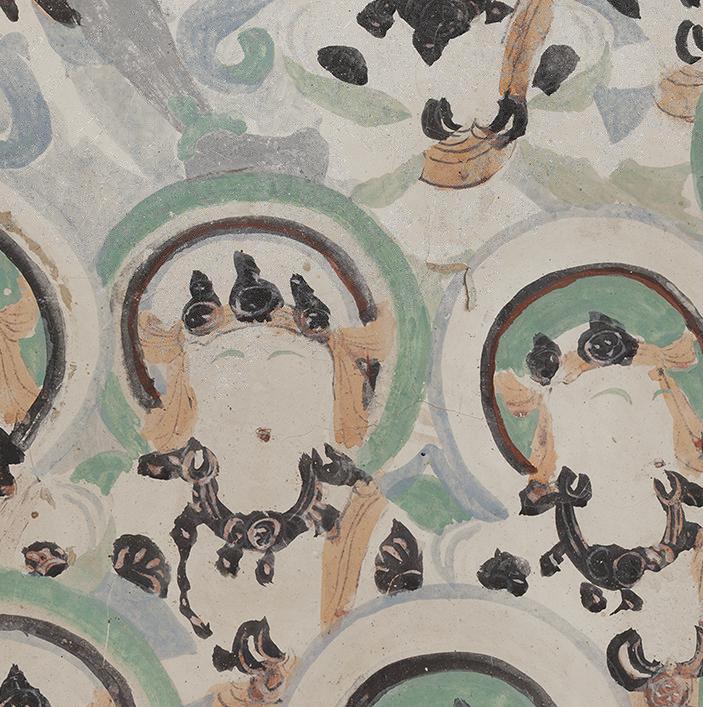

Supplement: Supplementary file 1 [file sensors-21-02091-s001.zip › smartsensors_supplementary_data/nazerietal-model-results/027_masked_1739573_1.jpg]

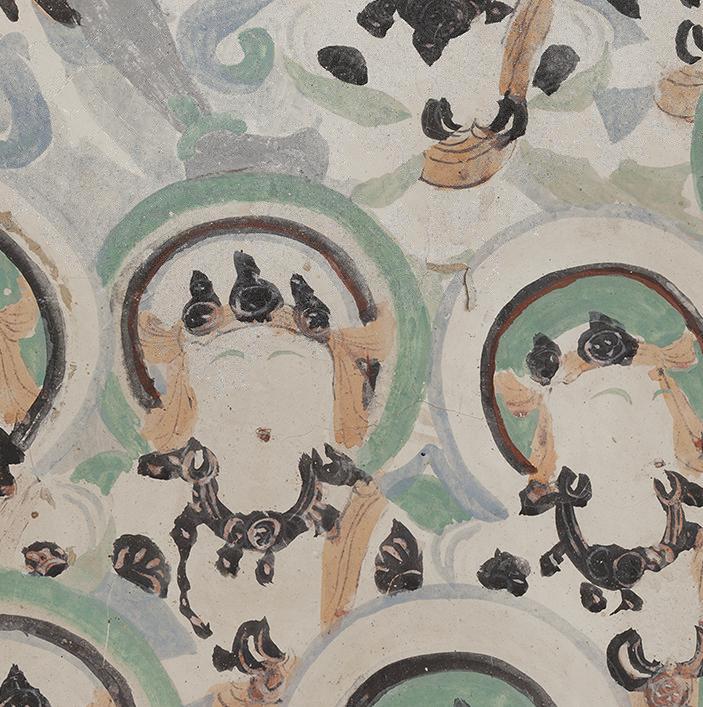

Supplement: Supplementary file 1 [file sensors-21-02091-s001.zip › smartsensors_supplementary_data/nazerietal-model-results/027_masked_1739573_2.jpg]

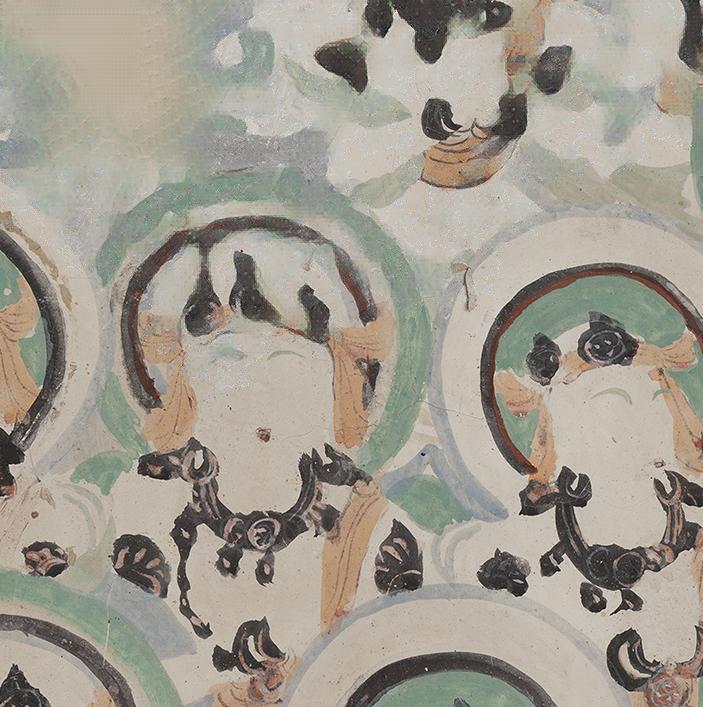

Supplement: Supplementary file 1 [file sensors-21-02091-s001.zip › smartsensors_supplementary_data/nazerietal-model-results/027_masked_1739573_3.jpg]

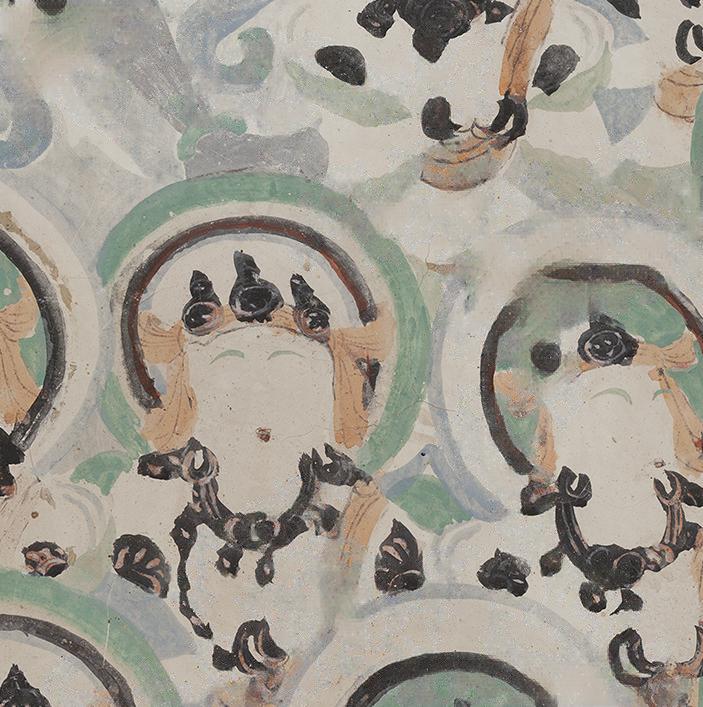

Supplement: Supplementary file 1 [file sensors-21-02091-s001.zip › smartsensors_supplementary_data/nazerietal-model-results/027_masked_994042_0.jpg]

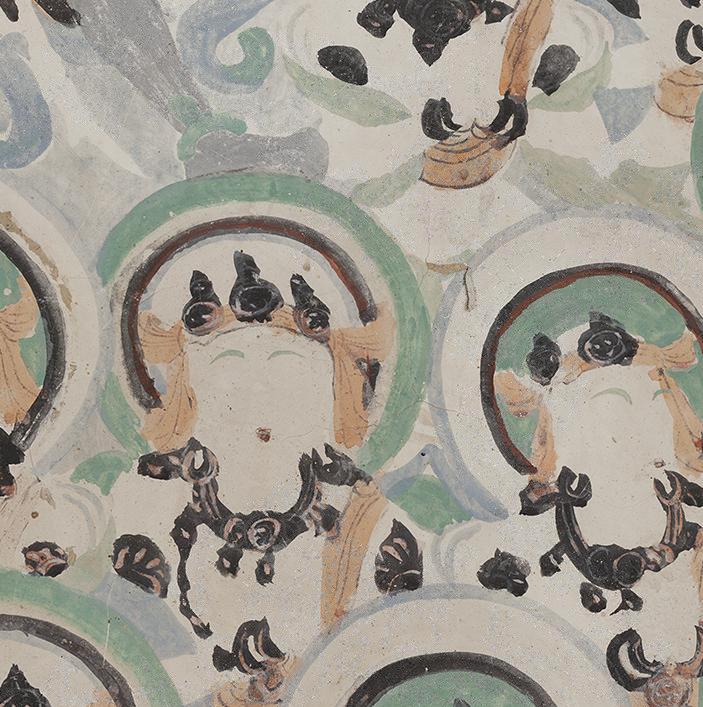

Supplement: Supplementary file 1 [file sensors-21-02091-s001.zip › smartsensors_supplementary_data/nazerietal-model-results/027_masked_994042_1.jpg]

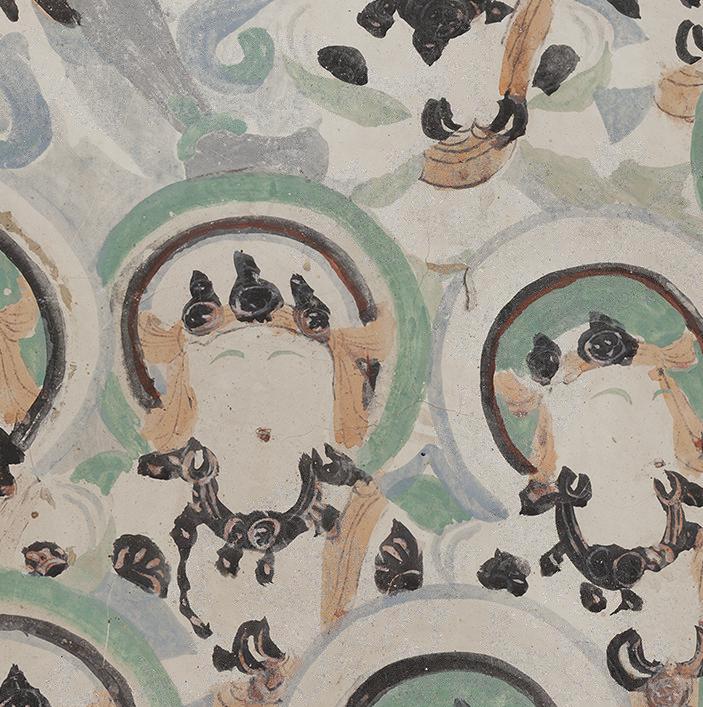

Supplement: Supplementary file 1 [file sensors-21-02091-s001.zip › smartsensors_supplementary_data/nazerietal-model-results/027_masked_994042_2.jpg]

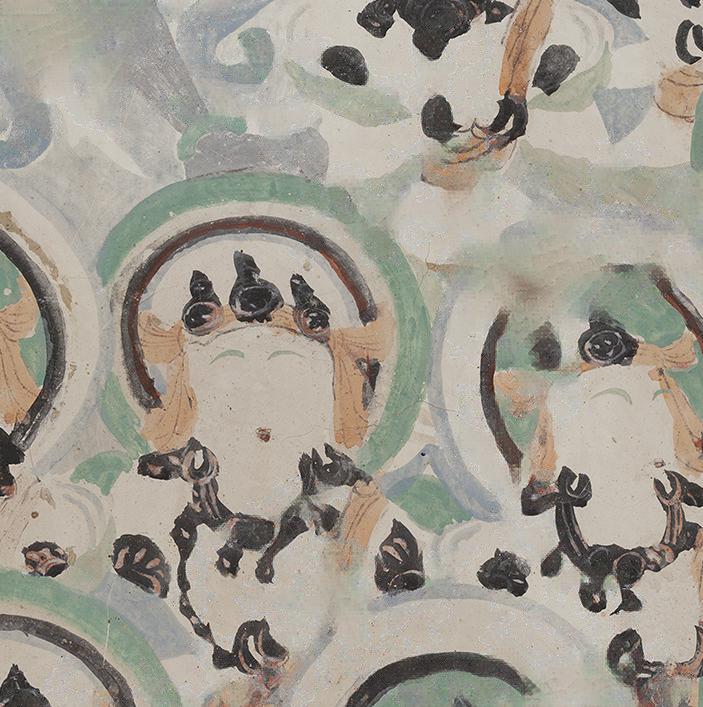

Supplement: Supplementary file 1 [file sensors-21-02091-s001.zip › smartsensors_supplementary_data/nazerietal-model-results/027_masked_994042_3.jpg]

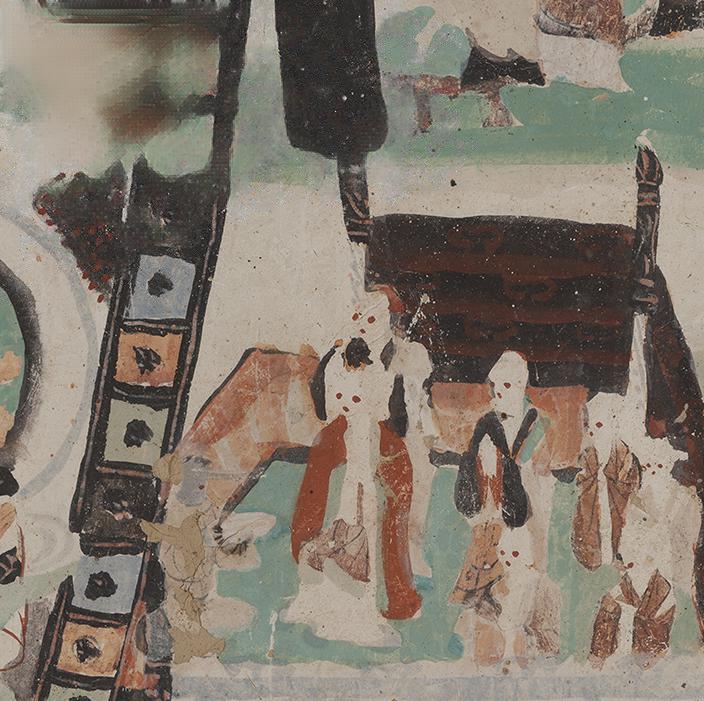

Supplement: Supplementary file 1 [file sensors-21-02091-s001.zip › smartsensors_supplementary_data/nazerietal-model-results/046_masked_1727264_0.jpg]

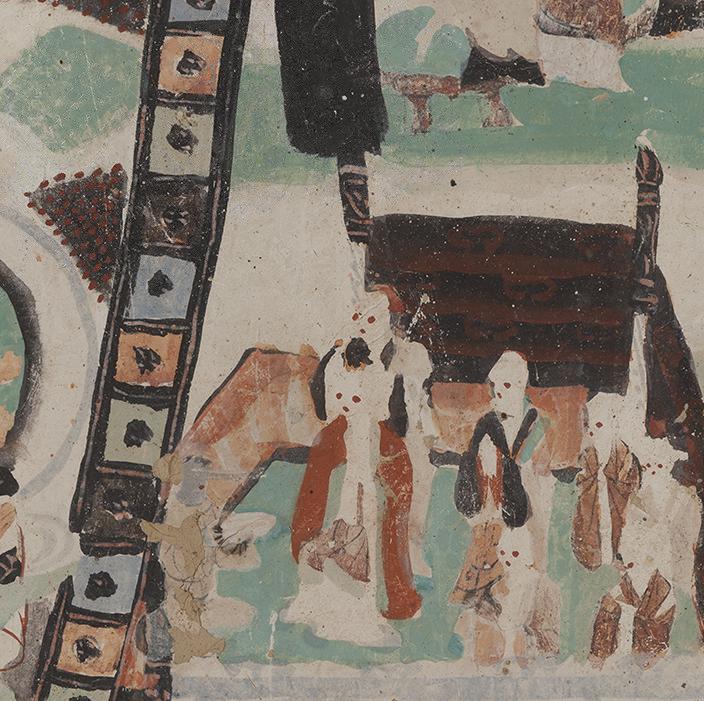

Supplement: Supplementary file 1 [file sensors-21-02091-s001.zip › smartsensors_supplementary_data/nazerietal-model-results/046_masked_1727264_1.jpg]

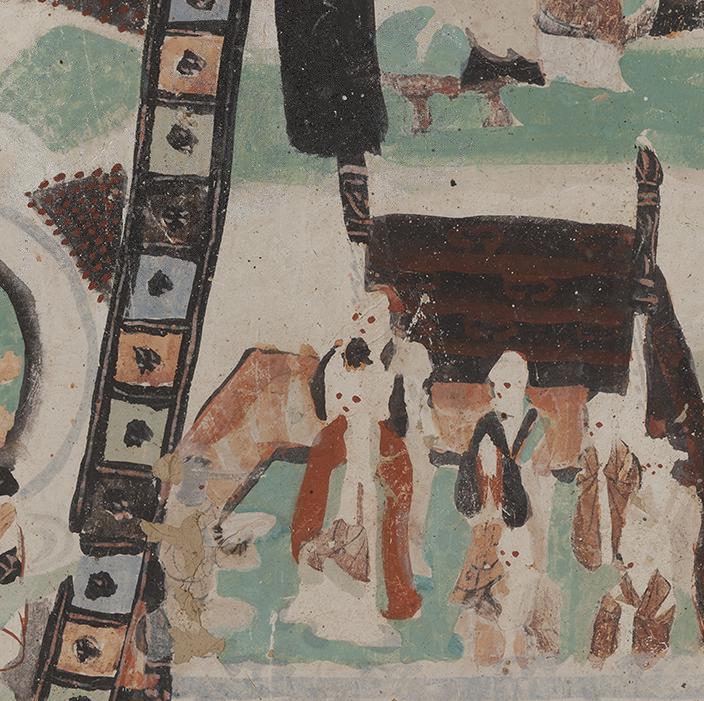

Supplement: Supplementary file 1 [file sensors-21-02091-s001.zip › smartsensors_supplementary_data/nazerietal-model-results/046_masked_1727264_2.jpg]

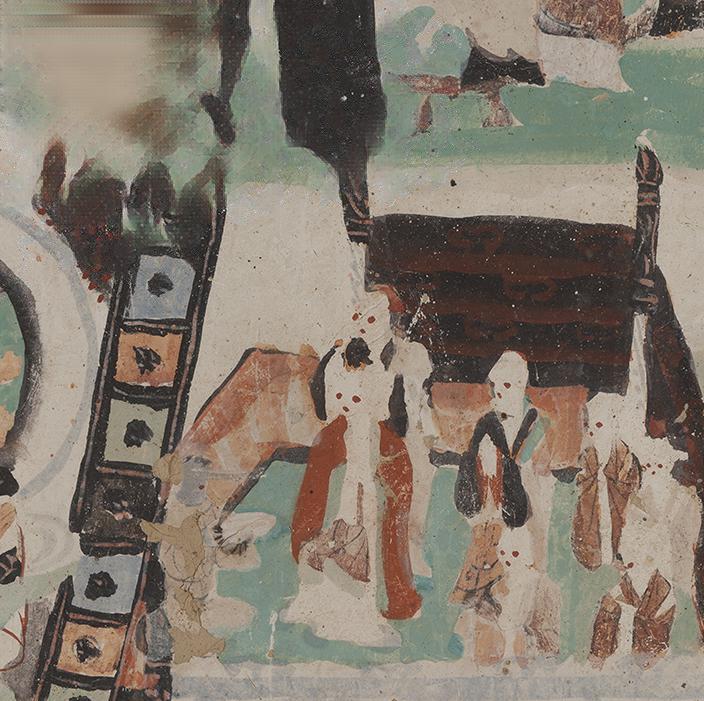

Supplement: Supplementary file 1 [file sensors-21-02091-s001.zip › smartsensors_supplementary_data/nazerietal-model-results/046_masked_1727264_3.jpg]

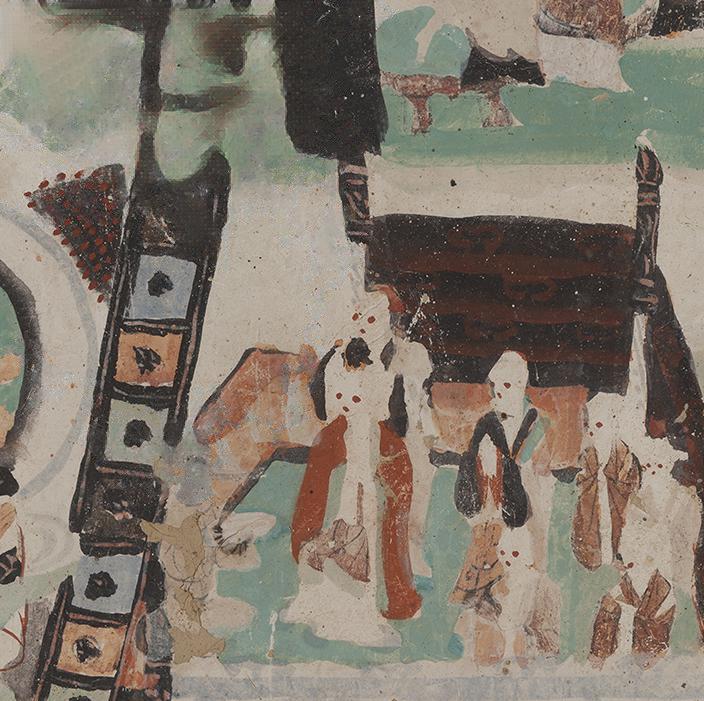

Supplement: Supplementary file 1 [file sensors-21-02091-s001.zip › smartsensors_supplementary_data/nazerietal-model-results/046_masked_987008_0.jpg]

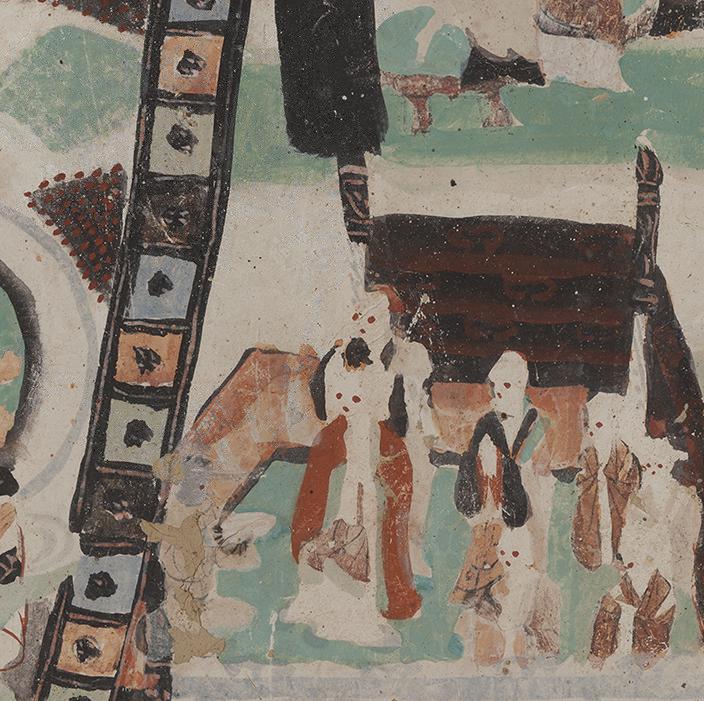

Supplement: Supplementary file 1 [file sensors-21-02091-s001.zip › smartsensors_supplementary_data/nazerietal-model-results/046_masked_987008_1.jpg]

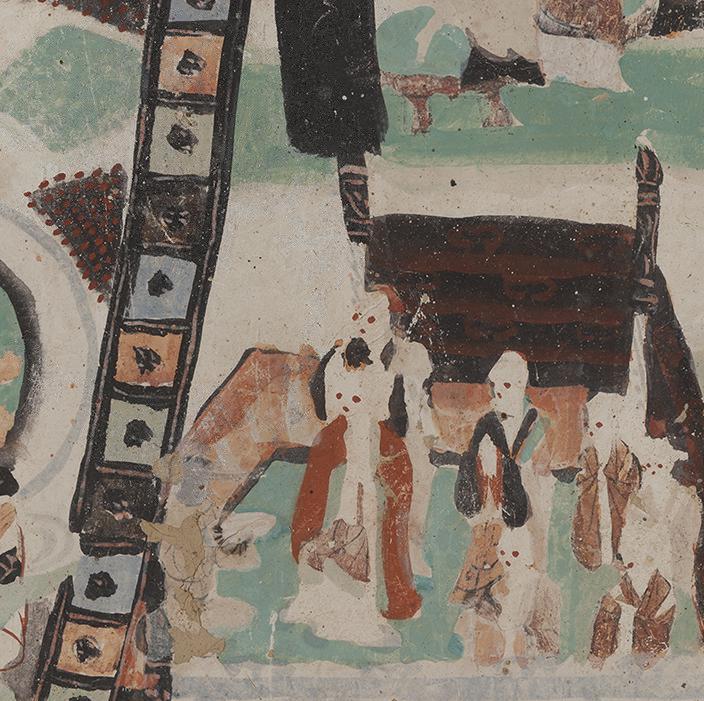

Supplement: Supplementary file 1 [file sensors-21-02091-s001.zip › smartsensors_supplementary_data/nazerietal-model-results/046_masked_987008_2.jpg]

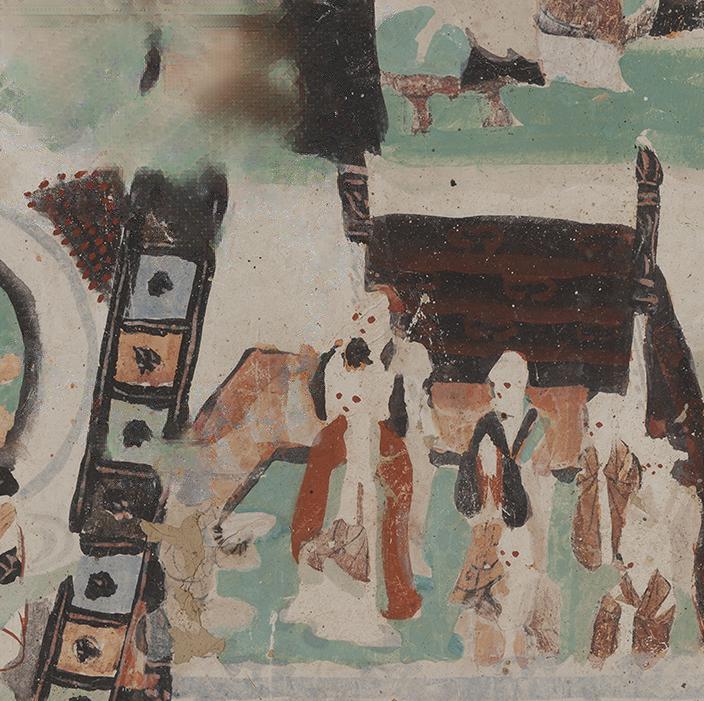

Supplement: Supplementary file 1 [file sensors-21-02091-s001.zip › smartsensors_supplementary_data/nazerietal-model-results/046_masked_987008_3.jpg]

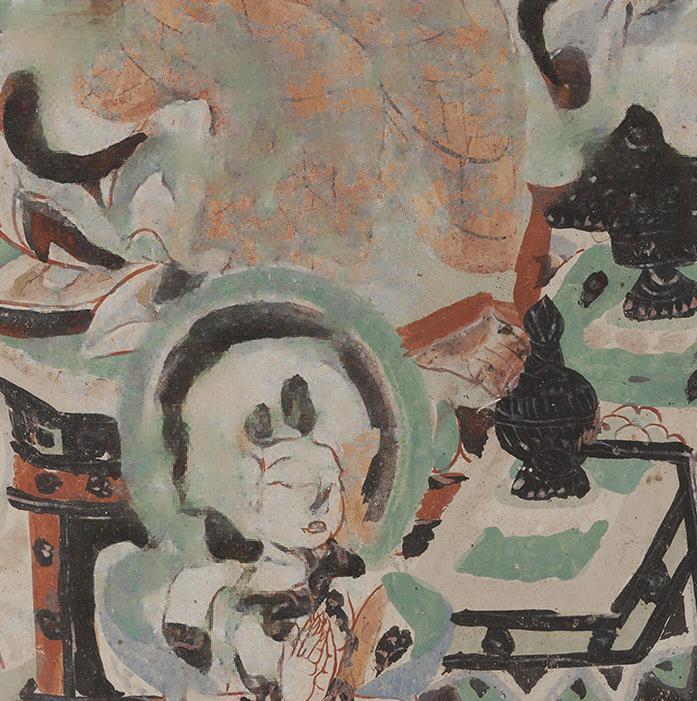

Supplement: Supplementary file 1 [file sensors-21-02091-s001.zip › smartsensors_supplementary_data/nazerietal-model-results/049_masked_1710089_0.jpg]

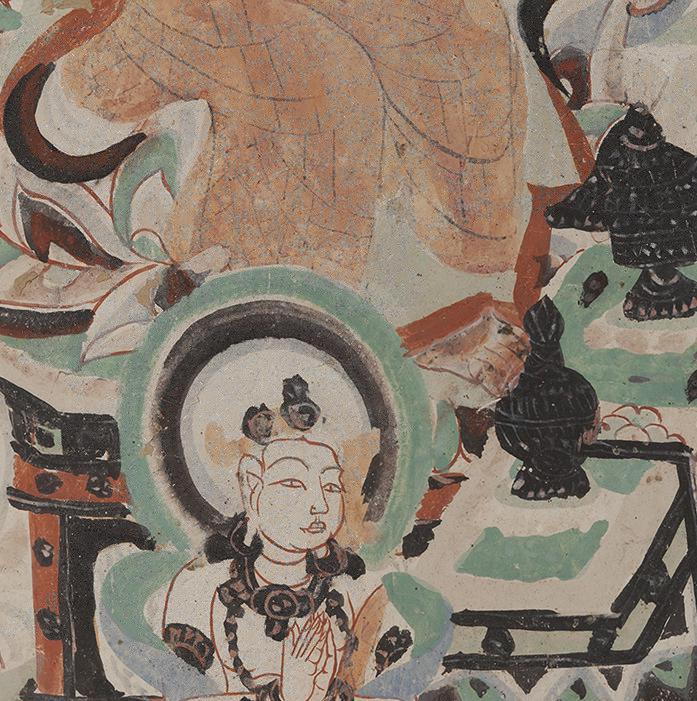

Supplement: Supplementary file 1 [file sensors-21-02091-s001.zip › smartsensors_supplementary_data/nazerietal-model-results/049_masked_1710089_1.jpg]

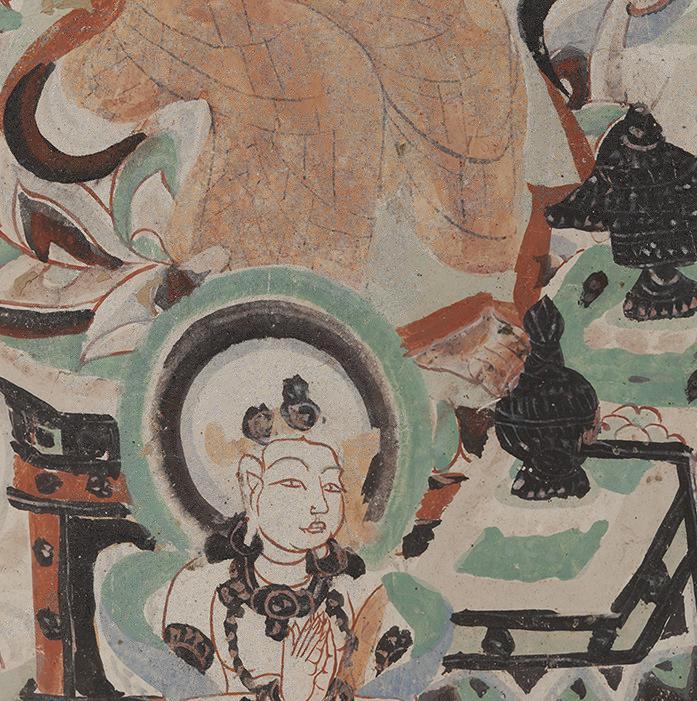

Supplement: Supplementary file 1 [file sensors-21-02091-s001.zip › smartsensors_supplementary_data/nazerietal-model-results/049_masked_1710089_2.jpg]

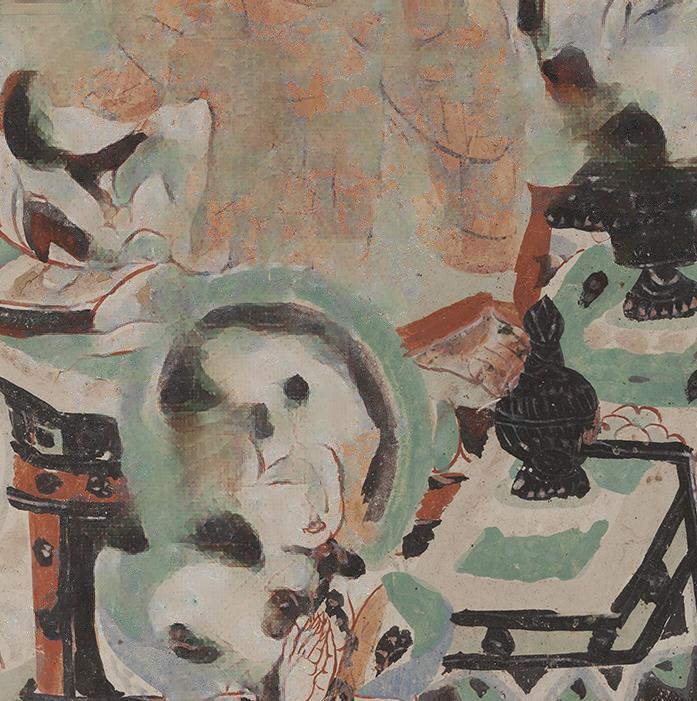

Supplement: Supplementary file 1 [file sensors-21-02091-s001.zip › smartsensors_supplementary_data/nazerietal-model-results/049_masked_1710089_3.jpg]

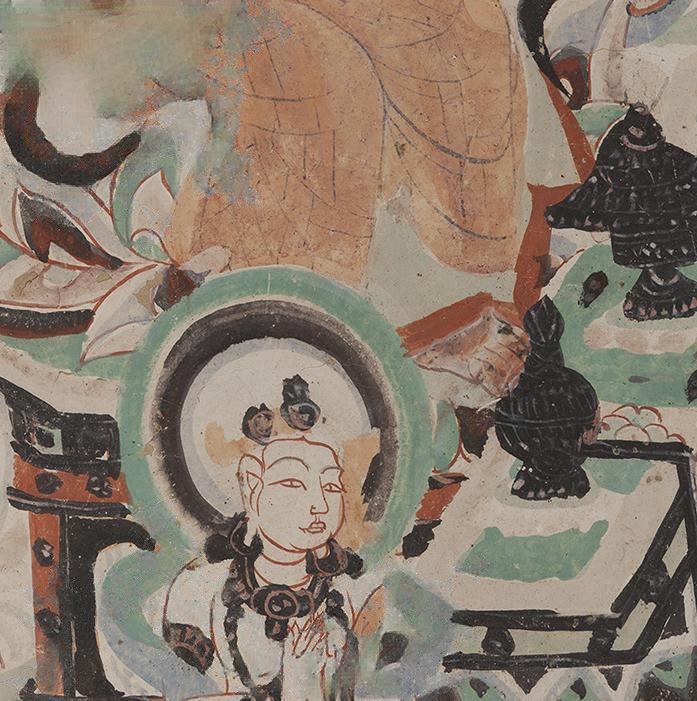

Supplement: Supplementary file 1 [file sensors-21-02091-s001.zip › smartsensors_supplementary_data/nazerietal-model-results/049_masked_977194_0.jpg]

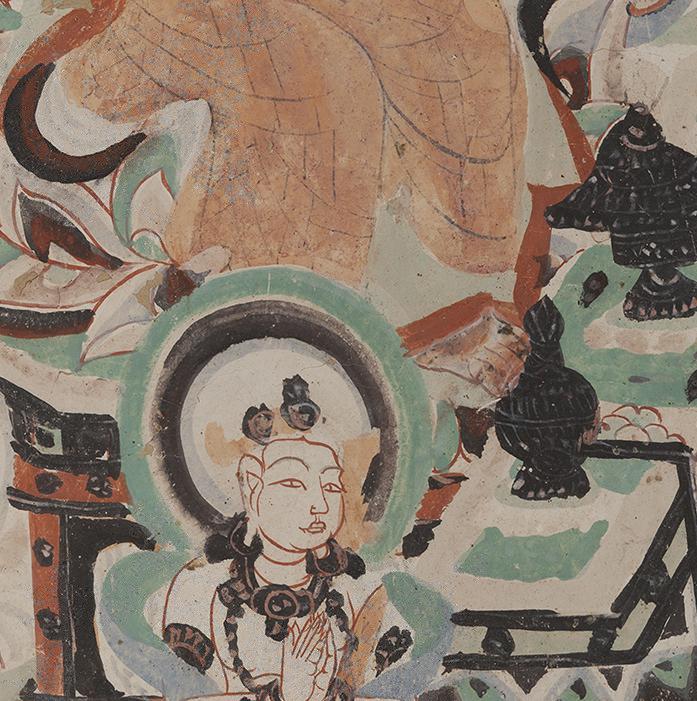

Supplement: Supplementary file 1 [file sensors-21-02091-s001.zip › smartsensors_supplementary_data/nazerietal-model-results/049_masked_977194_1.jpg]

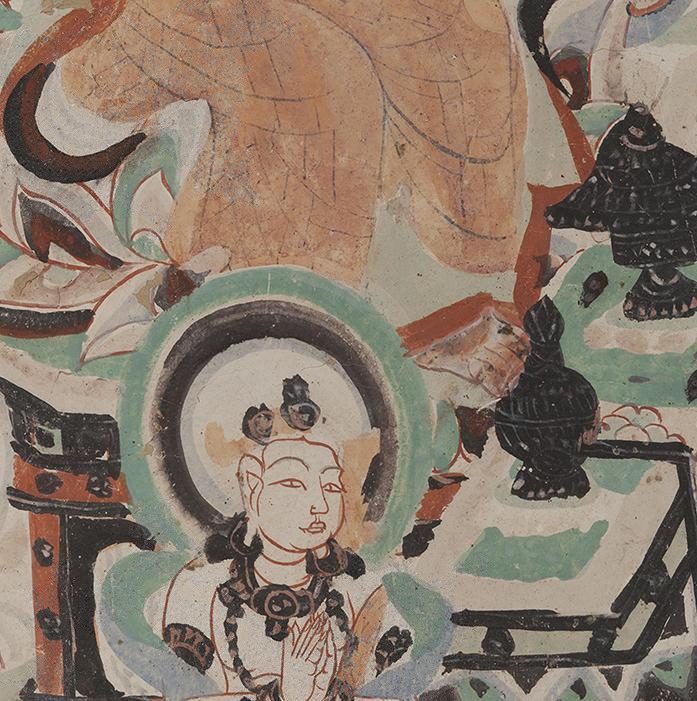

Supplement: Supplementary file 1 [file sensors-21-02091-s001.zip › smartsensors_supplementary_data/nazerietal-model-results/049_masked_977194_2.jpg]

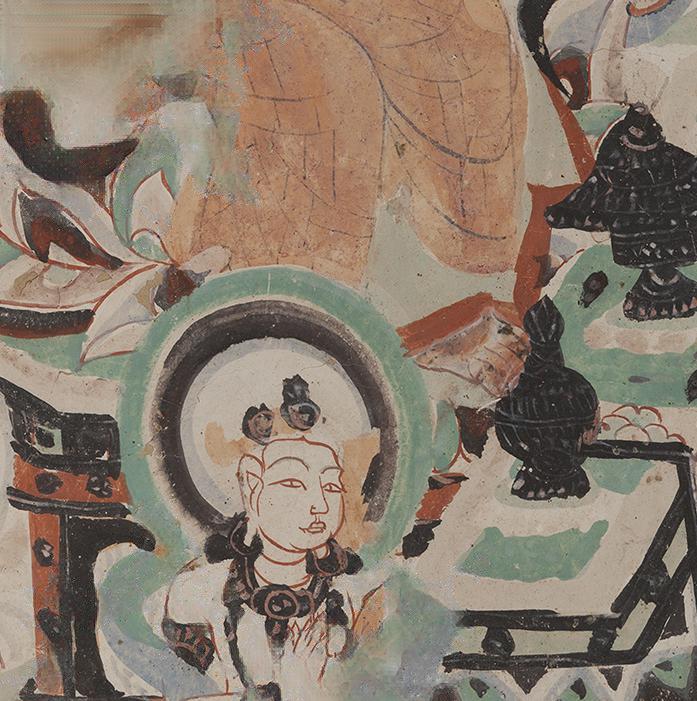

Supplement: Supplementary file 1 [file sensors-21-02091-s001.zip › smartsensors_supplementary_data/nazerietal-model-results/049_masked_977194_3.jpg]

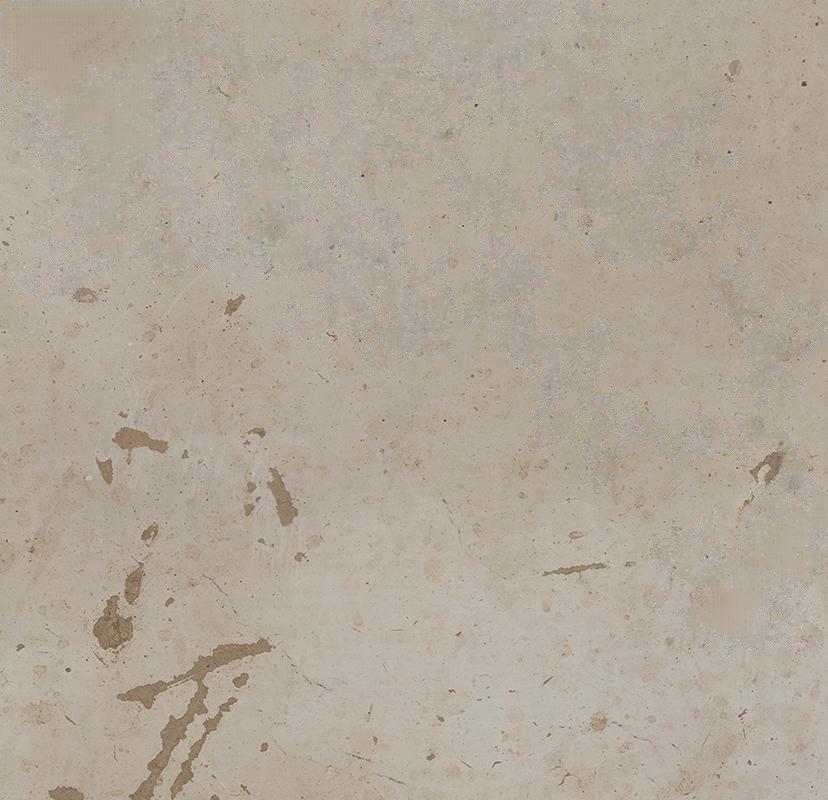

Supplement: Supplementary file 1 [file sensors-21-02091-s001.zip › smartsensors_supplementary_data/nazerietal-model-results/054_masked_1324800_0.jpg]

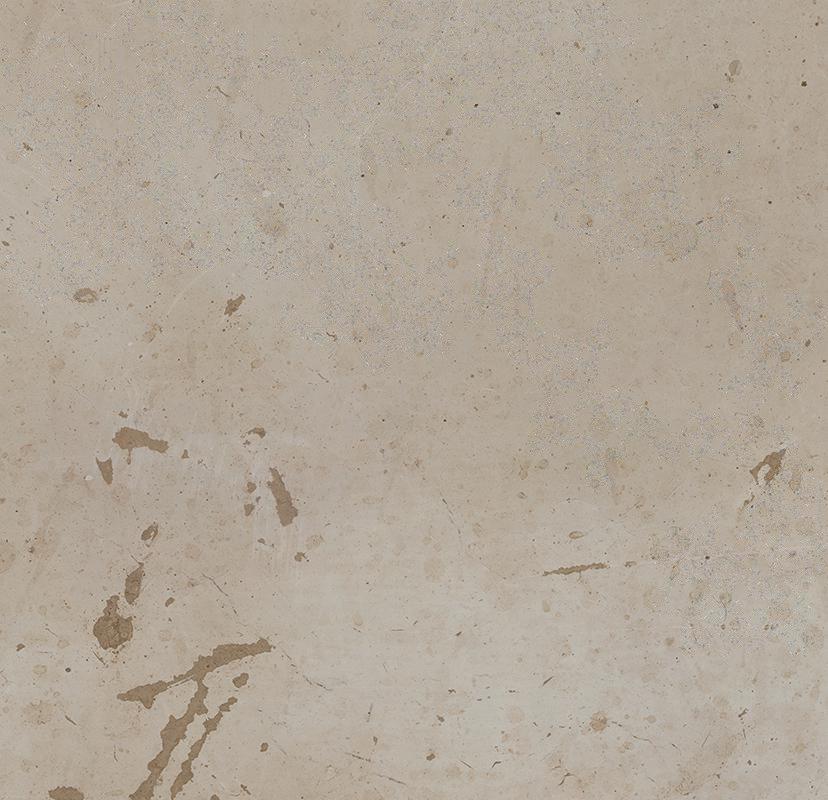

Supplement: Supplementary file 1 [file sensors-21-02091-s001.zip › smartsensors_supplementary_data/nazerietal-model-results/054_masked_1324800_1.jpg]

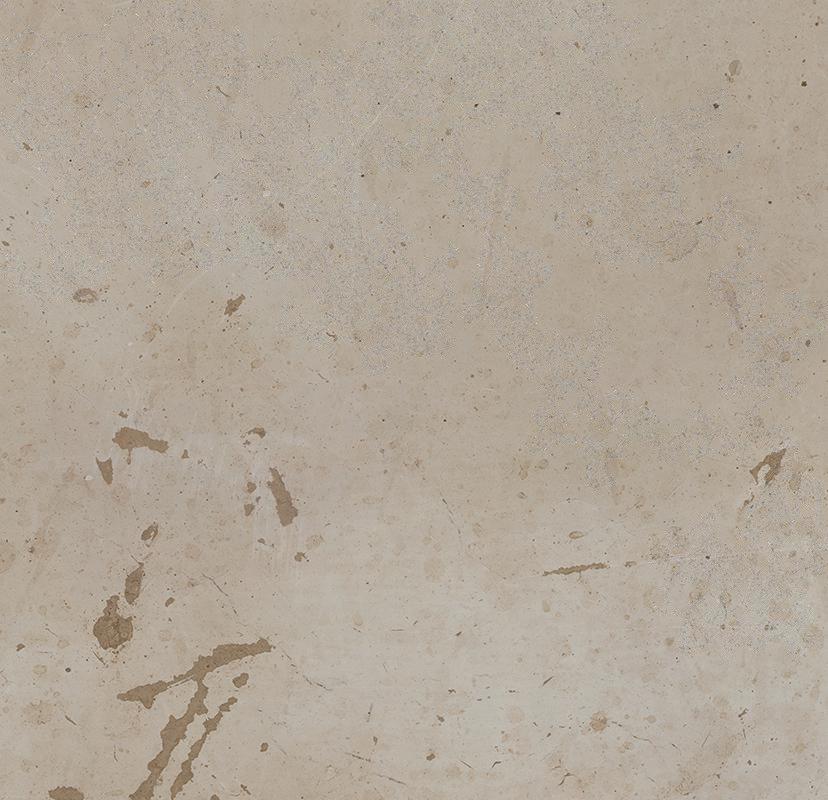

Supplement: Supplementary file 1 [file sensors-21-02091-s001.zip › smartsensors_supplementary_data/nazerietal-model-results/054_masked_1324800_2.jpg]

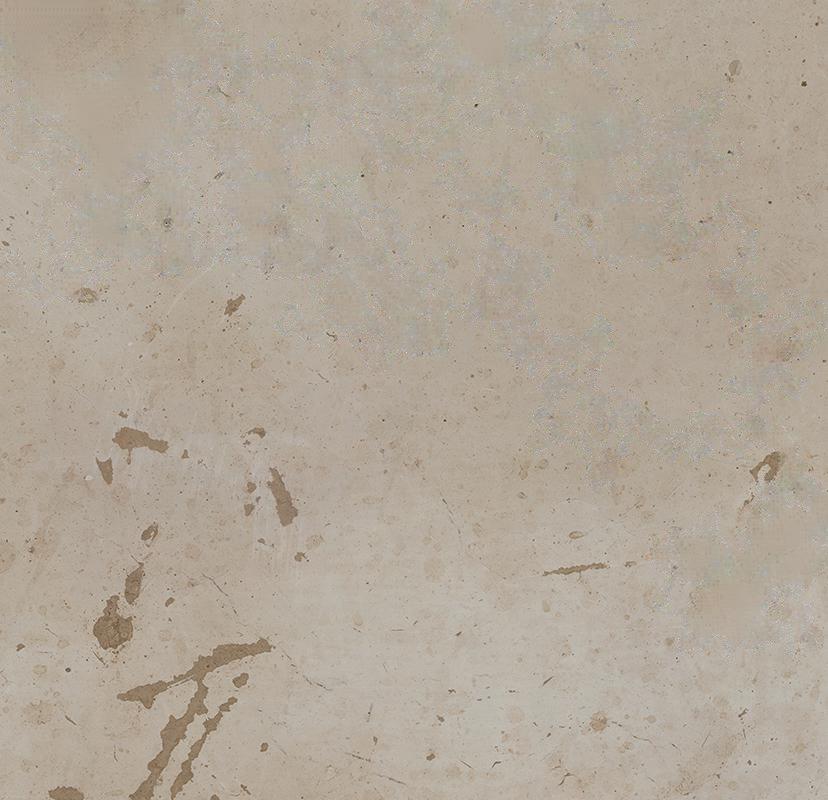

Supplement: Supplementary file 1 [file sensors-21-02091-s001.zip › smartsensors_supplementary_data/nazerietal-model-results/054_masked_1324800_3.jpg]

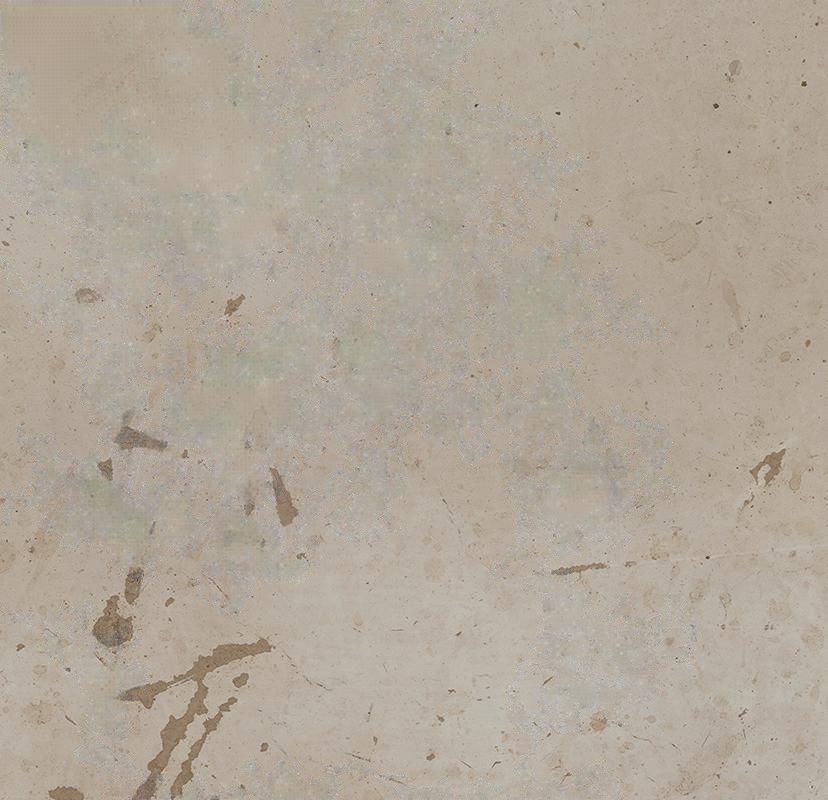

Supplement: Supplementary file 1 [file sensors-21-02091-s001.zip › smartsensors_supplementary_data/nazerietal-model-results/054_masked_2318400_0.jpg]

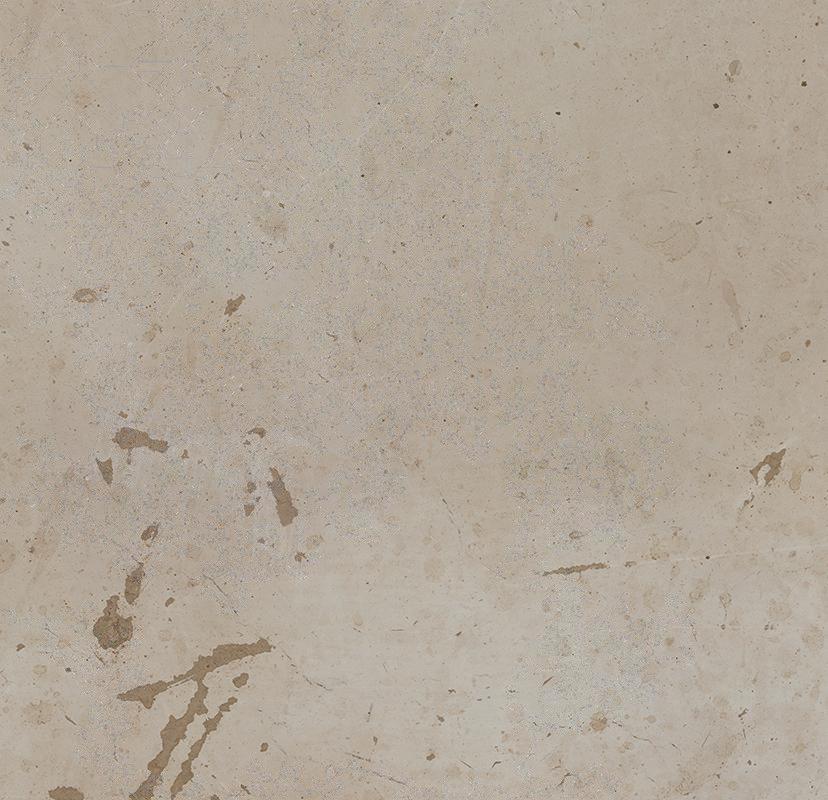

Supplement: Supplementary file 1 [file sensors-21-02091-s001.zip › smartsensors_supplementary_data/nazerietal-model-results/054_masked_2318400_1.jpg]

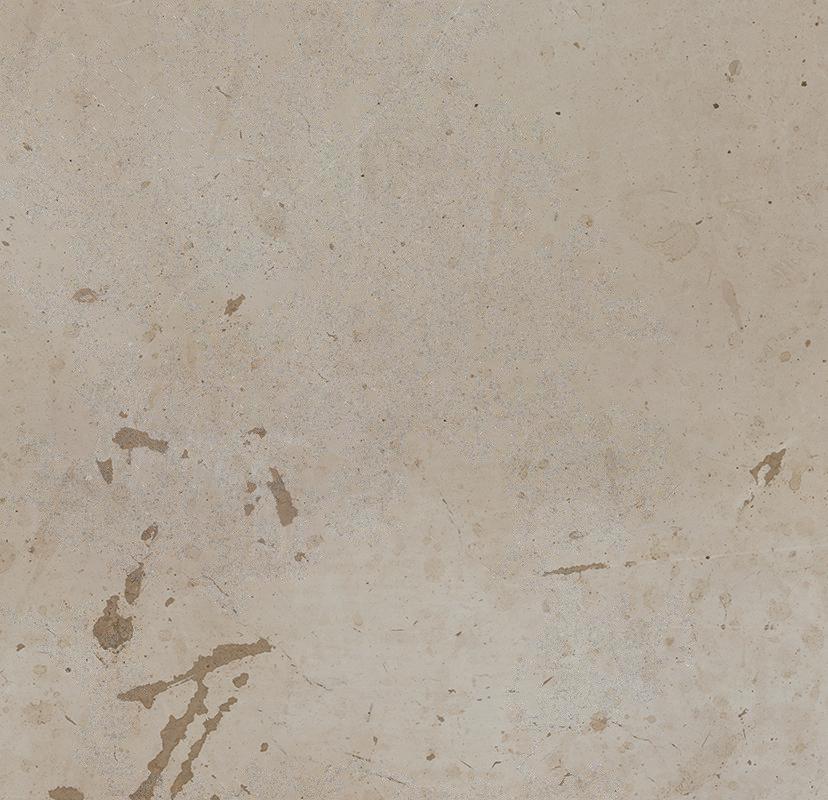

Supplement: Supplementary file 1 [file sensors-21-02091-s001.zip › smartsensors_supplementary_data/nazerietal-model-results/054_masked_2318400_2.jpg]

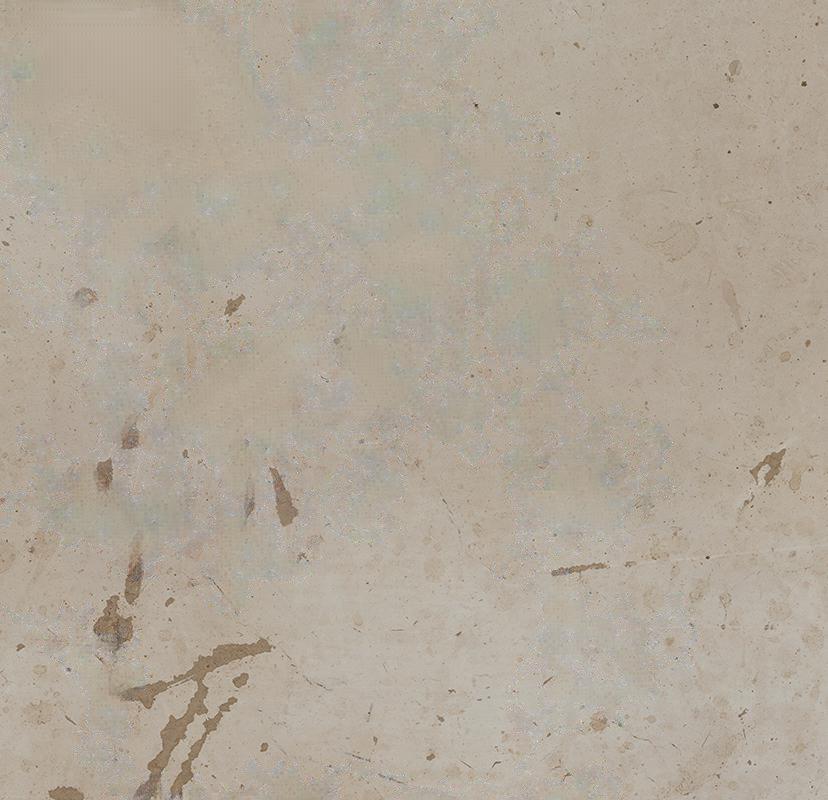

Supplement: Supplementary file 1 [file sensors-21-02091-s001.zip › smartsensors_supplementary_data/nazerietal-model-results/054_masked_2318400_3.jpg]

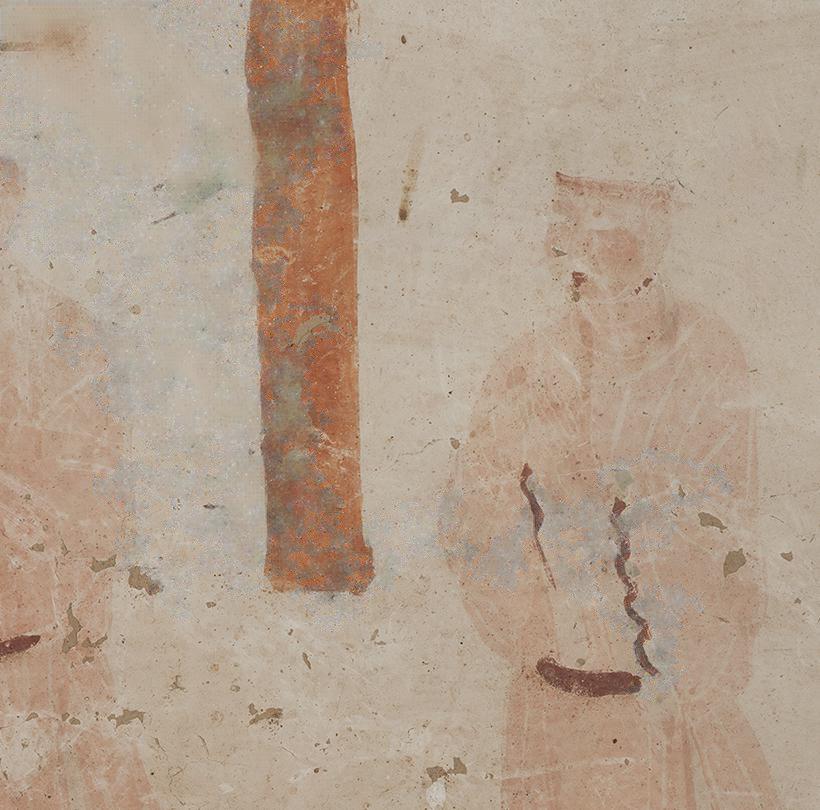

Supplement: Supplementary file 1 [file sensors-21-02091-s001.zip › smartsensors_supplementary_data/nazerietal-model-results/057_masked_1328400_0.jpg]

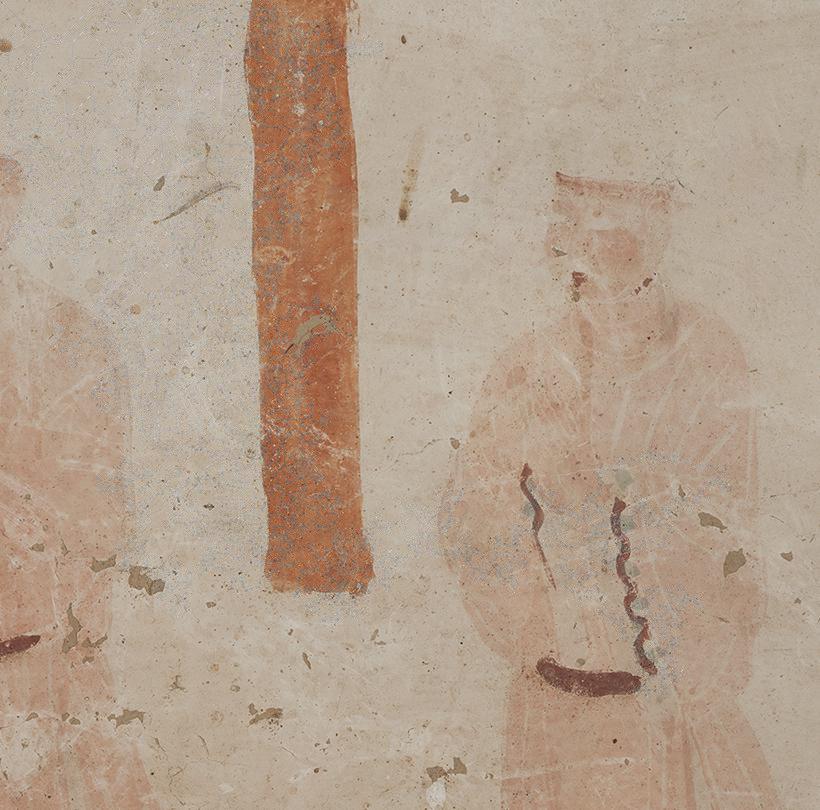

Supplement: Supplementary file 1 [file sensors-21-02091-s001.zip › smartsensors_supplementary_data/nazerietal-model-results/057_masked_1328400_1.jpg]

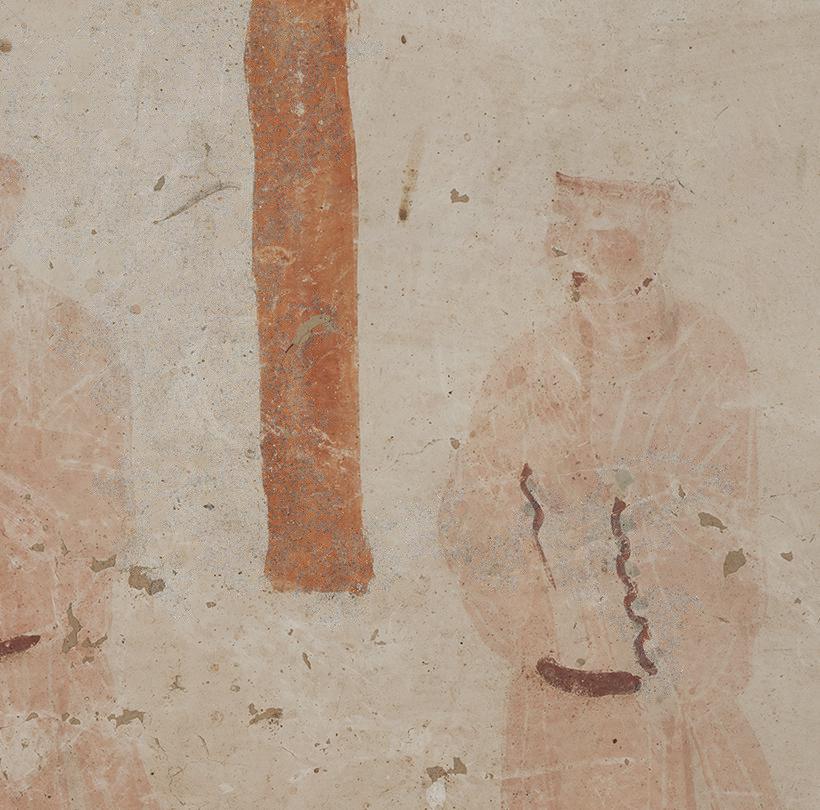

Supplement: Supplementary file 1 [file sensors-21-02091-s001.zip › smartsensors_supplementary_data/nazerietal-model-results/057_masked_1328400_2.jpg]

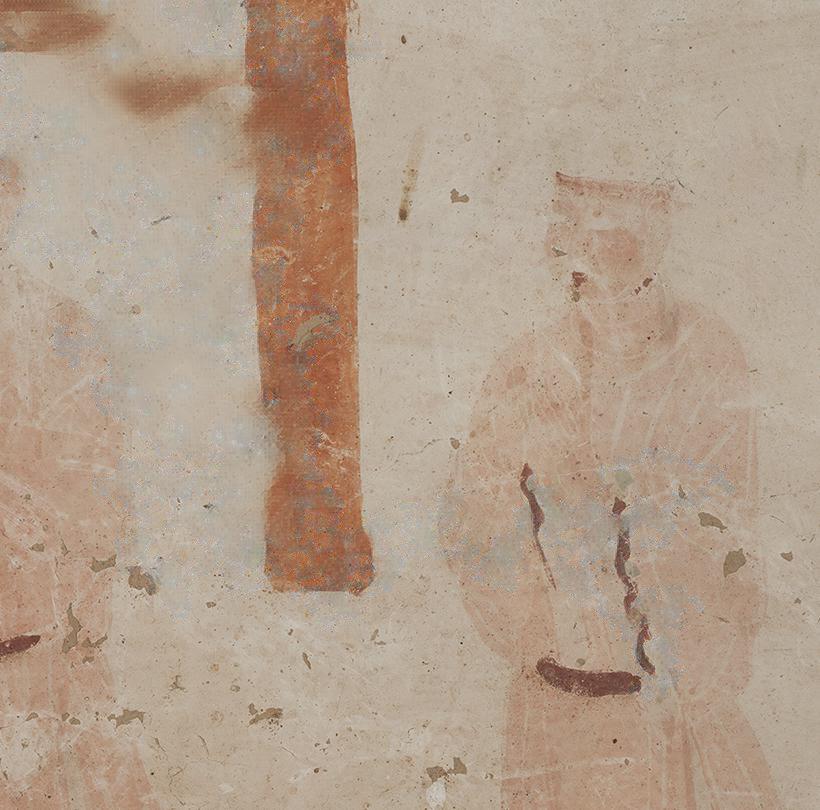

Supplement: Supplementary file 1 [file sensors-21-02091-s001.zip › smartsensors_supplementary_data/nazerietal-model-results/057_masked_1328400_3.jpg]

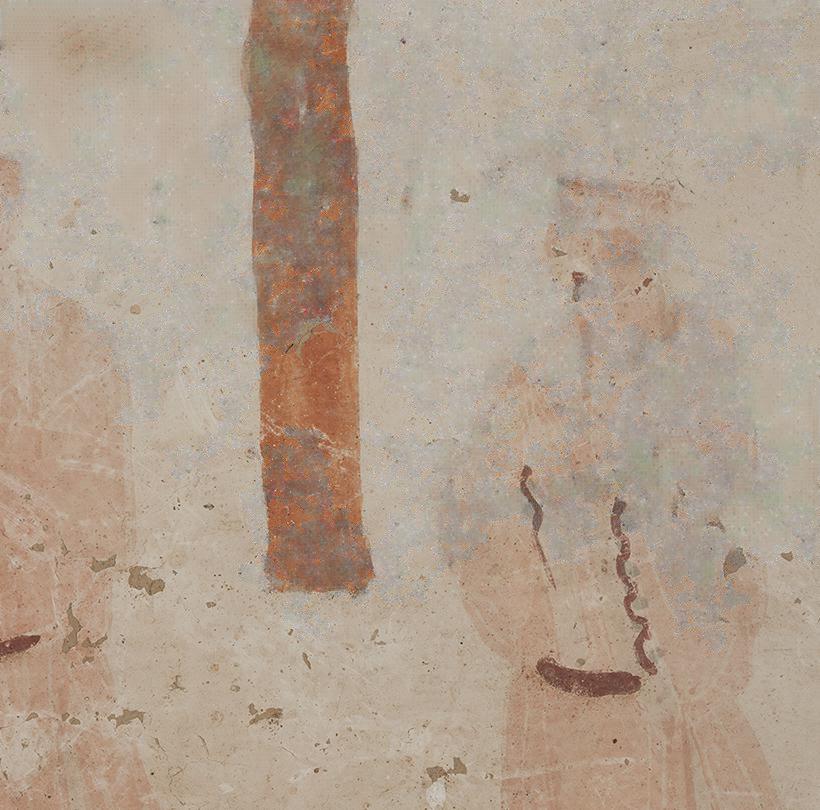

Supplement: Supplementary file 1 [file sensors-21-02091-s001.zip › smartsensors_supplementary_data/nazerietal-model-results/057_masked_2324700_0.jpg]

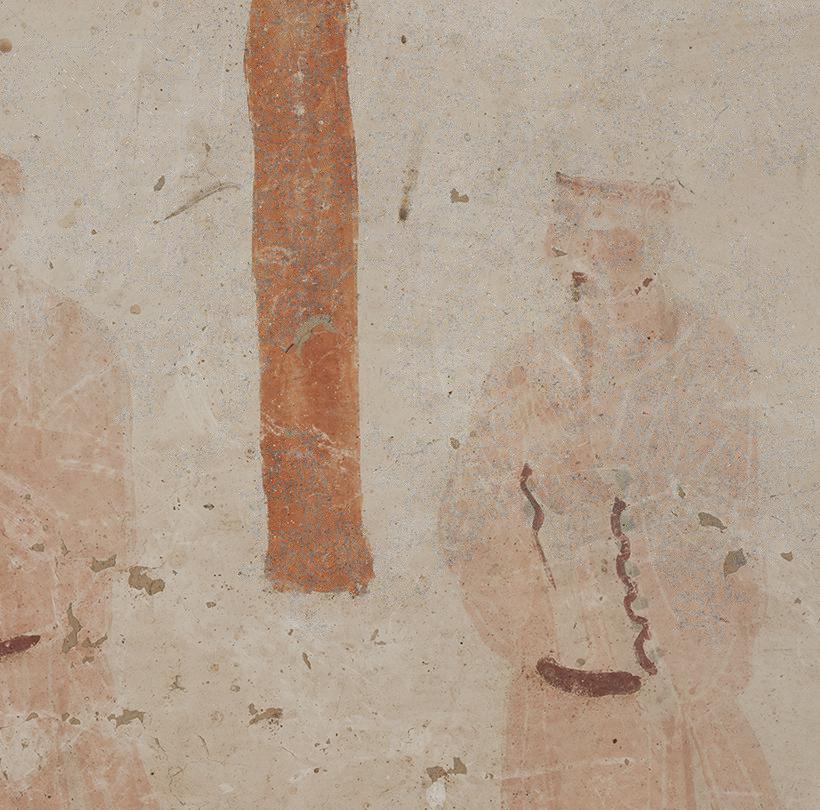

Supplement: Supplementary file 1 [file sensors-21-02091-s001.zip › smartsensors_supplementary_data/nazerietal-model-results/057_masked_2324700_1.jpg]

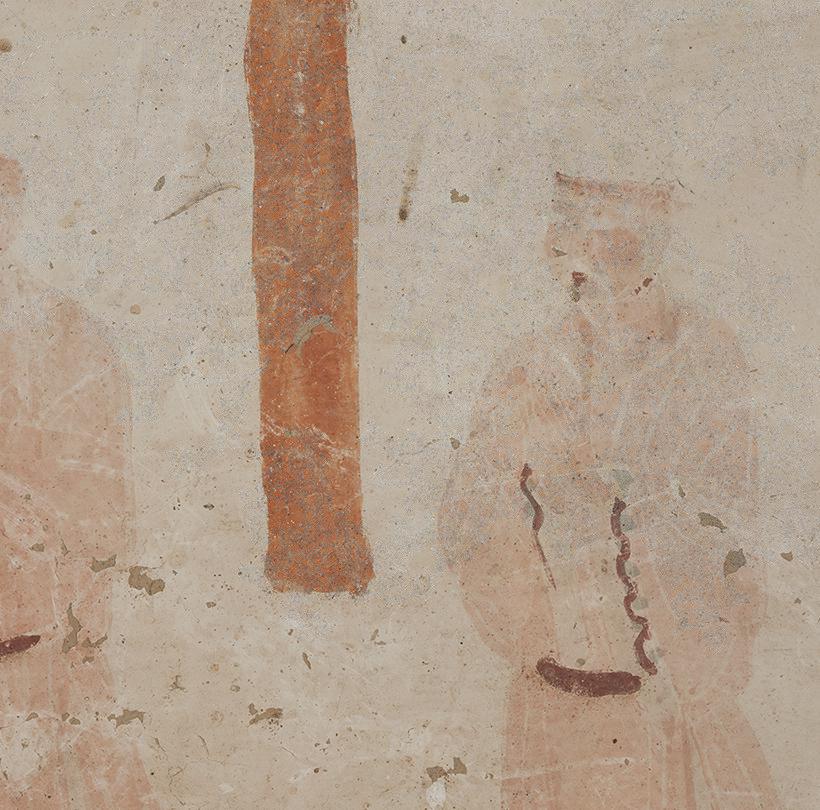

Supplement: Supplementary file 1 [file sensors-21-02091-s001.zip › smartsensors_supplementary_data/nazerietal-model-results/057_masked_2324700_2.jpg]

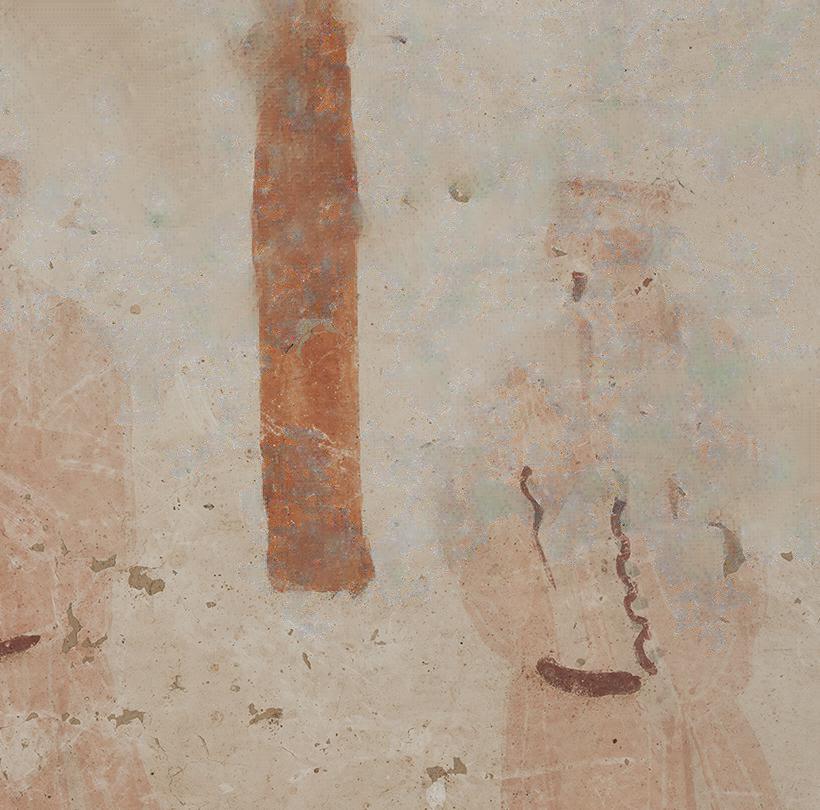

Supplement: Supplementary file 1 [file sensors-21-02091-s001.zip › smartsensors_supplementary_data/nazerietal-model-results/057_masked_2324700_3.jpg]

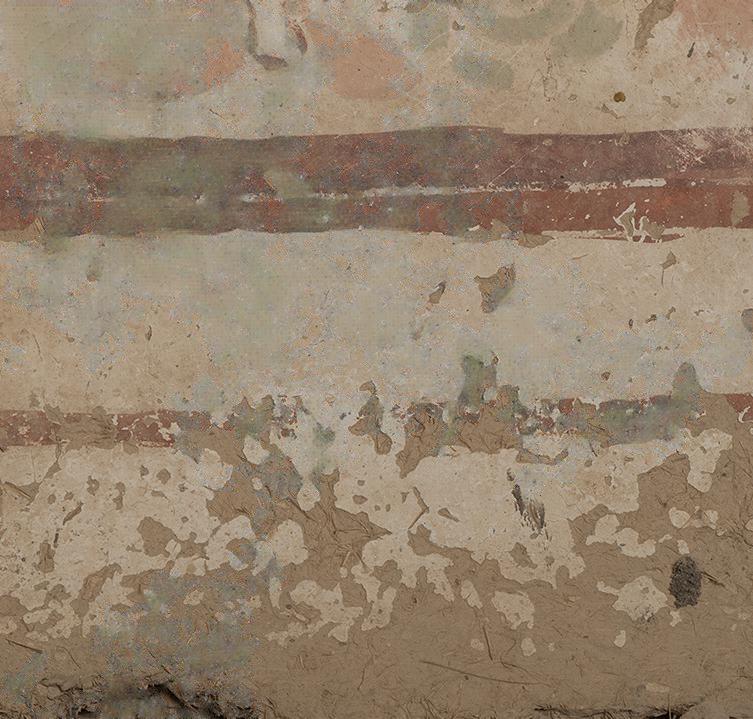

Supplement: Supplementary file 1 [file sensors-21-02091-s001.zip › smartsensors_supplementary_data/nazerietal-model-results/071_masked_1082814_0.jpg]

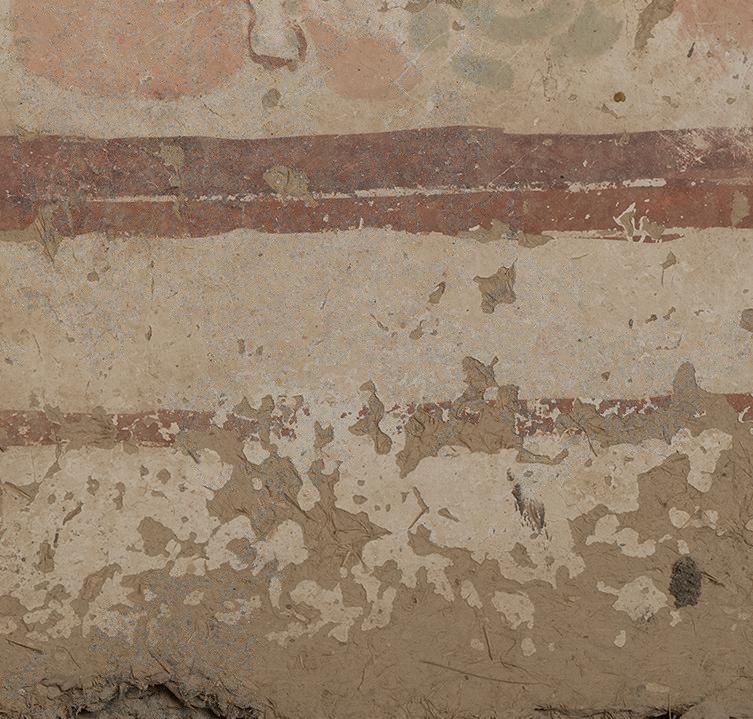

Supplement: Supplementary file 1 [file sensors-21-02091-s001.zip › smartsensors_supplementary_data/nazerietal-model-results/071_masked_1082814_1.jpg]

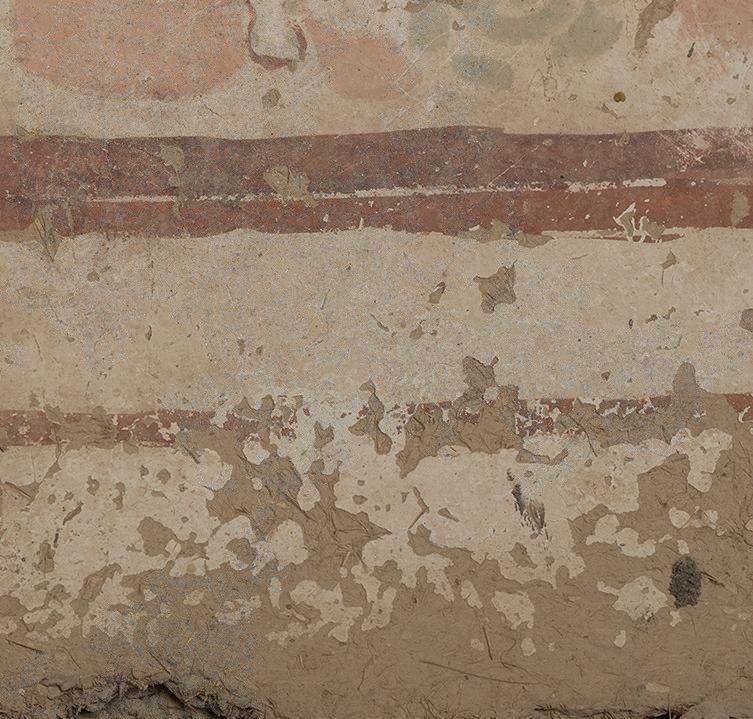

Supplement: Supplementary file 1 [file sensors-21-02091-s001.zip › smartsensors_supplementary_data/nazerietal-model-results/071_masked_1082814_2.jpg]

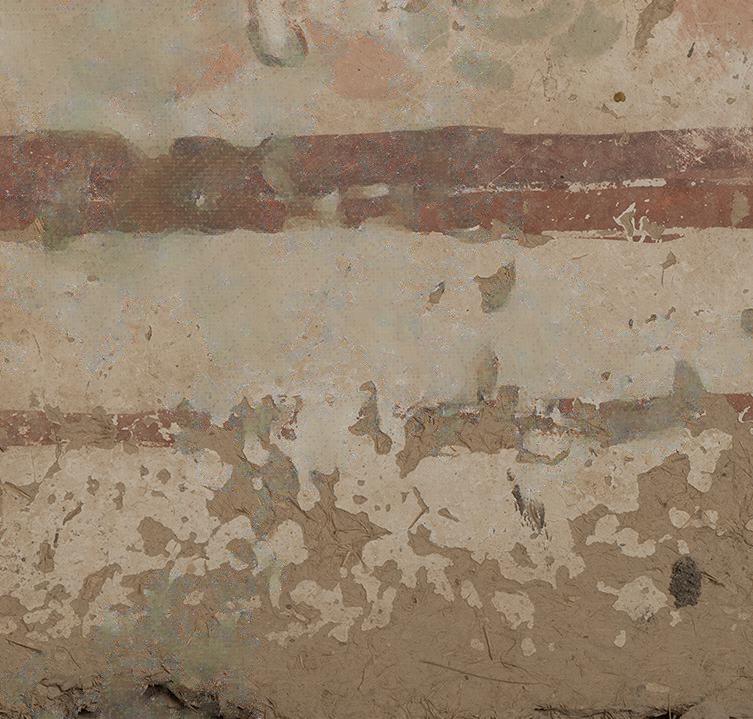

Supplement: Supplementary file 1 [file sensors-21-02091-s001.zip › smartsensors_supplementary_data/nazerietal-model-results/071_masked_1082814_3.jpg]

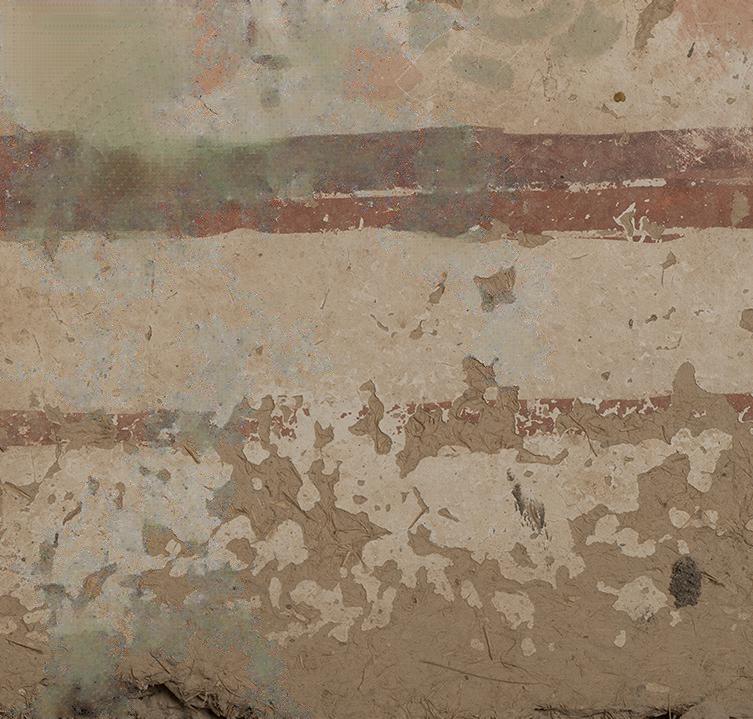

Supplement: Supplementary file 1 [file sensors-21-02091-s001.zip › smartsensors_supplementary_data/nazerietal-model-results/071_masked_1894924_0.jpg]

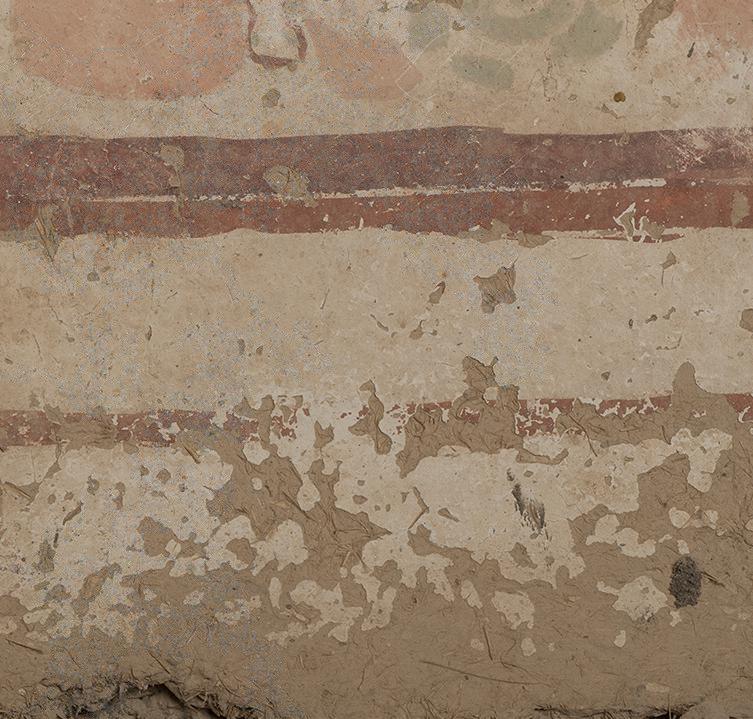

Supplement: Supplementary file 1 [file sensors-21-02091-s001.zip › smartsensors_supplementary_data/nazerietal-model-results/071_masked_1894924_1.jpg]

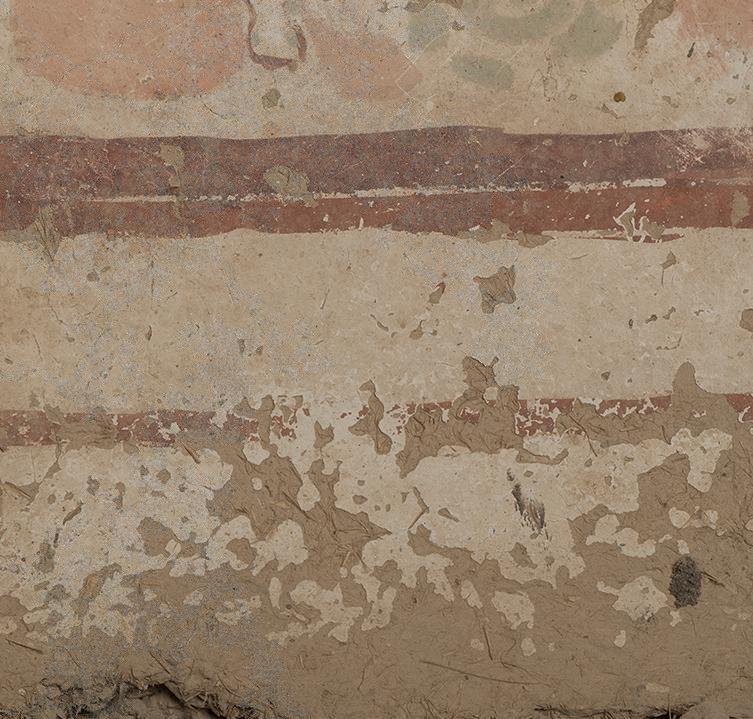

Supplement: Supplementary file 1 [file sensors-21-02091-s001.zip › smartsensors_supplementary_data/nazerietal-model-results/071_masked_1894924_2.jpg]

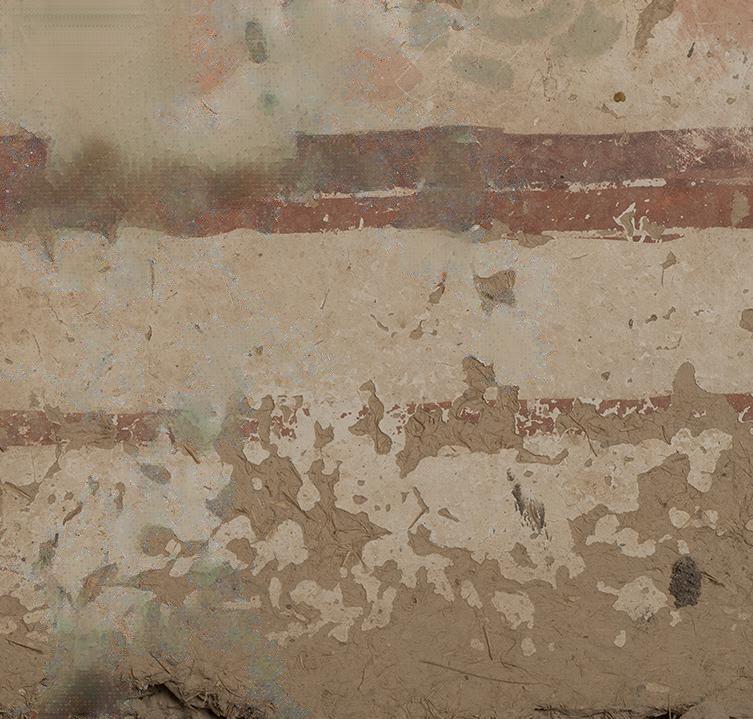

Supplement: Supplementary file 1 [file sensors-21-02091-s001.zip › smartsensors_supplementary_data/nazerietal-model-results/071_masked_1894924_3.jpg]

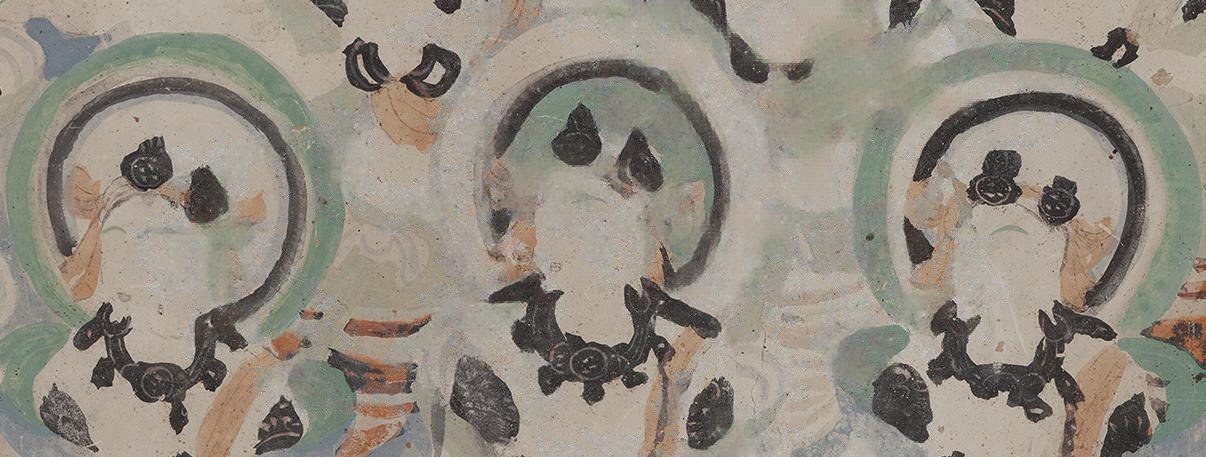

Supplement: Supplementary file 1 [file sensors-21-02091-s001.zip › smartsensors_supplementary_data/nazerietal-model-results/099_masked_1102284_0.jpg]

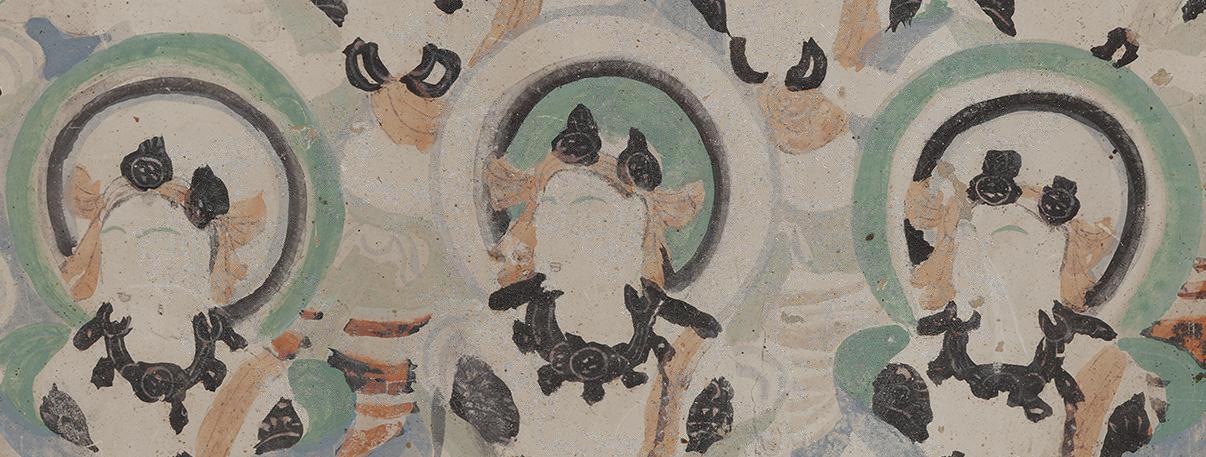

Supplement: Supplementary file 1 [file sensors-21-02091-s001.zip › smartsensors_supplementary_data/nazerietal-model-results/099_masked_1102284_1.jpg]

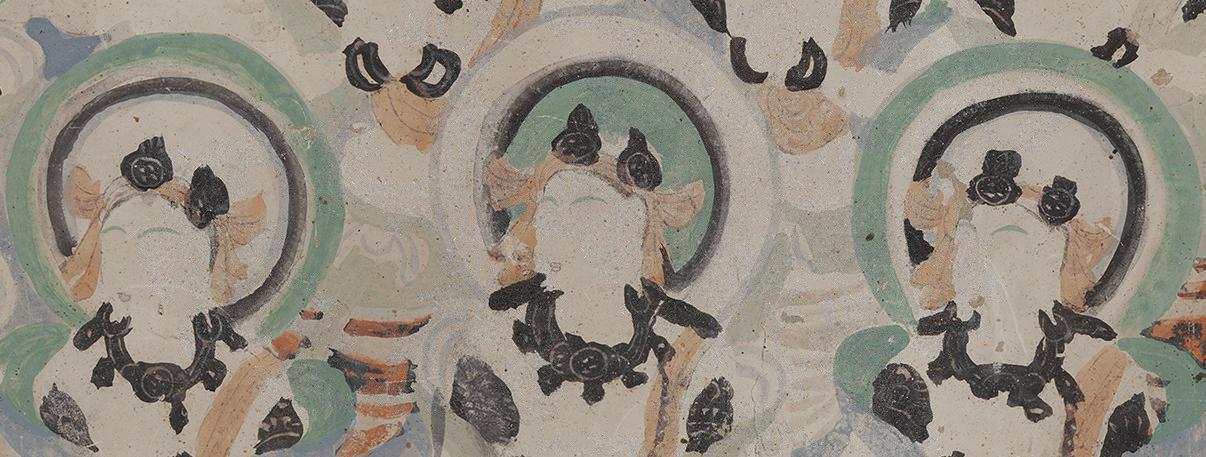

Supplement: Supplementary file 1 [file sensors-21-02091-s001.zip › smartsensors_supplementary_data/nazerietal-model-results/099_masked_1102284_2.jpg]

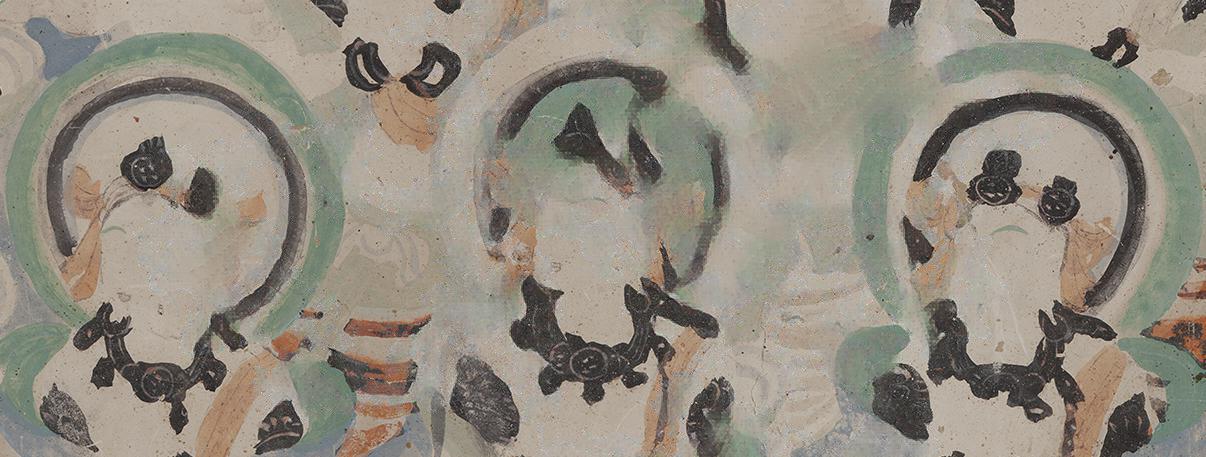

Supplement: Supplementary file 1 [file sensors-21-02091-s001.zip › smartsensors_supplementary_data/nazerietal-model-results/099_masked_1102284_3.jpg]

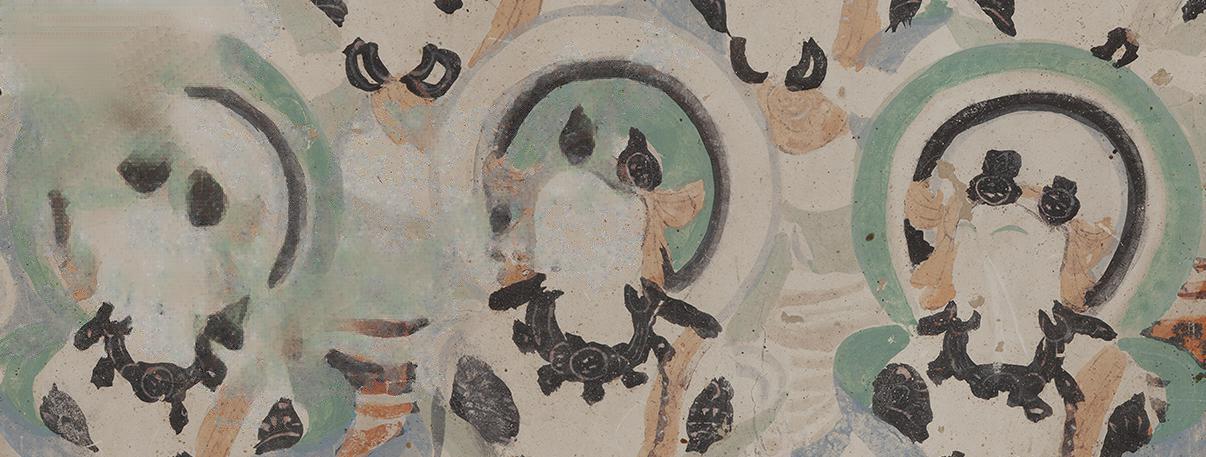

Supplement: Supplementary file 1 [file sensors-21-02091-s001.zip › smartsensors_supplementary_data/nazerietal-model-results/099_masked_1928997_0.jpg]

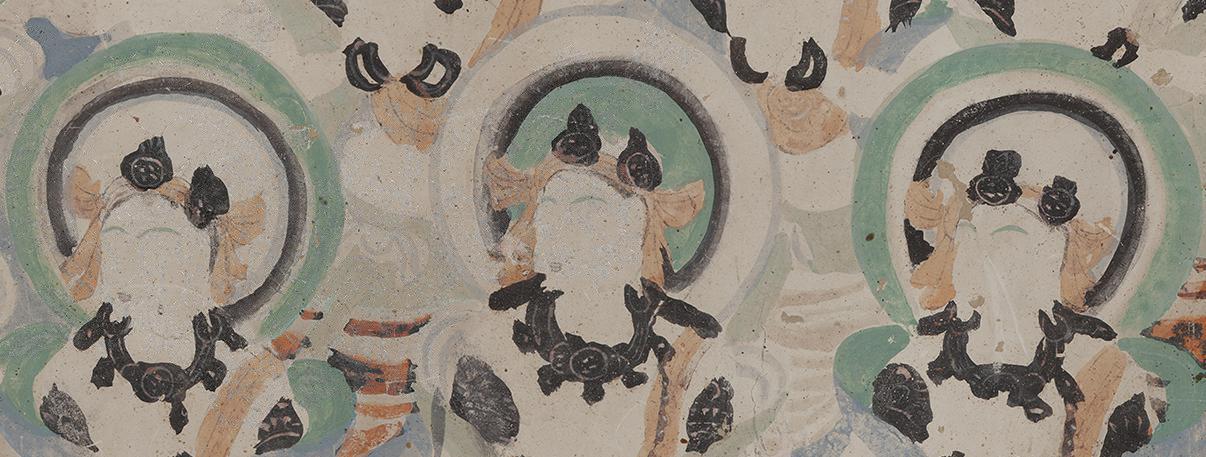

Supplement: Supplementary file 1 [file sensors-21-02091-s001.zip › smartsensors_supplementary_data/nazerietal-model-results/099_masked_1928997_1.jpg]

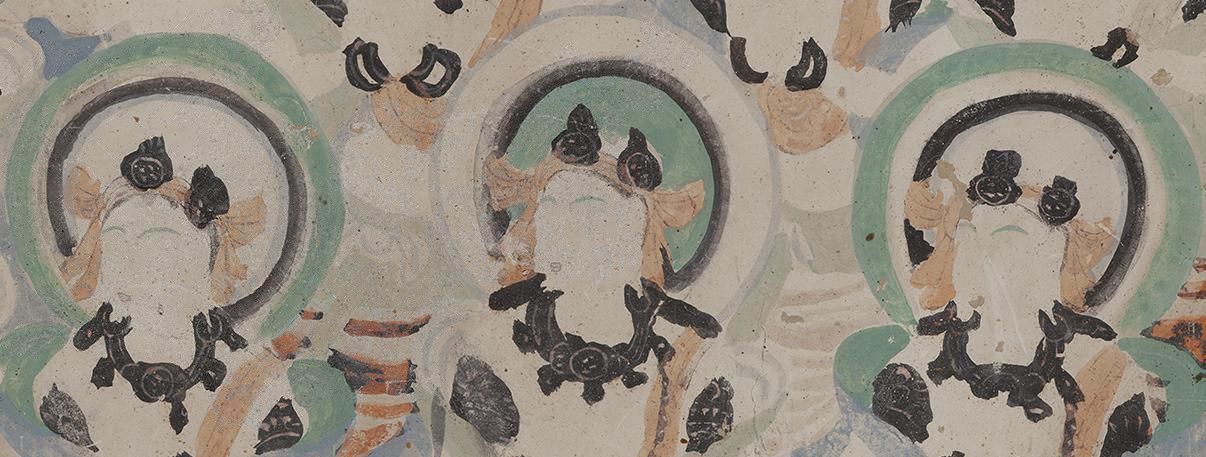

Supplement: Supplementary file 1 [file sensors-21-02091-s001.zip › smartsensors_supplementary_data/nazerietal-model-results/099_masked_1928997_2.jpg]

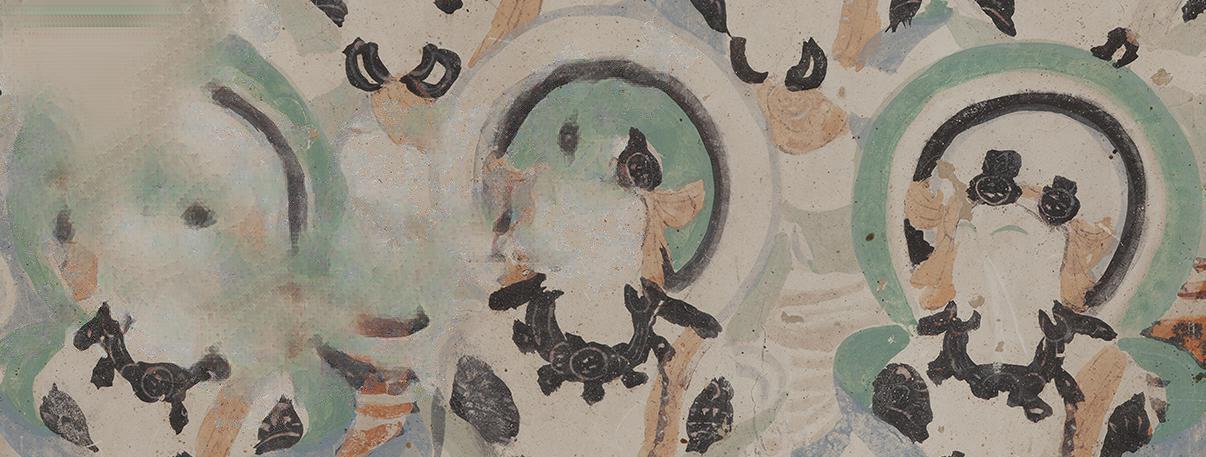

Supplement: Supplementary file 1 [file sensors-21-02091-s001.zip › smartsensors_supplementary_data/nazerietal-model-results/099_masked_1928997_3.jpg]

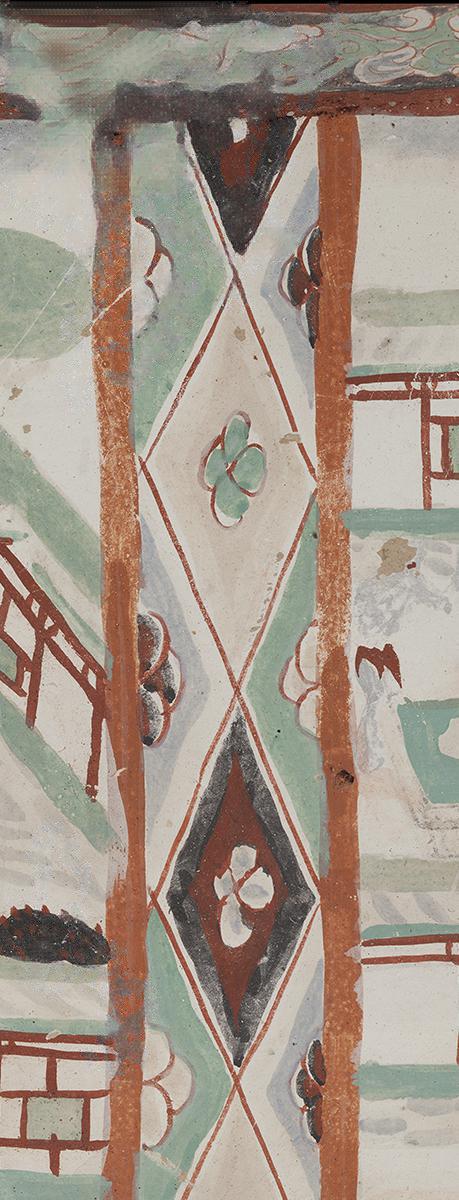

Supplement: Supplementary file 1 [file sensors-21-02091-s001.zip › smartsensors_supplementary_data/nazerietal-model-results/115_masked_1101600_0.jpg]

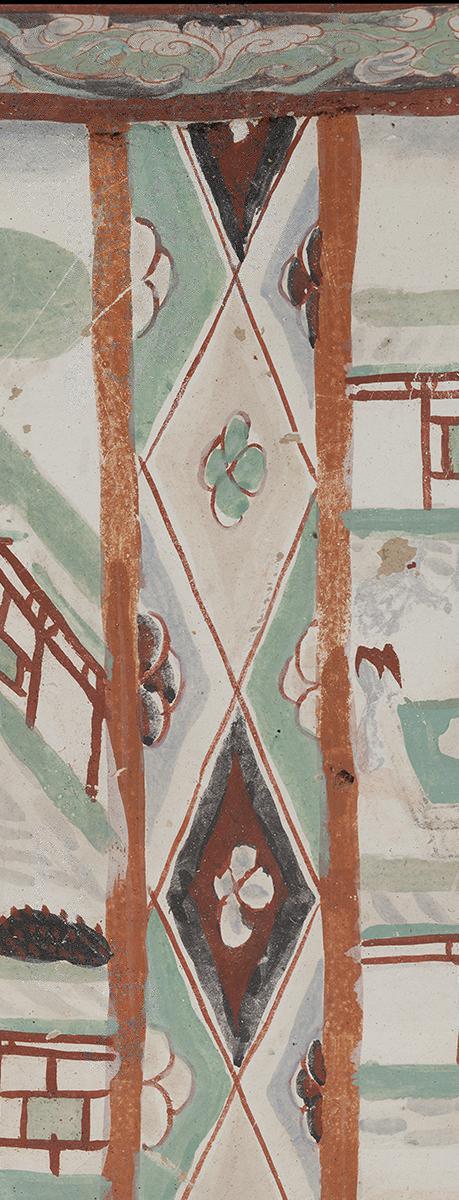

Supplement: Supplementary file 1 [file sensors-21-02091-s001.zip › smartsensors_supplementary_data/nazerietal-model-results/115_masked_1101600_1.jpg]

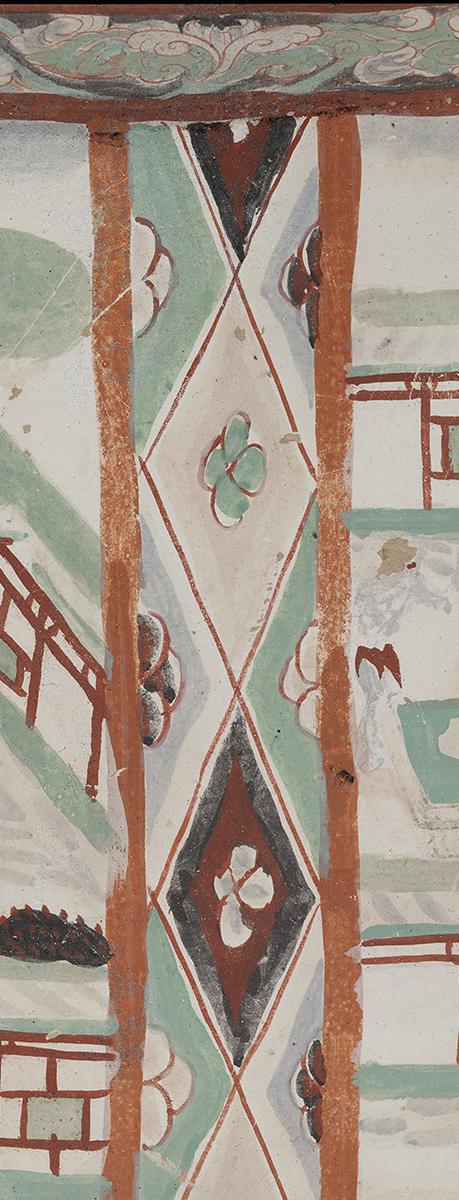

Supplement: Supplementary file 1 [file sensors-21-02091-s001.zip › smartsensors_supplementary_data/nazerietal-model-results/115_masked_1101600_2.jpg]

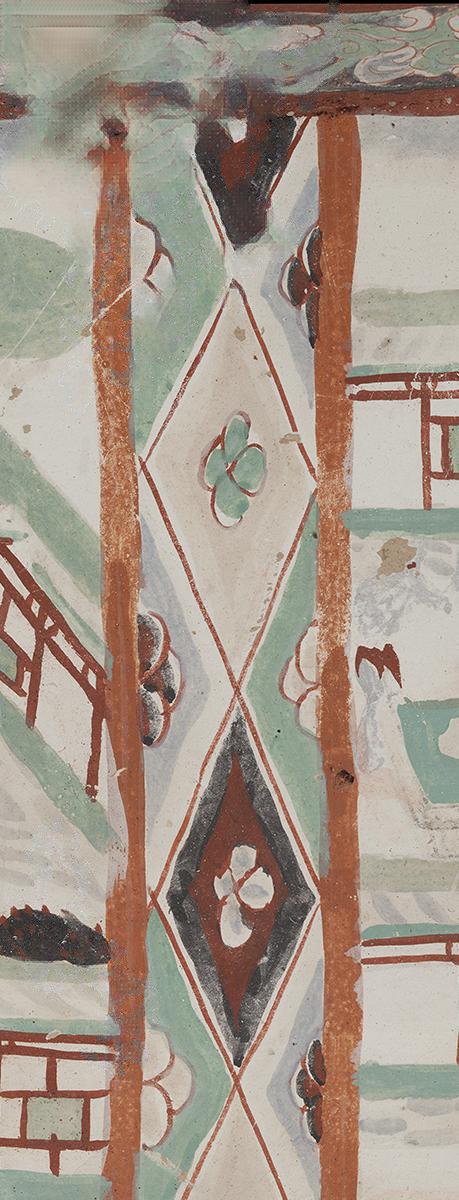

Supplement: Supplementary file 1 [file sensors-21-02091-s001.zip › smartsensors_supplementary_data/nazerietal-model-results/115_masked_1101600_3.jpg]

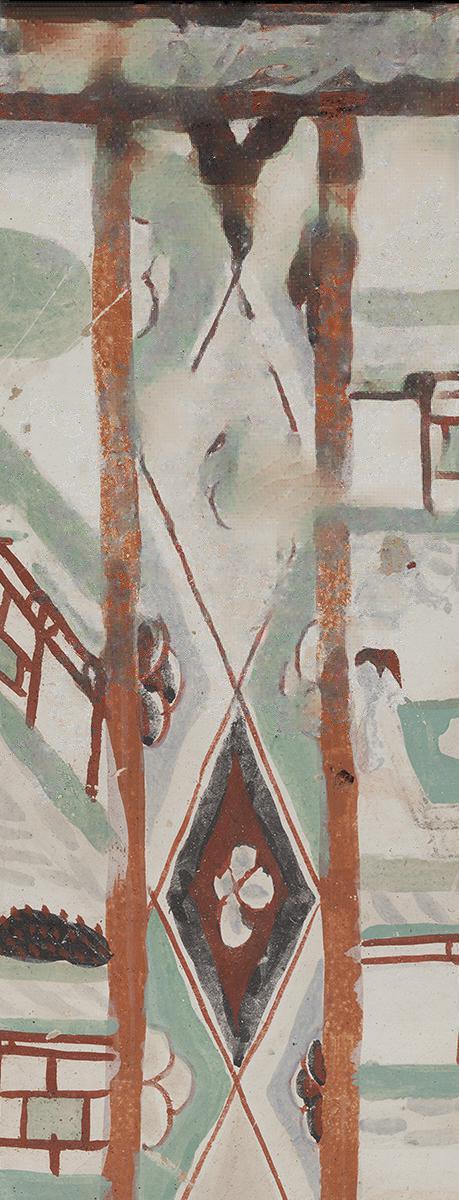

Supplement: Supplementary file 1 [file sensors-21-02091-s001.zip › smartsensors_supplementary_data/nazerietal-model-results/115_masked_1927800_0.jpg]

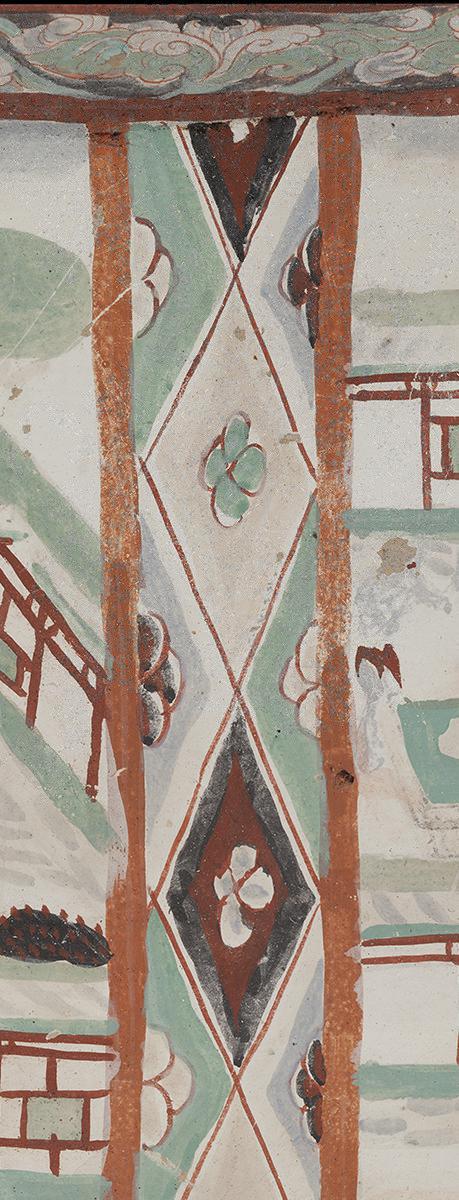

Supplement: Supplementary file 1 [file sensors-21-02091-s001.zip › smartsensors_supplementary_data/nazerietal-model-results/115_masked_1927800_1.jpg]

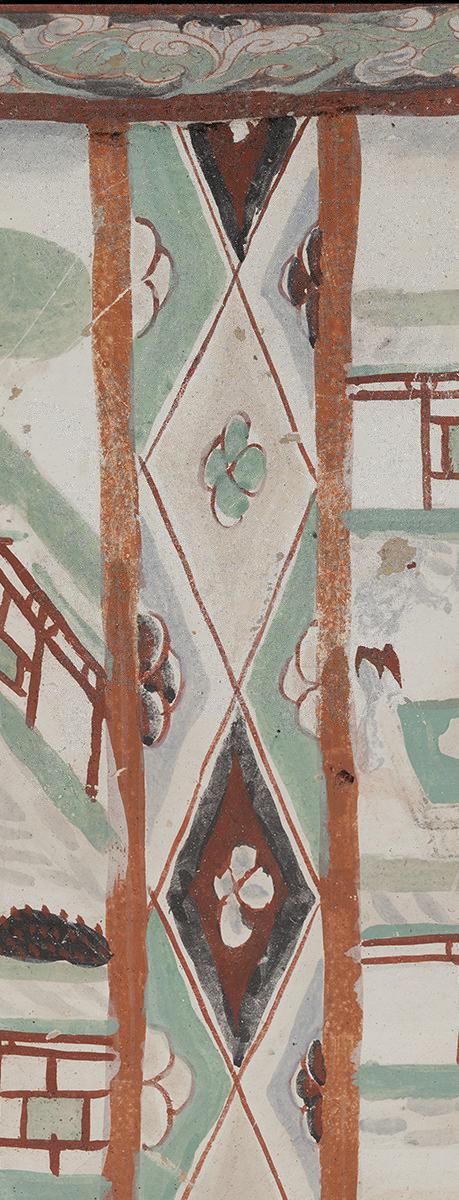

Supplement: Supplementary file 1 [file sensors-21-02091-s001.zip › smartsensors_supplementary_data/nazerietal-model-results/115_masked_1927800_2.jpg]

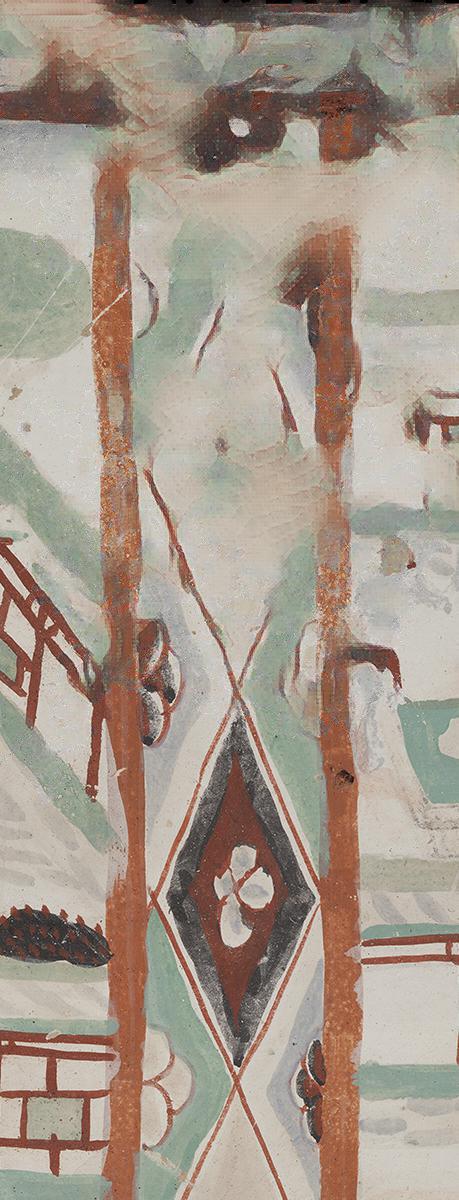

Supplement: Supplementary file 1 [file sensors-21-02091-s001.zip › smartsensors_supplementary_data/nazerietal-model-results/115_masked_1927800_3.jpg]

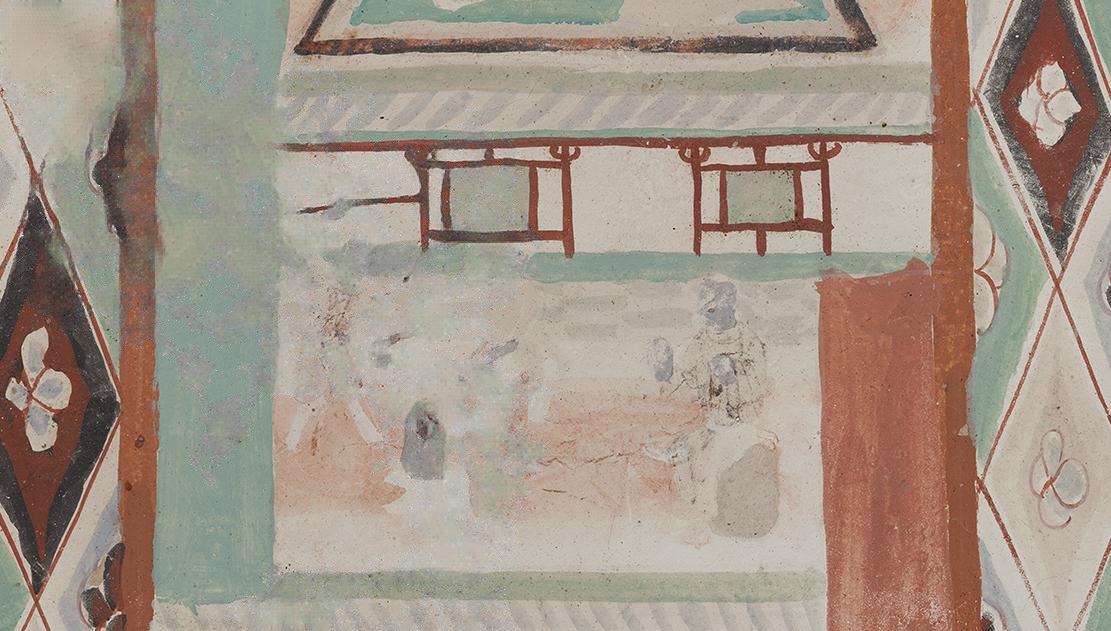

Supplement: Supplementary file 1 [file sensors-21-02091-s001.zip › smartsensors_supplementary_data/nazerietal-model-results/119_masked_1402082_0.jpg]

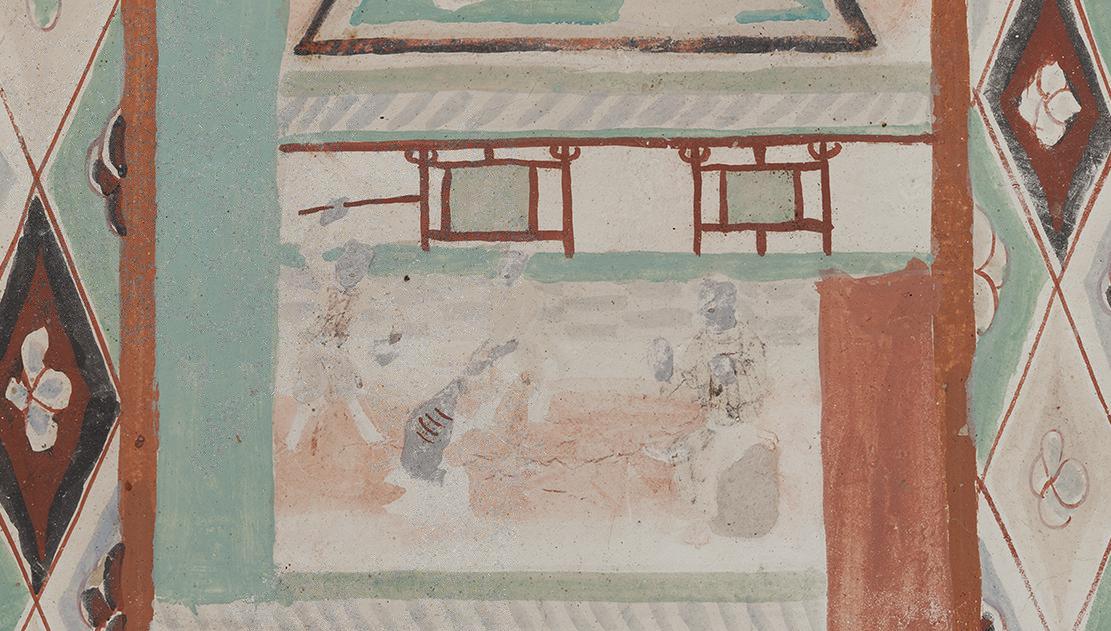

Supplement: Supplementary file 1 [file sensors-21-02091-s001.zip › smartsensors_supplementary_data/nazerietal-model-results/119_masked_1402082_1.jpg]

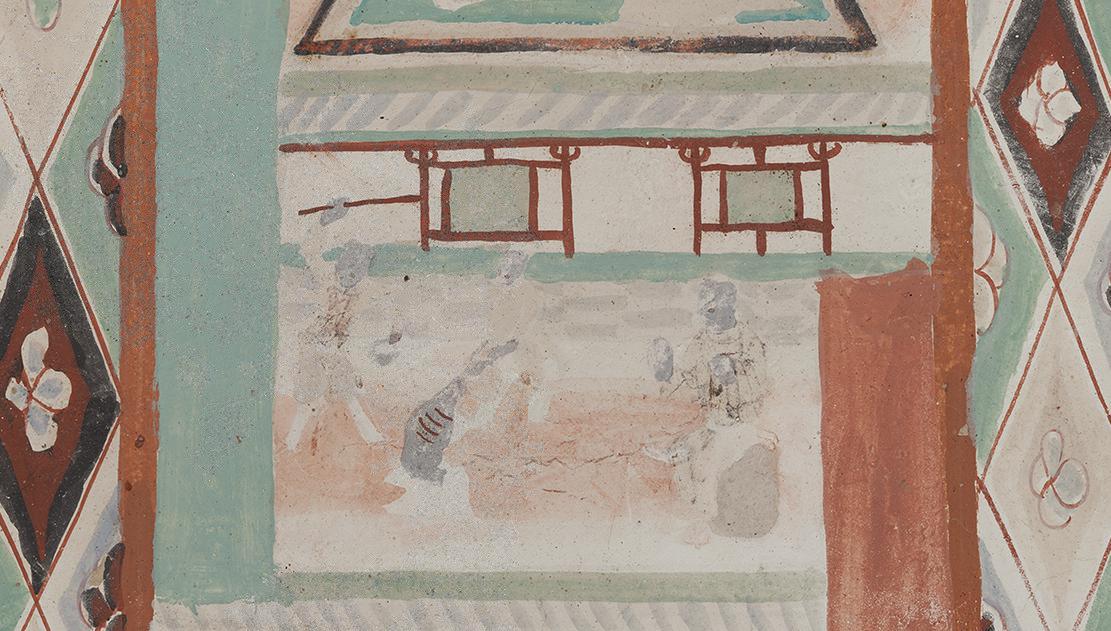

Supplement: Supplementary file 1 [file sensors-21-02091-s001.zip › smartsensors_supplementary_data/nazerietal-model-results/119_masked_1402082_2.jpg]

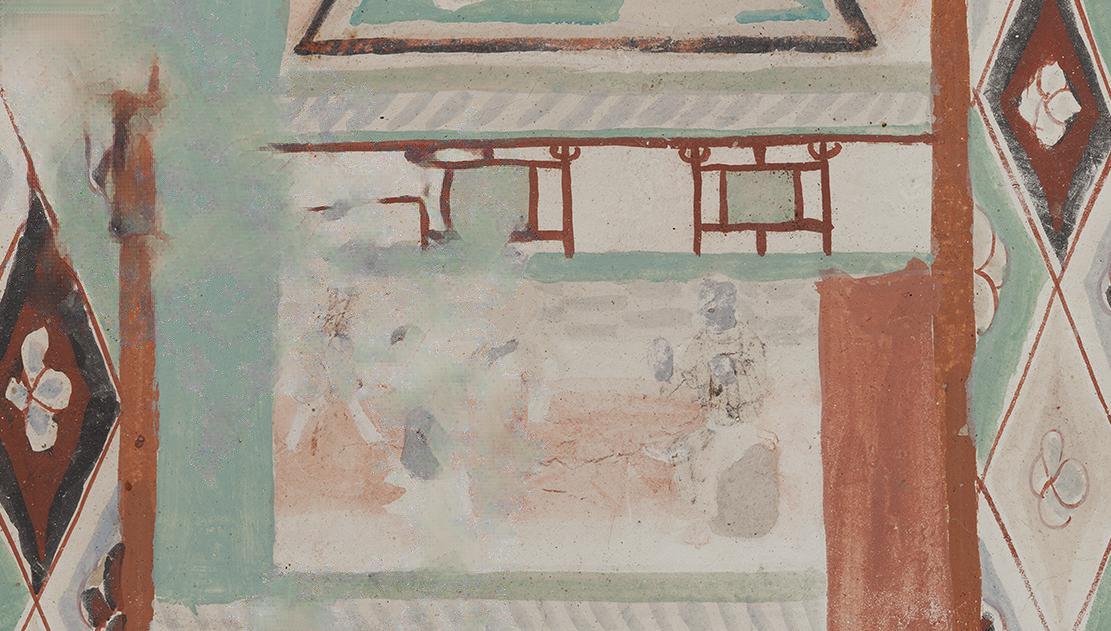

Supplement: Supplementary file 1 [file sensors-21-02091-s001.zip › smartsensors_supplementary_data/nazerietal-model-results/119_masked_1402082_3.jpg]

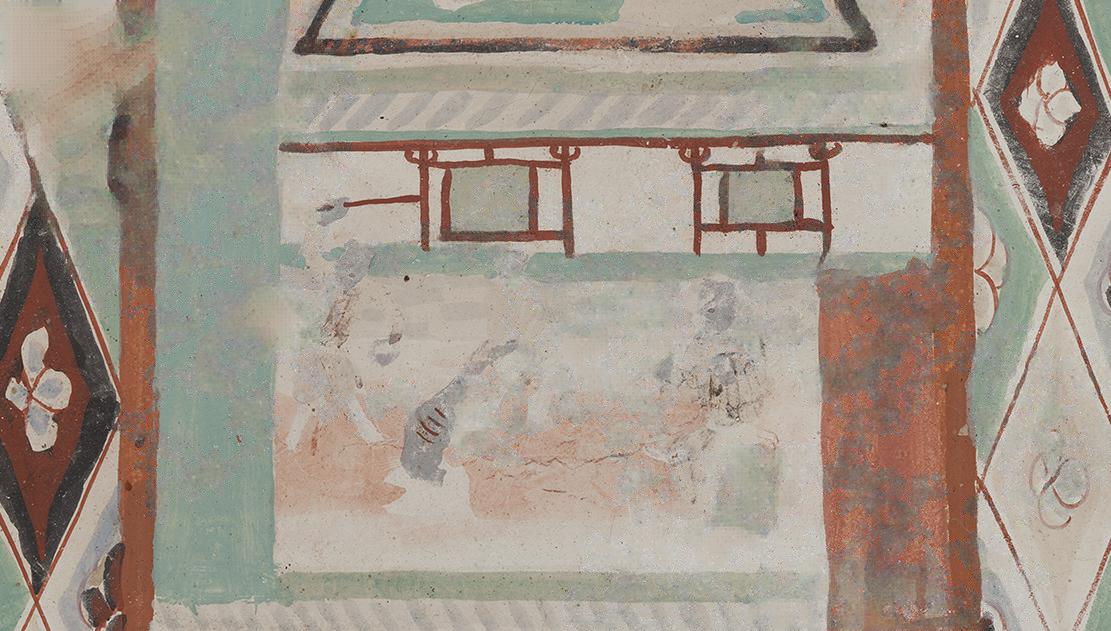

Supplement: Supplementary file 1 [file sensors-21-02091-s001.zip › smartsensors_supplementary_data/nazerietal-model-results/119_masked_2453643_0.jpg]

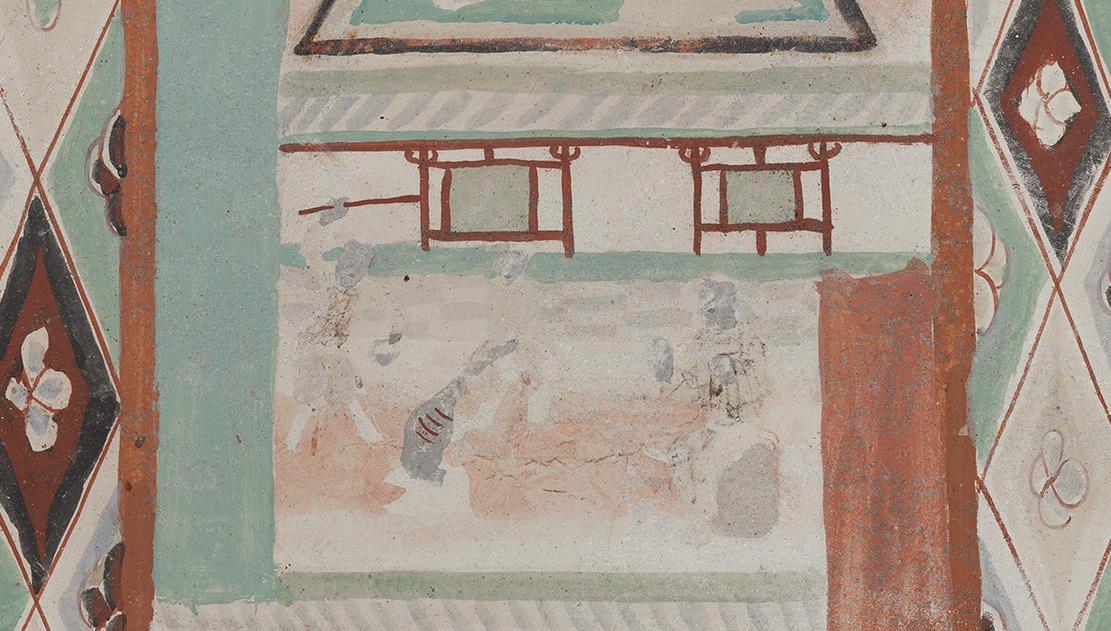

Supplement: Supplementary file 1 [file sensors-21-02091-s001.zip › smartsensors_supplementary_data/nazerietal-model-results/119_masked_2453643_1.jpg]

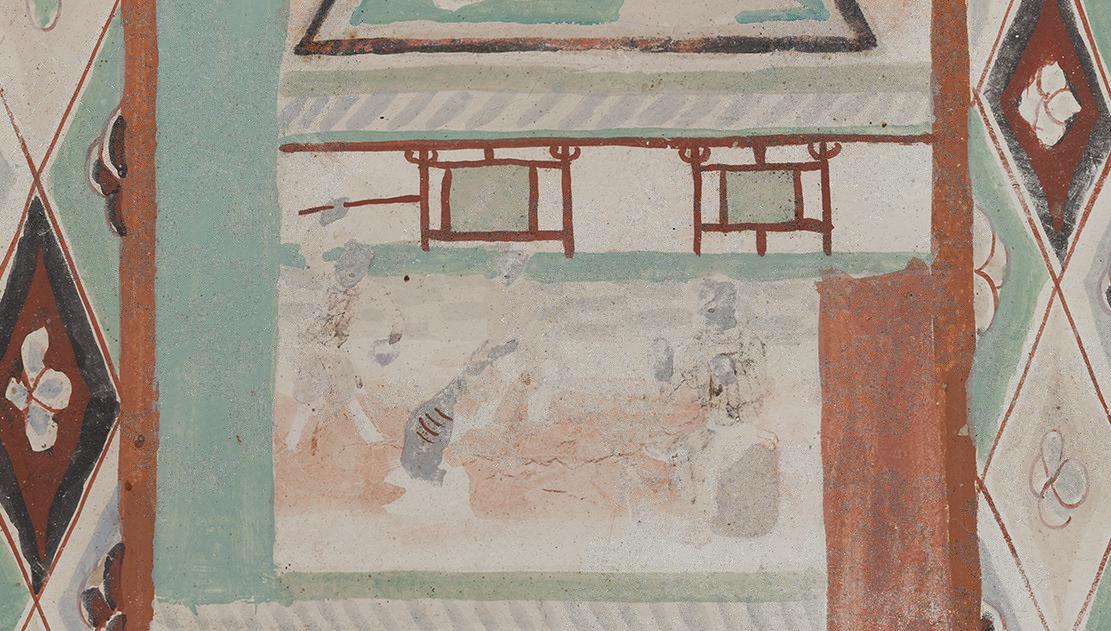

Supplement: Supplementary file 1 [file sensors-21-02091-s001.zip › smartsensors_supplementary_data/nazerietal-model-results/119_masked_2453643_2.jpg]

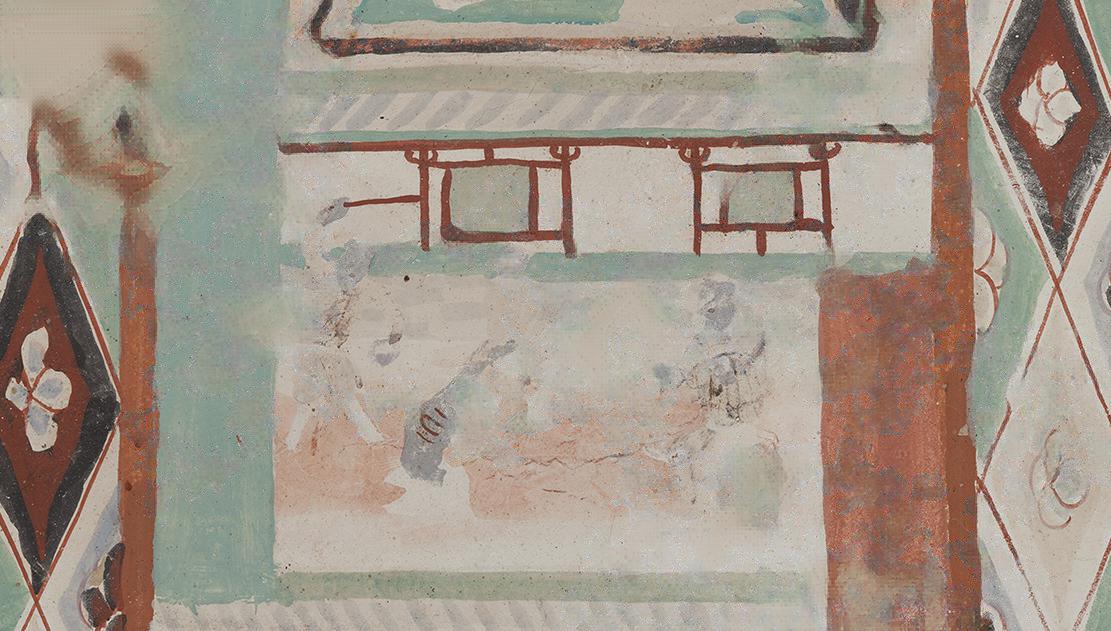

Supplement: Supplementary file 1 [file sensors-21-02091-s001.zip › smartsensors_supplementary_data/nazerietal-model-results/119_masked_2453643_3.jpg]

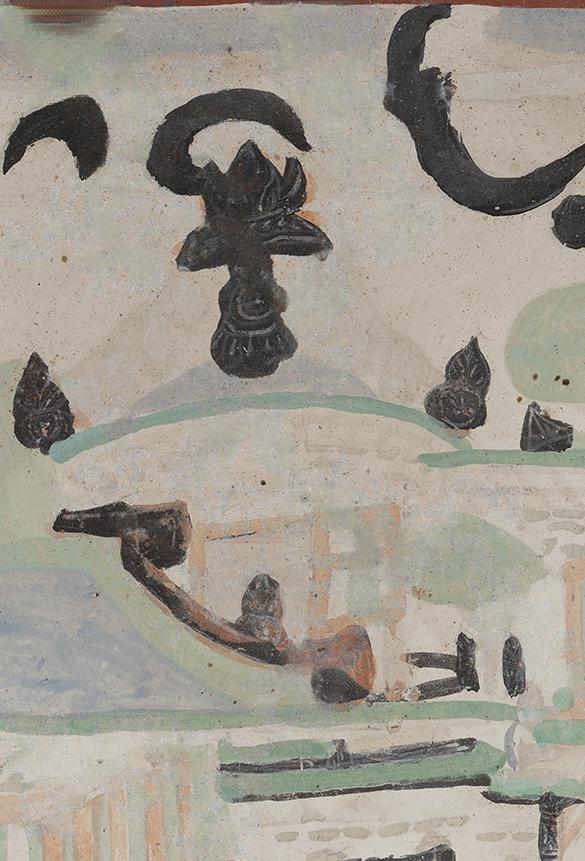

Supplement: Supplementary file 1 [file sensors-21-02091-s001.zip › smartsensors_supplementary_data/nazerietal-model-results/131_masked_1007370_0.jpg]

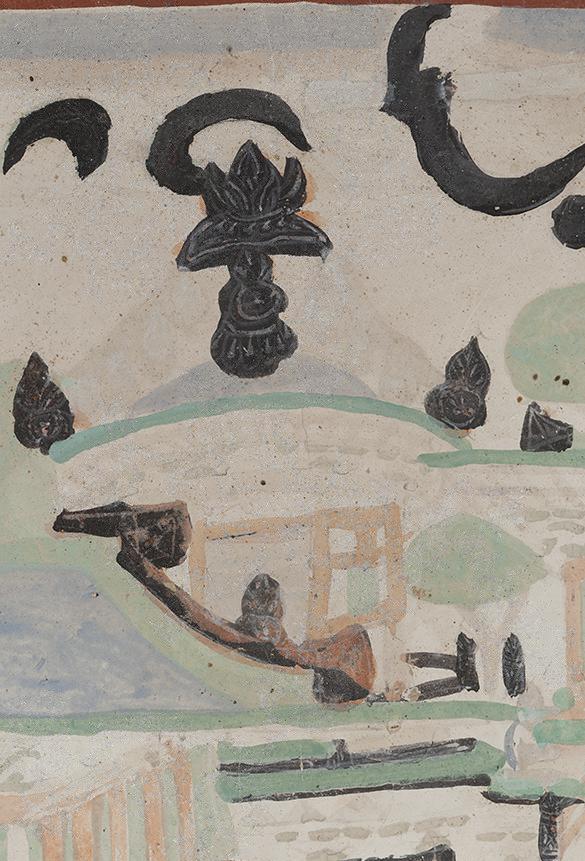

Supplement: Supplementary file 1 [file sensors-21-02091-s001.zip › smartsensors_supplementary_data/nazerietal-model-results/131_masked_1007370_1.jpg]

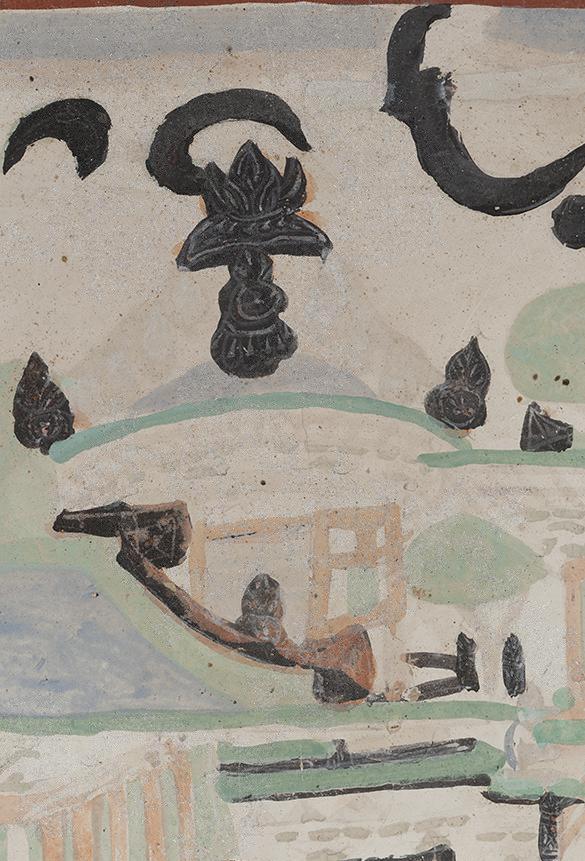

Supplement: Supplementary file 1 [file sensors-21-02091-s001.zip › smartsensors_supplementary_data/nazerietal-model-results/131_masked_1007370_2.jpg]

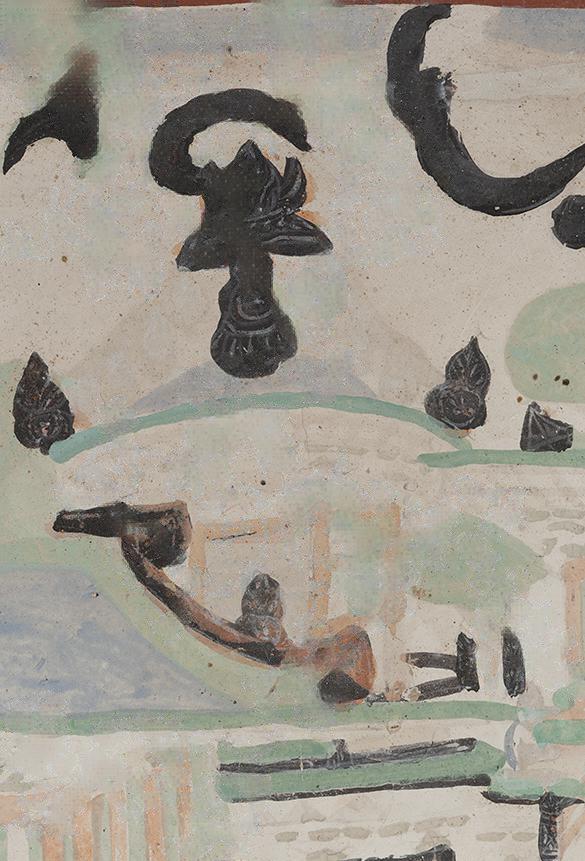

Supplement: Supplementary file 1 [file sensors-21-02091-s001.zip › smartsensors_supplementary_data/nazerietal-model-results/131_masked_1007370_3.jpg]

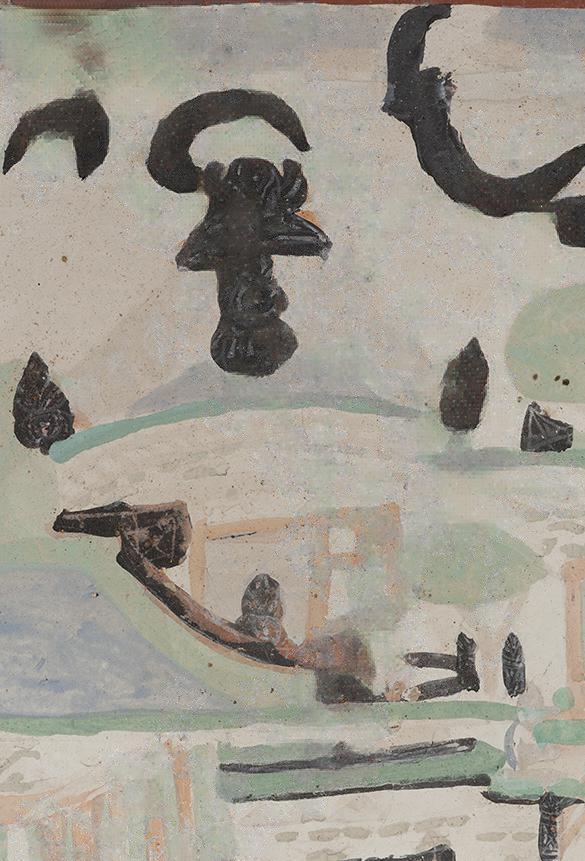

Supplement: Supplementary file 1 [file sensors-21-02091-s001.zip › smartsensors_supplementary_data/nazerietal-model-results/131_masked_1762897_0.jpg]

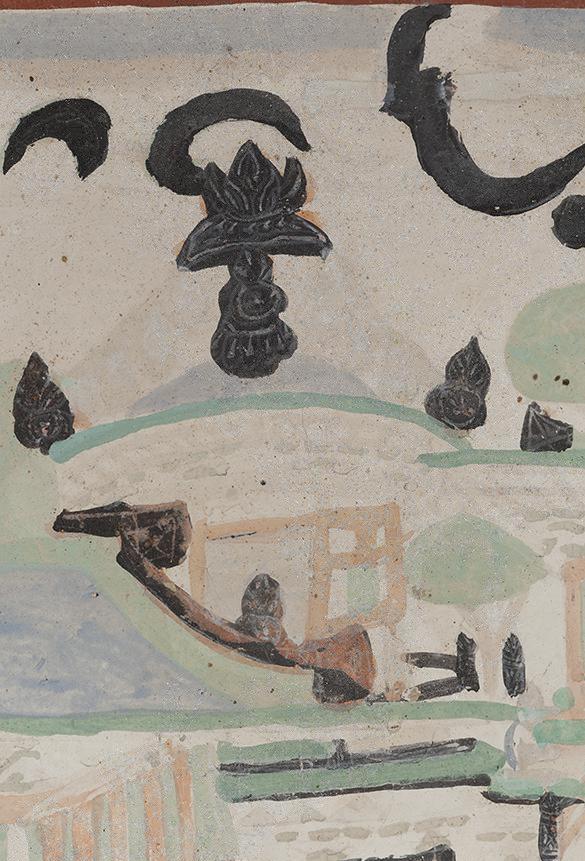

Supplement: Supplementary file 1 [file sensors-21-02091-s001.zip › smartsensors_supplementary_data/nazerietal-model-results/131_masked_1762897_1.jpg]

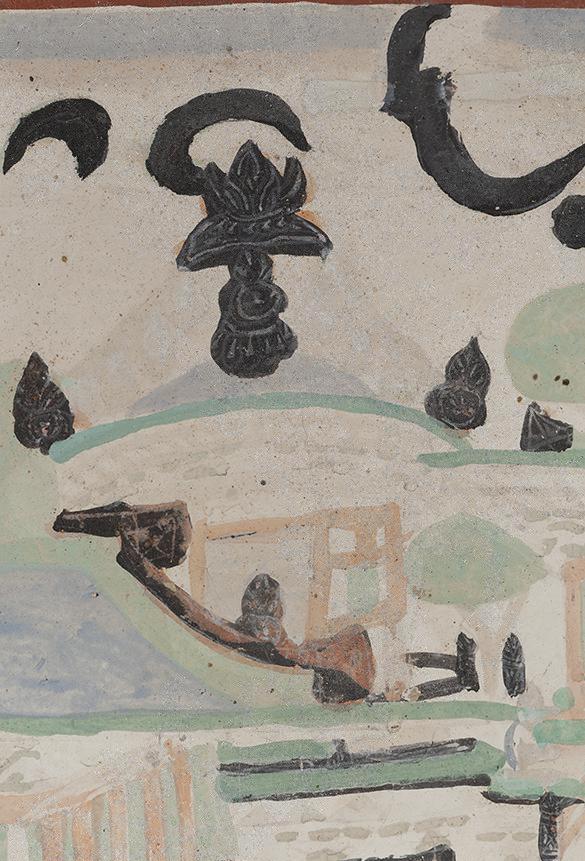

Supplement: Supplementary file 1 [file sensors-21-02091-s001.zip › smartsensors_supplementary_data/nazerietal-model-results/131_masked_1762897_2.jpg]

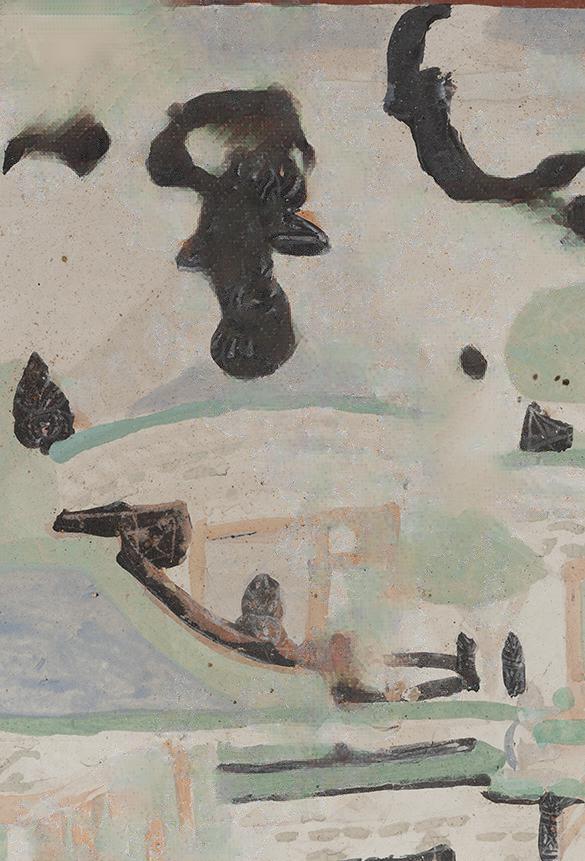

Supplement: Supplementary file 1 [file sensors-21-02091-s001.zip › smartsensors_supplementary_data/nazerietal-model-results/131_masked_1762897_3.jpg]

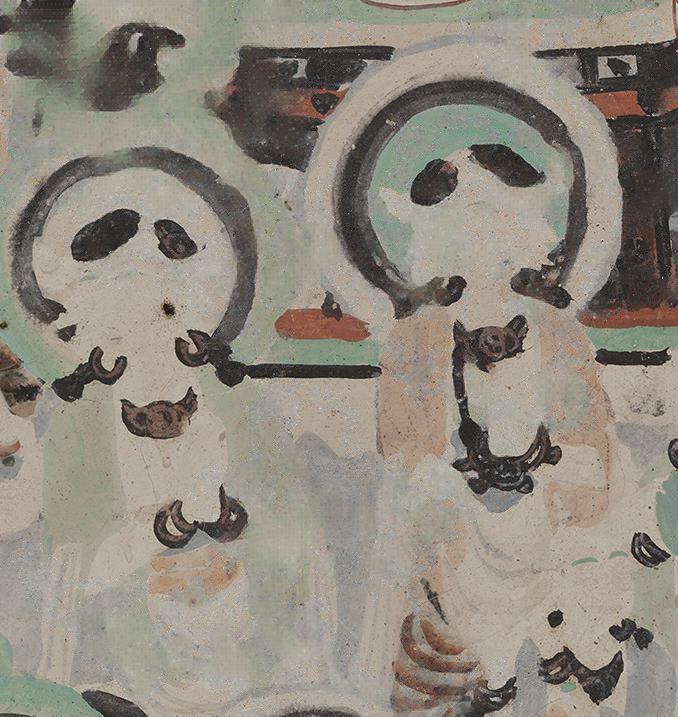

Supplement: Supplementary file 1 [file sensors-21-02091-s001.zip › smartsensors_supplementary_data/nazerietal-model-results/146_masked_1701441_0.jpg]

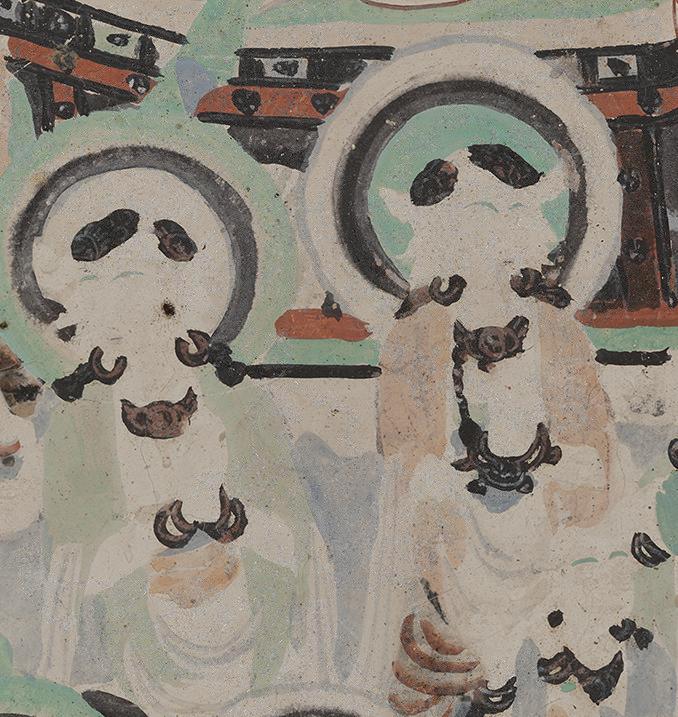

Supplement: Supplementary file 1 [file sensors-21-02091-s001.zip › smartsensors_supplementary_data/nazerietal-model-results/146_masked_1701441_1.jpg]

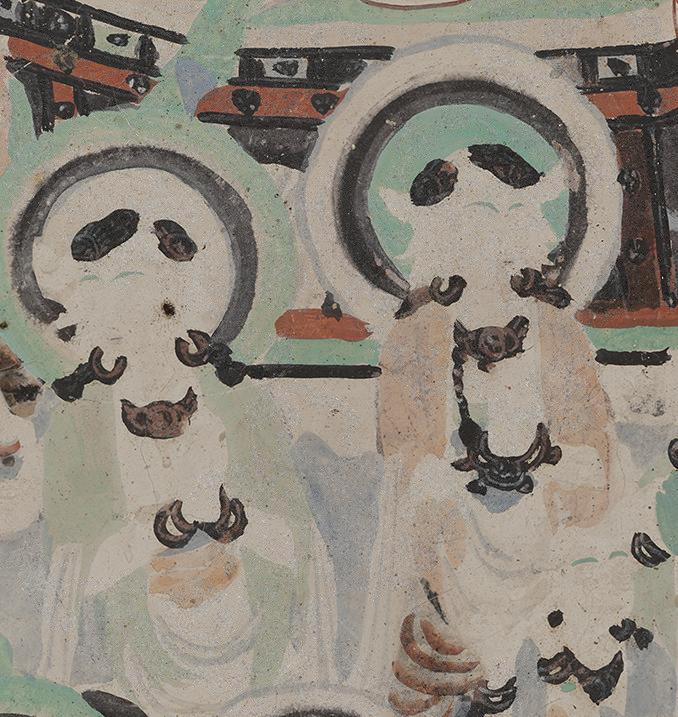

Supplement: Supplementary file 1 [file sensors-21-02091-s001.zip › smartsensors_supplementary_data/nazerietal-model-results/146_masked_1701441_2.jpg]

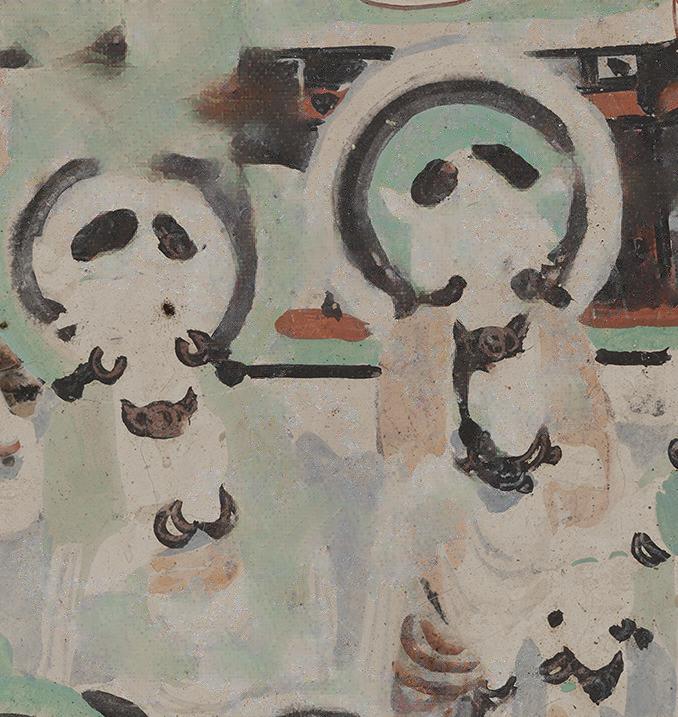

Supplement: Supplementary file 1 [file sensors-21-02091-s001.zip › smartsensors_supplementary_data/nazerietal-model-results/146_masked_1701441_3.jpg]
